# *β*-Arylation of oxime ethers using diaryliodonium salts through activation of inert C(sp^3^)–H bonds using a palladium catalyst†
†Electronic supplementary information (ESI) available. CCDC 1057827 and 1048079. For ESI and crystallographic data in CIF or other electronic format see DOI: 10.1039/c5sc03903g


**DOI:** 10.1039/c5sc03903g

**Published:** 2015-11-11

**Authors:** Jing Peng, Chao Chen, Chanjuan Xi

**Affiliations:** a Key Laboratory of Bioorganic Phosphorus Chemistry & Chemical Biology (Ministry of Education) , Department of Chemistry , Tsinghua University , Beijing 100084 , China . Email: chenchao01@mails.tsinghua.edu.cn

## Abstract

An efficient method of selective *β*-arylation of oxime ethers was realized by using a palladium catalyst with diaryliodonium salts as the key arylation reagents. The reaction proceeded smoothly through the activation of inert C(sp^3^)–H bonds to give corresponding ketones and aldehydes. This convenient procedure can be successfully applied to construct new C(sp^3^)–C(sp^2^) bonds on a number of complex molecules derived from natural products and thus serves as a practical synthetic tool for direct late-stage C(sp^3^)–H functionalization.

## Abstract 2

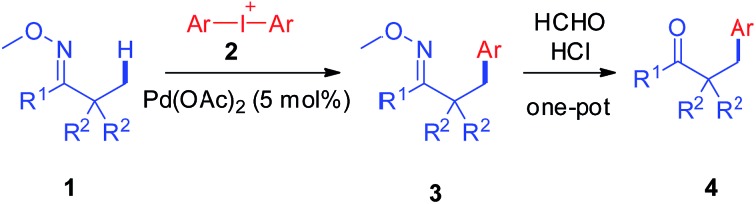
Palladium catalyzed selective *β*-arylation of oxime ethers was realized using diaryliodonium salts as the key arylation reagents.

## Introduction

Arylation *via* direct activation of inert C–H bonds has emerged as a fascinating field, which could provide useful aromatic compounds with high atom- and step-economy.^1^ In the past decade, significant progress has been made in the development of transition-metal catalyzed arylation on C(sp^2^)–H which enables coupling a large range of substrates to various aromatic reagents,^2^ including more challenging work on enantioselective construction of stereo-centers published most recently.2j–l In comparison, arylation on an inert C(sp^3^)–H bond (simple alkyl C–H bond) is much less explored, with a scope of limited substrates reported.^3^ One of the most successful strategies is transition-metal catalyzed-arylation of carboxylate derivatives, including carboxylic acids, esters and amides.^4^ Assisted by big auxiliary groups, alkyl amines could also be selectively arylated on the chain.^5^

**Scheme 1:**
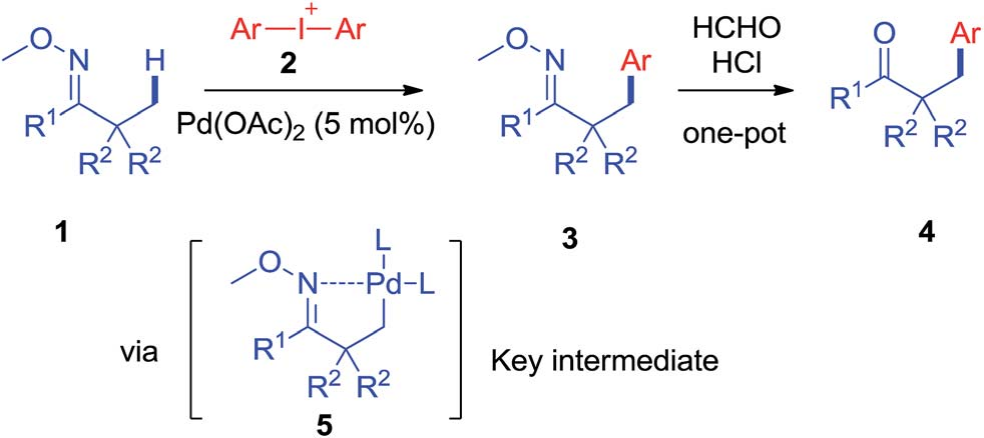
Pd-catalyzed *β*-arylation reaction of oxime ethers *via* C(sp^3^)–H bond activation.

In light of these advances, we were encouraged to develop new arylation reactions through direct activation on alkyl C–H bonds with a wider scope of substrates which offers unprecedented opportunities to efficiently synthesize valuable aromatic compounds.^6^ With regard to chelation-assisted C–H bond functionalization, facile introduction and removal of the directing group could enlarge the scope of substrates from pre-designed molecules and consequently enable the protocol to be applicable to various natural products, generating new and attractive sources for bioactive compounds with high diversity and functionality.^7^ With this ultimate goal in mind, we successfully developed a Pd-catalyzed *β*-arylation reaction on the inert C(sp^3^)–H bond of oxime ethers to give useful products, which could be easily converted to important *β*-aryl ketones, amines and so on.^8^ The reaction proceeds smoothly *via* C(sp^3^)–H activation with diaryliodonium salts as the key coupling partner reagents (Scheme 1).

## Results and discussion

As revealed by known work, the main issues of transition-metal catalyzed functionalization on C(sp^3^)–H bonds not only resulted from the inertness and abundance of C(sp^3^)–H bonds in organic compounds but, more essentially, from the inherent instability of *in situ* generated catalytic metal species, which may easily undergo *β*-hydrogen elimination or side reactions.^9^ To some extent, a good solution is adjusting electronic and coordination effects of the catalytic species, but this generally required additional modification of the substrates.^10^ Hence, a more straightforward solution might be to accelerate the transformation of the catalytic metal species by accomodating the proper coupling reagents with an appropriate chelating group.^11^ With this strategy, we successfully realized the *β*-arylation reaction on the C(sp^3^)–H bond of oxime ethers with Pd(OAc)_2_ as the catalyst. During the study of C–C bond formation on inert C–H bonds, we initiated the investigation with arylating 2-methylcyclohexanone *O*-methyl oxime, which is quantitatively synthesized from *α*-methylcyclohexanone. To begin with, when PhBr or PhI was used as the coupling reagent under various conditions, it always failed to give the desired arylation product, **3aa**, and dehydrogenation side products **1a′** and **1a′′** were observed with the formation of Pd-black. This implied that the putative Pd-species **5** was generated from **1a** with Pd(OAc)_2_, but due to the instability, PhBr or PhI could not be coupled to species **5** before it underwent *β*-hydrogen-elimination. In order to facilitate the desired arylation reaction, Ph_2_I^+^PF_6_^–^, **2a**·PF_6_^–^ (1 equiv.) was chosen as the coupling reagent and **3aa** was formed, albeit in 17% yield (Table 1).^12^ The addition of a base slightly increased the yield of **3aa**, but the best yield was only 27% when Ag_2_CO_3_ (2 equiv.) was employed. By adding pivalic acid, the starting materials were fully converted to produce **3aa** with 50% yield.^13^ In order to further accelerate the transfer step of the phenyl group, we added the polar solvent, hexafluoroisopropanol (HFIP) to the reaction, resulting in an increase to 82% yield of **3aa**. The use of **2a**·OTf^–^ gave an even better result (87%, entry 17, isolated in 83%), while **2a**·BF_4_^–^ failed to generate **3aa**. For further modification, when the reaction was completed under the optimized condition, entry 17, the mixture was treated with formaldehyde in an acidic system,^14^ and 2-benzyl cyclohexanone, **4aa**, was isolated in 80% yield (for experimental details, see ESI†). In accordance with the initial proposal, ketoximes and ketones can be easily interconverted, making this method an efficient approach to *β*-aryl ketones.

**Table 1:** Optimization of reaction conditions for **3aa**^*a*^

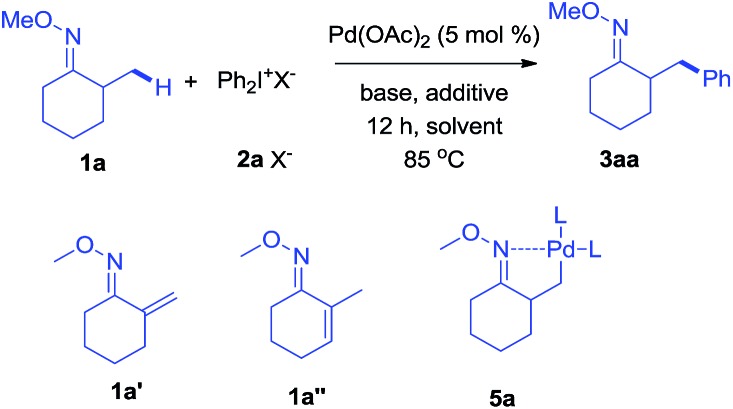				
Entry	Solvent	Base (equiv.)	Additive (equiv.)	Yield^*b*^
1	DCE	None	None	Trace
2	DCE	None	None	17%
3	CH_3_CN	None	None	N.R.
4	DMSO	None	None	N.R.
5	EtOH	None	None	N.R.
6	DCE	K_2_CO_3_ (1)	None	10%
7	DCE	NaHCO_3_ (1)	None	15%
8	DCE	Na_2_CO_3_ (1)	None	8%
9	DCE	Ag_2_CO_3_ (1)	None	23%
10	DCE/*t*-butanol (4 : 1)	Ag_2_CO_3_ (2)	None	27%
11	DCE	Ag_2_CO_3_ (2)	PivOH (0.3)	44%
12	DCE	Ag_2_CO_3_ (2)	PivOH (0.6)	50%
13	DCE/*t*-butanol (4 : 1)	Ag_2_CO_3_ (2)	PivOH (0.6)	57%
14	DCE/HFIP (3 : 1)	Ag_2_CO_3_ (2)	PivOH (0.6)	82%
15	DCE/HFIP (1 : 1)	Ag_2_CO_3_ (2)	PivOH (0.6)	40%
16	DCE/HFIP (1 : 1)	Ag_2_CO_3_ (2)	PivOH (0.6)	51%
17^*c*^ ^,^^*d*^	DCE/HFIP (3 : 1)	Ag_2_CO_3_ (2)	PivOH (0.6)	87%
18^*d*^	DCE/HFIP (3 : 1)	Ag_2_CO_3_ (2)	None	Trace

^*a*^Reaction conditions: **1a** (0.25 mmol), **2a**·X^–^(0.25 mmol), solvent (2 mL).

^*b*^Determined by NMR using trichloroethylene as an internal.

^*c*^ **2a**·OTf^–^was used.

^*d*^The reaction was quenched after 5 hours.

**Scheme 2:**
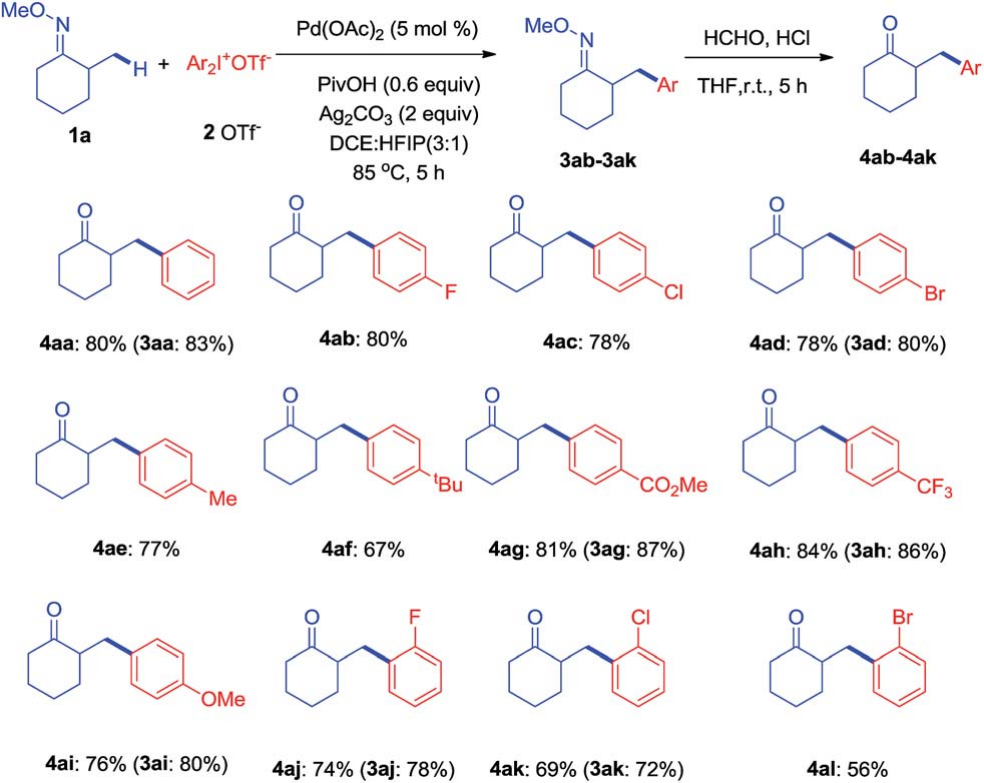
Scope of diaryliodonium salts to form desired *β*-arylated products.

With the optimized conditions established, the scope of diaryliodonium salts was examined for *β*-arylation of **1a**. As shown in Scheme 2, diaryliodonium salts with a range of substituents were efficiently coupled using these conditions. Most of the products were isolated in the ketone form (**4**) rather than oxime ethers (**3**), since ketones were considered to be more synthetically useful. The diaryliodonium triflates with F^–^, Cl^–^ and Br^–^ substituents at the *para* position (**2b–2d**) reacted smoothly with **1a**, generating corresponding products (**4ab–4ad**) in good yields. The use of methyl and ^*t*^butyl substituted diphenyliodonium triflates (**2e–2f**) and **1a** provided products (**4ae–4af**) in slightly lower yields. Diaryliodonium triflates bearing a strong electron-withdrawing group (–CO_2_Me and –CF_3_) or strong electron-donating group (–OMe) also yielded desired products (**4ag–4ai**) while **3ah** was obtained under a lower temperature of 70 °C. The reaction of **1a** with *ortho*-substituted diaryliodonium triflates (**2j–2l**) also afforded expected products (**4aj–4al**) in satisfactory yields. However, the use of asymmetric diaryliodonium salts Ar–I^+^-MesX^–^ failed to give products. A number of selected oximes (**3aa**, **3ad**, **3 ag**, **3ah**, **3ai**, **3aj**, and **3ak**) were isolated to demonstrate the original efficiency.

**Scheme 3:**
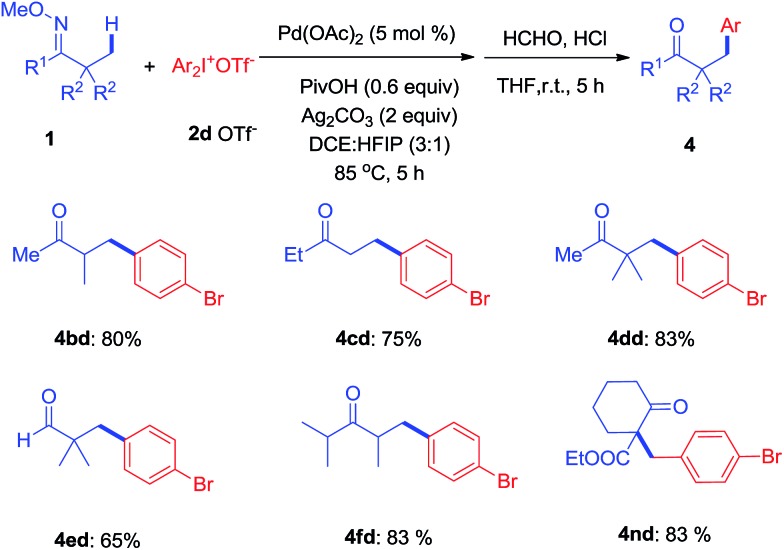
*β*-Arylation of selected oxime ethers with **2d**.

To further investigate, a series of substituted acylic oxime substrates were examined to explore the regioselectivity of the arylation with di(4-bromophenyl)iodonium **2d**. As shown in Scheme 3, ketoximes **1b–1d** all reacted with **2d** to give mono-arylated products at the *α*-methyl group in good yields, while the generation of bi-arylated compounds remained at trace amounts. The use of aldoxime **1e** only produced **4ed** in moderate yield while the bulky ketoxime **1f** with four *α*-methyl groups afforded the mono-arylated product **4fd** in good yield. In addition, we examined the effect of substituents on cyclohexyl oxime ethers and **4nd** was isolated in 83% yield from **1n**.

**Scheme 4:**
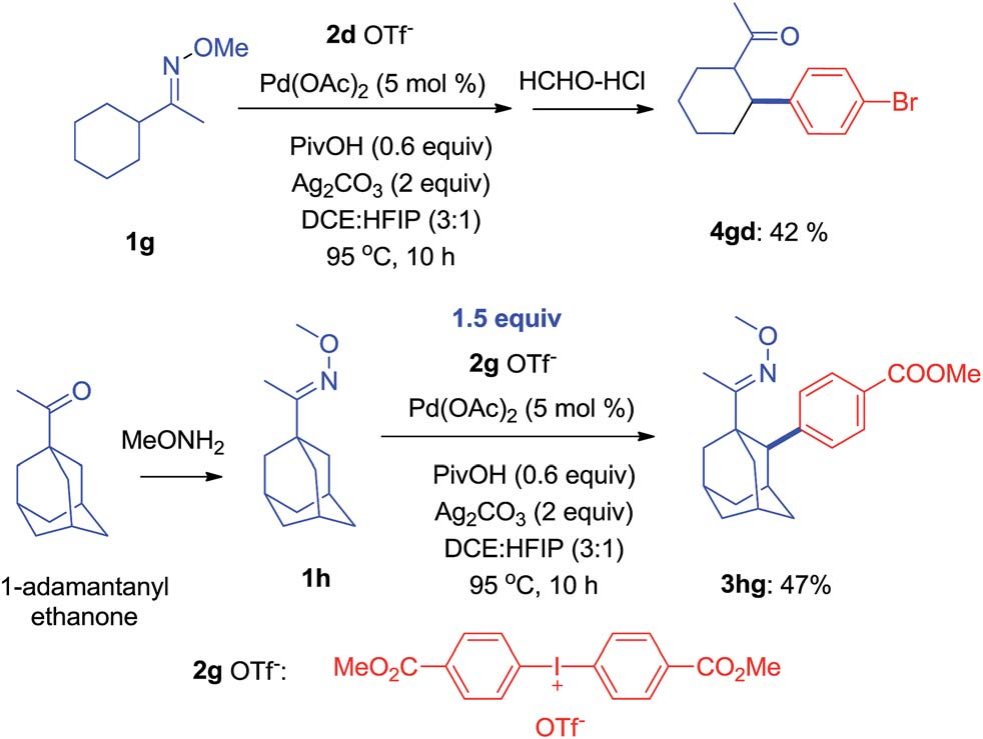
The activation of methylene C–H bonds to form C–C bonds.

It is known that the activation of methylene C–H bonds is harder than of methyl C–H bonds in transition-metal catalyzed reactions because C–H bonds in the methylene position are more hindered.^15^ As shown herein, some of the ketoximes with the appropriate configuration, for instance **1g**, could successfully be arylated with **1d** to give **4gd** in 42% yield. Similarly, it also worked with an oxime ether containing a 1-adamantanyl group, **1h**, to produce **3hg** in 47% yield (Scheme 4).

**Scheme 5:**
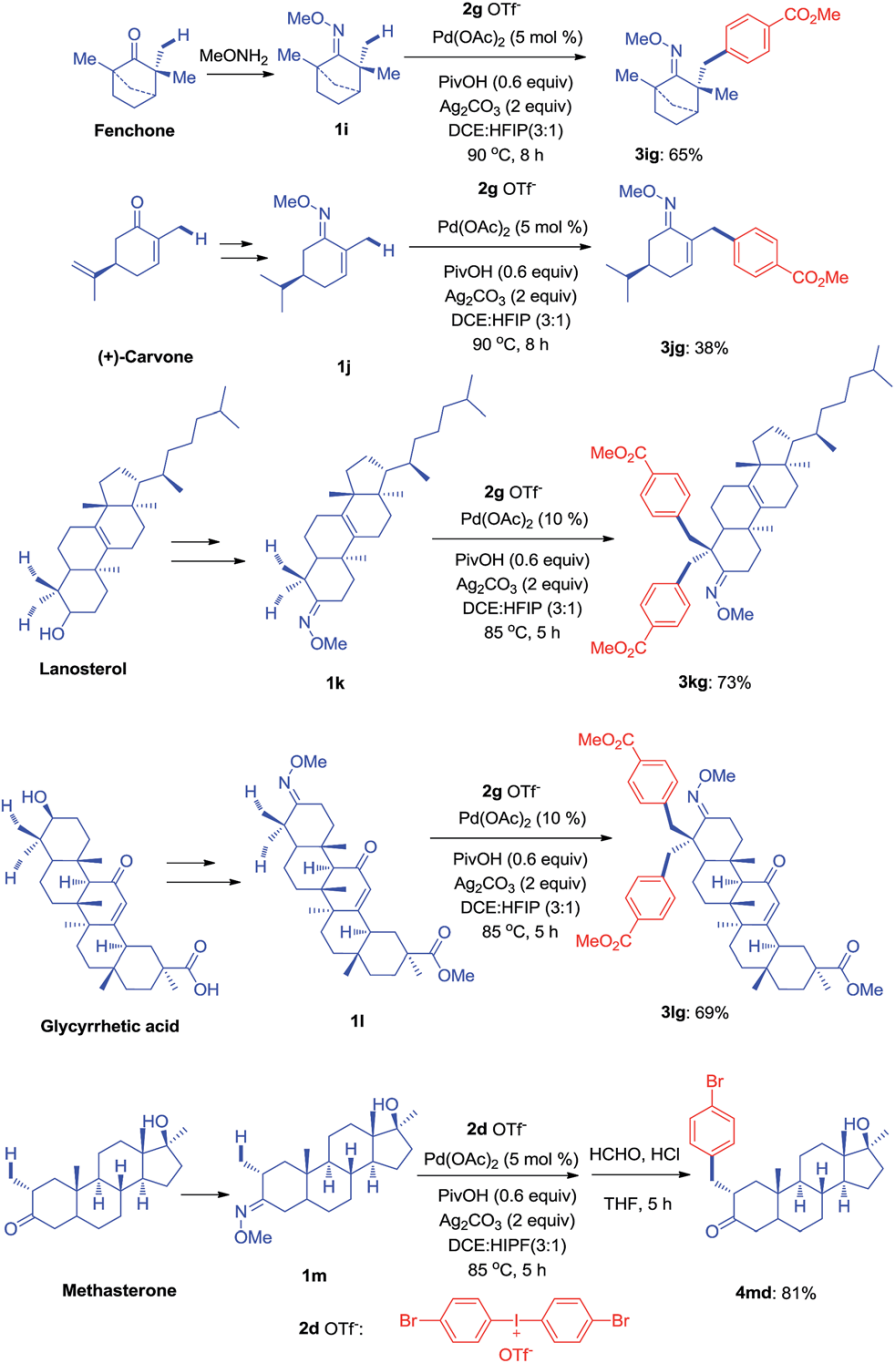
Modification of complex molecules derived from natural products (see ESI† for experimental details).

**Fig. 1:**
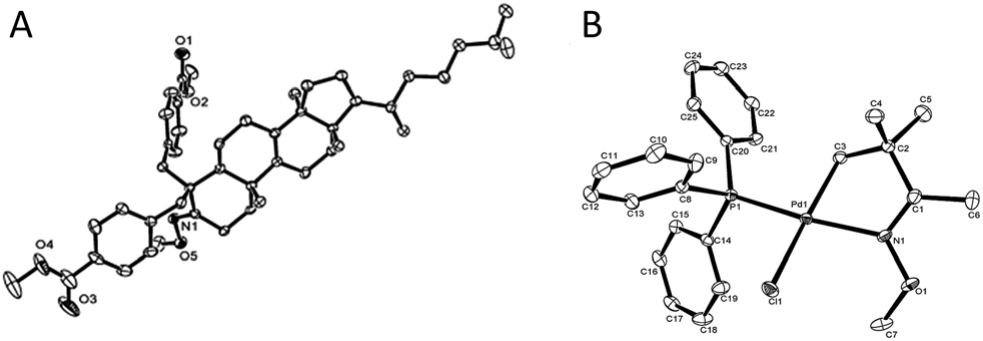
A (left), Crystal structure of compound **3kg**; B (right), crystal structure of palladation intermediate **6** (see ESI† for detailed date of the crystals).

**Scheme 6:**
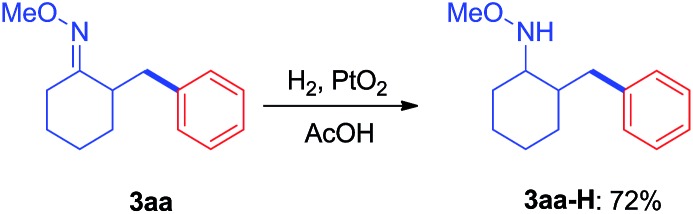
Hydrogenation of **3aa** to generate **3aa-H**.

Since the direct modification of natural products at a normally inert position has attracted much attention due to the potential to access new bioactive compounds from “starmolecules”,1a,^16^ some complex substrates with natural product backbones were examined in our new arylation reactions for reactivity, selectivity and tolerance of functional groups (Scheme 5). Oxime **1i**, derived from naturally-occurred fenchone which contains three methyl groups, could be selectively arylated on an exo-methyl group with **2g** under standard conditions, giving **3ig** in 65% yield. Naturally abundant in many essential oils, (+)-carvone can be easily converted to *α*,*β*-unsaturated ketone oxime with *α*-methyl group **1j**. This can then can be arylated with **2g** to give **3jg** under the synthetic method established, albeit in 38% yield. However, with 1 equivalent of diaryliodonium salt **2g**, the mono-arylated product of **1k** derived from lanosterol was observed as two inseparable isomers in a relevantly low yield. Alternatively, when 2 equivalents of **2g** were employed in the transformation, the bis-arylated product **3kg** was successfully obtained in 73% isolated yield. The structure of **3kg** was confirmed by XRD analysis, shown in Fig. 1A. *β*-Glycyrrhetinic acid is a major metabolite of glycyrrhizin, one of the main constituents of licorice, and has been shown to exhibit anti-ulcerative, anti-inflammatory, and immunomodulatory properties. Substrate **1l**, derived from glycyrrhetinic acid, could also be bis-arylated on both methyl C–H bonds to form **3lg** with 69% isolated yield. Besides natural product-like compounds, the synthetic reagent, methasterone was converted to oxime **1m**, which was then successfully arylated with **2d** to give **4md** in high yield, with the hydroxyl group unchanged during the reaction. In addition, oxime ethers can be easily converted to corresponding amines which are useful building blocks with a range of potential applications (Scheme 6).^17^

**Scheme 7:**
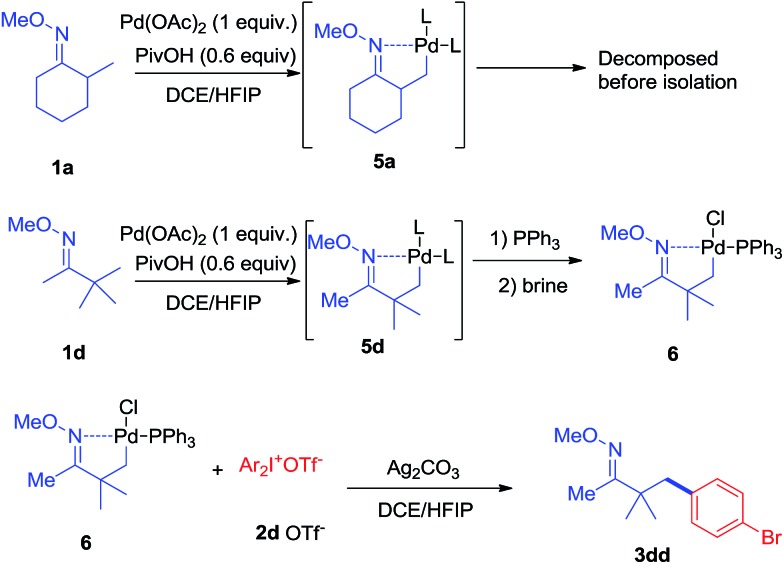
Preparation of the palladation intermediate and its reaction with **2d**.

In terms of the mechanism study, we proposed that the process was initiated by a cyclometalated complex.^18^ Using **1a** as the starting backbone, the isolation of the palladation intermediate always failed due to the strong tendency of *β*-H elimination of **5a**. When treating **1d** with Pd(OAc)_2_, the existence of palladation intermediate **5d** was proved. By converting **5d** to **6** (Scheme 7),^19^ the crystal structure of **6** was identified, indicating that palladium was bound with CH_2_ and oxime-nitrogen atom as a cyclometalation species (Fig. 1B). In order to confirm the catalytic competency, complex **6** was treated with 1 equivalent of **2d** and 2 equivalents of Ag_2_CO_3_, analogously to the standard reaction conditions, and **3dd** was observed in 80% yield by *in situ* NMR (Scheme 7).

**Scheme 8:**
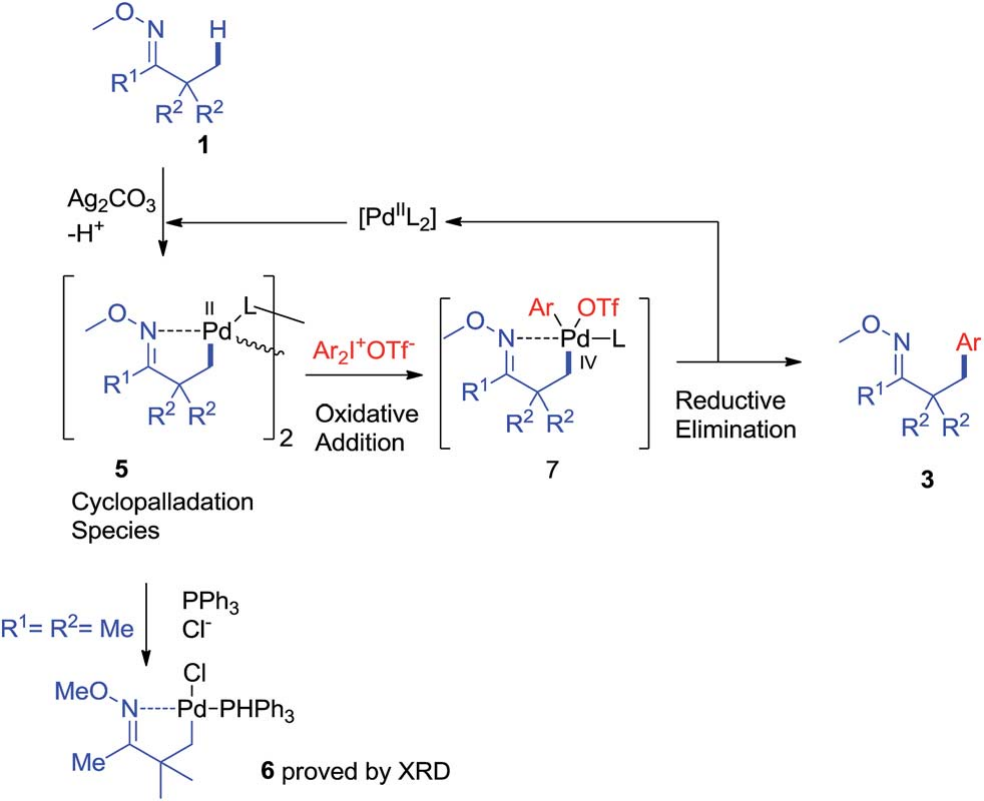
A plausible mechanism.

Based on the above results and literature reports, a plausible mechanism is proposed in Scheme 8. First, the reaction of oxime ether **1** with Pd^II^ species (Pd(OAc)_2_ or other palladium salts generated *in situ*) would produce cyclopalladation species **5**. And the oxidative addition of diaryliodonium salt **2** to **5** would afford Pd^IV^ intermediate **7**.^11^ The reductive elimination of **7** would give product **3**, and release the Pd^II^ species into the catalytic cycle.

## Conclusions

In summary, we have developed a novel *β*-arylation reaction on inert C(sp^3^)–H bonds of oxime ethers. The reaction offers new opportunities to prepare useful *β*-arylated oximes from/to ketones and aldehydes *via* simple transformation with good efficiency and step-economy. The easy manipulation and good tolerance of functional groups enable the method to be used to modify many complex compounds derived from natural backbones. Further investigations on the scope, mechanism, and synthetic application of this new reaction are under way in our laboratory.

## Supplementary Material

Supplementary information

Crystal structure data
