# Supplementary material for: β-Arylation of oxime ethers using diaryliodonium salts through activation of inert C(sp)–H bonds using a palladium catalyst
Source: Chem Sci. 2015 Nov 11;7(2):1383–7. doi: 10.1039/c5sc03903g (PMC5975923; doi:10.1039/c5sc03903g)
Supplement: Supplementary file 1 [file SC-007-C5SC03903G-s001.pdf]

Electronic Supplementary Material (ESI) for Chemical Science.
This journal is © The Royal Society of Chemistry 2015

# *Supporting Information*

# *β*-Arylation of Oxime Ethers Using Diaryliodonium Salts through Activation of Inert $C_{(sp3)}$-H Bond with Palladium Catalyst

*Jing Peng, Chao Chen,* Chanjuan Xi*

Key Laboratory of Bioorganic Phosphorus Chemistry & Chemical Biology (Ministry of Education), Department of Chemistry, Tsinghua University, Beijing 100084, China

**Table of Contents**…………………………………………………………………1

**I. General Method**…………………………………………………………………2

**II. Experimental Procedures for the Preparation of Starting Material**………3

**III. Experimental Procedure for the Optimization Study**……………………10

**IV. Experimental Procedure for the sp$^3$ C−H Arylation**……………………12

**V. Experimental Procedure of hydrogenation**…………………………………26

**VI. Palladium Complexes**…………………………………………………………27

**VII. Discussions on Negative Results**……………………………………………29

**VIII. Copies of $^1$H and $^{13}$C NMR spectra**……………………………………30

**IX. References**……………………………………………………………………70

## I. General Comments

All the reactions were carried out in a pre-dried screwcapped tube with a Teflon-lined septum under $N_2$ atmosphere. Diaryliodonium salts except $Ph_2IPF_6$ and $(4\text{-}^t\text{Bu-Ph})_2IOTf$ were prepared according to the literatues[1]. Column chromatography was performed on silica gel (particle size 10-40 μm, Ocean Chemical Factory of Qingdao, China). $^1$H NMR and $^{13}$C NMR spectra were recorded on a JEOL AL-300MHz, AL-400MHz or AL-600MHz spectrometer at ambient temperature with $CDCl_3$ as the solvent. Chemical shifts (δ) were given in ppm, referenced to the residual proton resonance of $CDCl_3$ (7.26), to the carbon resonance of $CDCl_3$ (77.16). Coupling constants (*J*) were given in Hertz (Hz). The term m, dq, q, t, d, s referred to multiplet, doublet quartet, quartet, triplet, doublet, singlet.

HRMS experimets were carried out on a Thermo Scientific LTQ Orbitrap Discovery (Bremen, Germany). Melting points were gained by mini-Lamp. MS data were monitored by GC-MS. The reaction progress was monitored by TLC and GC-MS if applicable.

## II. Experimental Procedures for the Preparation of Starting Materials

### 1. General Procedure for Preparing Oximes (1a-1h, 1m)[2]

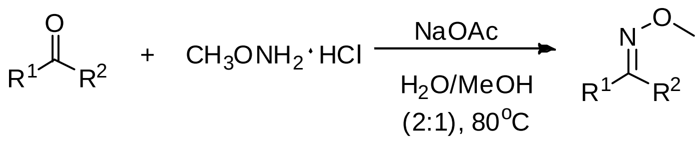


Methoxylamine hydrochloride (1.5 equiv) and NaOAc (2.5 equiv) were placed in a schlenk tube and charge with $N_2$. Solvent, $H_2O$ (10 ml)/MeOH (5 mL), were added into the tube alongside with ketone (5 mmol) and the mixture was stirred at 80°C for 5h. After cooling down to room temperature, dichloromethane (10 mL) and water (10 mL) were added. The aqueous layer was extracted with dichloromethane and the combined organic extracts were dried over $MgSO_4$, filtered, and concentrated under reduced pressure.

**Note:** Aimed ketoximes were obtained quantitively after concentration under reduced pressure without any further purification.

### 2. Procedure for Preparing Compound 1i [2]

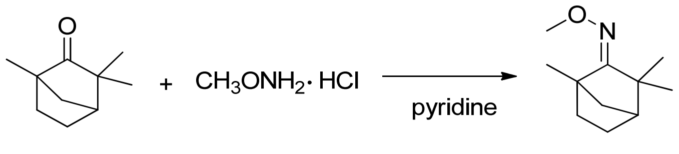


Fenchone

A stirred solution of fenchone (5.0 mmo, 760 mg) and methoxylamine hydrochloride (5.5 mmol, 456.5 mg) in pyridine (10 mL) was heated at 115°C for overnight. Pyridine was removed under reduced pressure. The residue was diluted with diethyl ether (10 mL) and then washed with water (10 mL). The organic phase was dried over $MgSO_4$, filtered, and evaporated under reduced pressure. The residue was purified by column chromatography on silica gel to give desired **1i** (500 mg, 55%).

### 3. Procedure for Preparing Compound 1j [3]

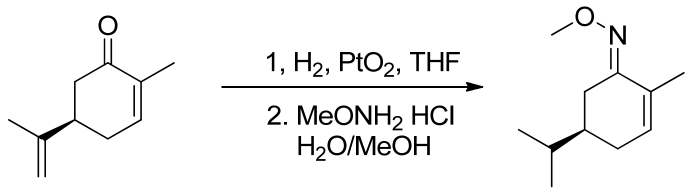


(+)-Carvol

$PtO_2$ (0.05 mmol, 11 mg) in 50 mL THF were placed in a round bottom flask. (+)-Carvol (10 mmol, 1.56 mL) was slowly added and the mixture was stirred at room temperature under

an atmosphere of hydrogen gas (balloon). Progress was monitored by GC/MS with full conversion achieved after 5.5 h. The resulting mixture was filtered through celite and washed with EtOAc (10ml×3). The filtrate was evaporated under reduced pressure and the pure 5-isopropyl-2-methylcyclohex-2-enone were obtained by column chromatography on silica gel (Hexane/EtOA = 1/50). (1.31 g, 86%).

The aimed product (**1j**) (1.45 g, 93%) were prepare from 5-isopropyl-2-methylcyclohex-2-enone according to the general procedure for preparing oximes above.

## 4. Procedure for Preparing Compound 1k [3,4]

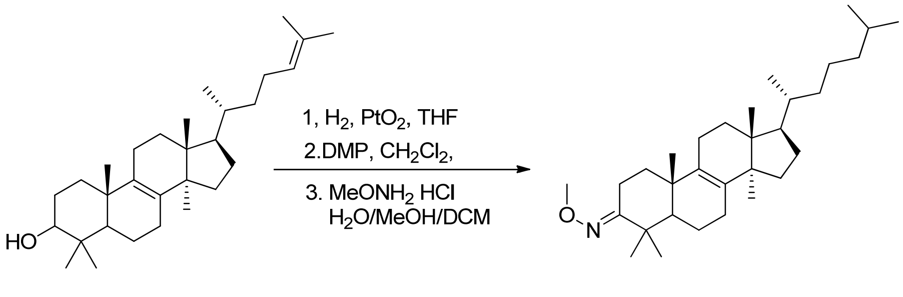


$PtO_2$ (0.05 mmol, 11 mg) in 50 mml THF were placed in a round bottom flask and Lanosterol (10 mmol, 4.26 g) was slowly added. The mixture was stirred at room temperature under an atmosphere of hydrogen gas (balloon). Progress was monitored by GC/MS with full conversion achieved after 6h. The resulting mixture was filtered through celite and washed with EtOAc. The filtrate was evaporated under reduced pressure and the residue was used without further purification. To a stirred solution of the residue in dichloromethane (100 mL) was added Dess-Martin periodinane (15 mmol, 6.36 g). After stirring for one hour at room temperature, 100μl water was added into the mixture to accelerate the conversion. The reaction was quenched after another 3h. The resulting mixture was extracted with EtOAc (50 ml×3) while the organic layers were combined, dried over $MgSO_4$, filtered, and concentrated. The residue was purified by column chromatography on silica gel (Hexane/EtOA = 1/30) to give the ketone. (3.00 g, 70%) .

Hydroxylamine hydrochloride (1.58 g, 18.8 mmol) and NaOAc (2.51 g, 30.8 mmol) were added to the above ketone (3g, 7 mmol) in $H_2O$ (20 mL)/MeOH (10 mL)/DCM (5 mL) and the mixture was stirred at 75 $^{o}$C for 4 h. After cooling down to room temperature, EtOAc (15 mL) and water (10 mL) were added. The aqueous layer was extracted with EtOAc (15 mL x 3) and the combined organic extracts were dried over $MgSO_4$, filtered, and concentrated under reduced pressure. The residue was purified by column chromatography on silica gel (Hexane/EtOAc = 50/1) to afford ketoxime **1k** (2.96g, 93%).

## 5. Procedure for Preparing Compound 1l [5,6]

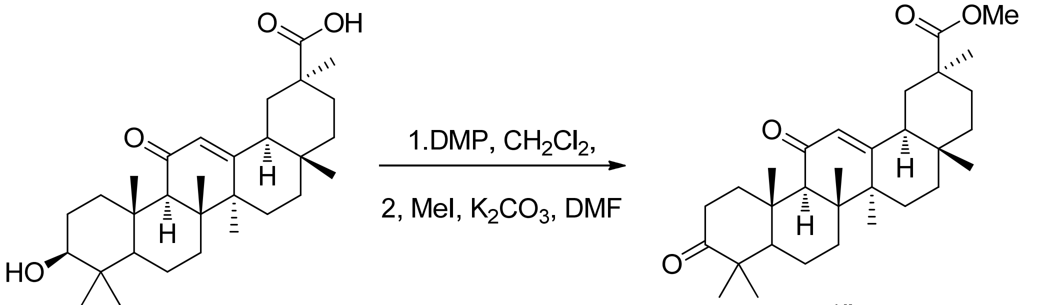


1l'

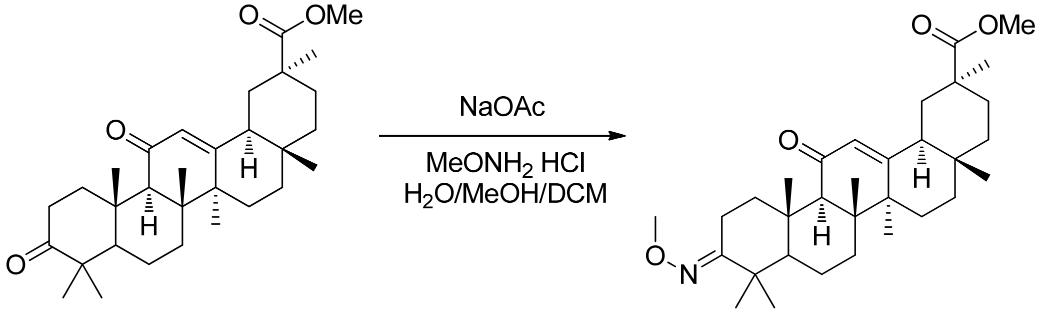


1l'

1l

To a solution of Glycyrrhetinic Acid (2.35 g, 5 mmol) in dichloromethane (50 mL) was added Dess-Martin periodinane (5.5 mmol, 2.33 g). After stirring for 1h at room temperature, 100μl water was added into the mixture to facilitate the conversion. The reaction was quenched after another 3h and the resulting mixture was extracted with EtOAc (30 ml×3). The organic layers were combined, dried over $MgSO_4$, filtered and concentrated without further purification to give the corresponding ketone [6] quantitively.

Solid $K_2CO_3$ (3.12 g, 22.5 mmol) as well as neat MeI (4.67 mL, 75.3 mmol) were added to a room temperature DMF solution (30 mL) of glycyrrhetic acid (2.35 g, 5 mmol). After stirring for 8h, solution was filtered followed by evaporating filtration. The residual DMF was portioned between $H_2O$ (200 mL) and EtOAc (200 mL) while aqueous layer was separated and extracted with EtOAc. Then the combined organic layers were washed with 5 % $Na_2S_2O_3$, saturated $NaHCO_3$, brine and then dried ($Mg_2SO_4$). Concentration afforded the crude ester that was purified by chromatography (1:20 EtOAc/hexanes) to afford corresponding ester (1l') as a white solid (1.70 g, 73%).[5]

Hydroxylamine hydrochloride (677 mg, 8.1 mmol) and NaOAc (1.08 g, 13.2 mmol) were added to the above ketone (1.45g, 3 mmol) in $H_2O$ (10 mL)/MeOH (5 mL)/DCM (2.5 mL) and the mixture was stirred at 75 $^oC$ for 4 h. After cooling down to room temperature, EtOAc (10 mL) and water (10 mL) were added. The aqueous layer was extracted with EtOAc (15 mL x 3) and the combined organic extracts were dried over $MgSO_4$, filtered, and concentrated under reduced pressure.**1l** can be obtained quatitively after concentration.

## 6. Spectroscopic Data of Prepared Oximes

***(E)*-2-methylcyclohexanone *O*-methyl oxime (1a)**: colorless liquid.

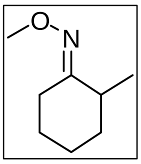

$^{1}$H NMR (400 MHz, CHLOROFORM-D) δ 3.81 (s, 3H), 2.84 – 2.71 (m, 1H), 2.36 – 2.27 (m, 1H), 2.11 – 2.03 (m, 1H), 1.88 – 1.76 (m, 1H), 1.75 – 1.64 (m, 2H), 1.54 – 1.44 (m, 2H), 1.40 – 1.33 (m, 1H), 1.10 (d, *J* = 6.8 Hz, 3H). $^{13}$C NMR (101 MHz, CHLOROFORM-D) δ 163.0, 61.1, 36.8, 35.4, 26.3, 24.1, 24.0, 17.3. EI-MS: 141 ($M^+$)

**pentan-3-one *O*-methyl oxime (1b)**: colorless liquid

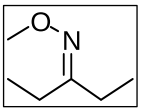

$^{1}$H NMR (301 MHz, CHLOROFORM-D) δ 3.79 (s, 3H), 2.28 (q, *J* = 7.6 Hz, 2H), 2.17 (q, *J* = 7.6 Hz, 2H), 1.07 (t, *J* = 5.5 Hz, 2H), 1.03 (t, *J* = 5.5 Hz, 1H). $^{13}$C NMR (76 MHz, CHLOROFORM-D) δ 163.5, 61.1, 27.1, 21.2, 11.3, 10.5. EI-MS: 115 ($M^+$)

**3-methylbutan-2-one *O*-methyl oxime (1c)**: colorless liquid; obtained as a mixture of two isomers in a ratio E/Z=6:1

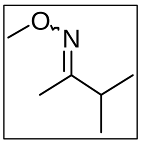

major isomer (***(E)*3-methylbutan-2-one *O*-methyl oxime**): $^{1}$H NMR (400 MHz, CHLOROFORM-D) δ 3.8 (s, 3H), 2.55 – 2.37 (m, 1H), 1.73 (s, 3H), 1.04 (d, *J* = 6.9, Hz, 6H).$^{13}$C NMR (101 MHz, CHLOROFORM-D) δ 162.0, 61.1, 34.3, 20.0, 10.8. EI-MS: 115 ($M^+$)

***(E)*-3,3-dimethylbutan-2-one *O*-methyl oxime (1d)**: colorless liquid

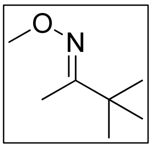

$^{1}$H NMR (400 MHz, CHLOROFORM-D) δ 3.81 (s, 3H), 1.77 (s, 3H), 1.10 (s, 9H). $^{13}$C NMR (101 MHz, CHLOROFORM-D) δ 163.4, 61.1, 37.1, 27.8 10.5.
EI-MS: 129 ($M^+$)

***(E)*-pivalaldehyde *O*-methyl oxime (1e):** colorless liquid

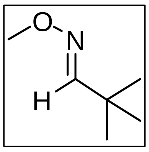

$^{1}$H NMR (301 MHz, CHLOROFORM-D) δ 7.29 (s, 1H), 3.80 (s, 3H), 1.09 (s, 9H).$^{13}$C NMR (76 MHz, CHLOROFORM-D) δ 158.3, 61.2, 33.6, 27.7
EI-MS: 115 ($M^+$)

**2,4-dimethylpentan-3-one *O*-methyl oxime (1f)**: colorless liquid

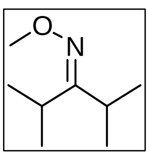

$^{1}$H NMR (400 MHz, CHLOROFORM-D) δ 3.75 (s, 3H), 2.98 (m, 1H), 2.48 (m, 1H), 1.10 (d, *J* = 7.1 Hz, 6H), 1.07 (d, *J* = 6.9 Hz, 6H). $^{13}$C NMR(101 MHz, CHLOROFORM-D) δ 168.5, 61.1, 31.2, 28.1, 21.3, 19.1.
EI-MS: 143 ($M^+$)

**1-cyclohexylethanone *O*-methyl oxime (1g)** : colorless liquid;obtained as a mixture of two isomers in a ratio E/Z=10:1

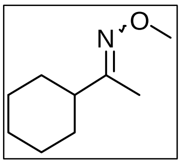


major isomer ( ***(E)*-1-cyclohexylethanone *O*-methyl oxime** ): $^1$H NMR (301 MHz, CHLOROFORM-D) δ 3.77 (s, 3H), 2.21 – 2.03 (m, 1H), 1.77 – 1.62 (m, 8H), 1.31 – 1.14 (m, 5H).$^{13}$C NMR (76 MHz, CHLOROFORM-D) δ 161.4, 61.0, 44.5, 30.2, 26.1, 11.6. EI-MS: 155 ($M^+$)

***(R,E)*-5-isopropyl-2-methylcyclohex-2-enone *O*-methyl oxime (1j)**: colorless liquid

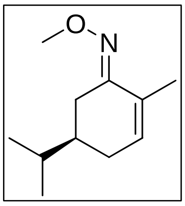


$^1$H NMR (400 MHz, CHLOROFORM-D) δ 5.98 (d, *J* = 5.7 Hz, 1H), 3.90 (s, 3H), 3.14 – 3.01 (m, 1H), 2.25 – 2.09 (m, 1H), 1.91 – 1.84 (m, 1H), 1.82 (s, 3H), 1.79 – 1.71 (m, 1H), 1.56 – 1.42 (m, 2H), 0.93 – 0.89 (m, 6H). $^{13}$C NMR (101 MHz, CHLOROFORM-D) δ 157.0, 133.1, 130.5, 61.8, 39.7, 32.3, 28.8, 26.7, 19.97 (s), 19.8, 17.8. EI-MS: 181 ($M^+$). $[\alpha]_D^{23}$-12.6$^o$ (1, DCM)

**1,3,3-trimethylbicyclo[2.2.1]heptan-2-one *O*-methyl oxime (1i)**: Pale orange liquid, obtained as a mixture of two isomers in a ratio E/Z=12:1

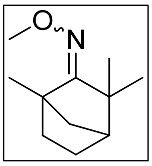


major isomer: ***(E)*-1,3,3-trimethylbicyclo[2.2.1]heptan-2-one *O*-methyl oxime:** $^1$H NMR (400 MHz, CHLOROFORM-D) δ 3.71 (s, 3H), 1.78 – 1.71 (m, 2H), 1.69 – 1.64 (m, 1H), 1.51 (m, 2H), 1.43 – 1.38 (m, 1H), 1.29 (m, 1H), 1.22 (s, 3H), 1.20 (s, 3H), 1.18 (s, 3H). $^{13}$C NMR (101 MHz, CHLOROFORM-D) δ 172.8, 61.1, 50.0, 48.7, 44.7, 43.5, 34.5, 25.4, 23.5, 22.6, 17.3. EI-MS: 181 ($M^+$). $[\alpha]_D^{23}$-23.6$^o$ (1, DCM)

***(E)*-1-((3r,5r,7r)-adamantan-1-yl)ethanone *O*-methyl oxime (1h):** pale orange liquid

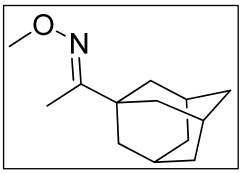


$^1$H NMR (400 MHz, CHLOROFORM-D) δ 3.81 (s, 3H), 2.01 (s, 3H), 1.76 – 1.64 (m, 15H) $^{13}$C NMR (101 MHz, CHLOROFORM-D) δ 163.7, 61.1, 39.6, 39.0, 36.9, 28.3, 9.5. EI-MS: 207 ($M^+$)

**(*E*)-ethyl 2-(methoxyimino)-1-methylcyclohexanecarboxylate (1n):** Pale yellow liquid.

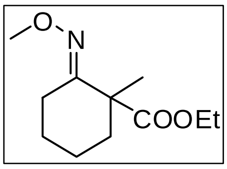


$^1$H NMR (400 MHz, CHLOROFORM-D) δ 4.23 – 4.07 (m, 2H), 3.82 (s, 3H), 3.19 – 3.03 (m, 1H), 2.38 – 2.28 (m, 1H), 1.90-1.82 (m, 1H), 1.77 – 1.69 (m, 1H), 1.69-1.62 (m, 1H), 1.51 – 1.42 (m, 1H), 1.42 – 1.33 (m, 2H), 1.32 (s, 3H), 1.23 (t, *J* = 7.1 Hz, 3H). $^{13}$C NMR (101 MHz, CHLOROFORM-D) δ 175.0, 159.7, 61.4, 61.0, 49.9, 38.2, 25.7, 23.6, 23.0, 22.7, 14.3. EI-MS: 213 ($M^+$)

**(8R,9S,10S,13S,14S,17S,E)-17-hydroxy-2,10,13,17-tetramethyltetradecahydro-1H-cyclopenta[a]phenanthren-3(2H)-one O-methyl oxime (1m):** colorless solid, m.p.156°C-158°C

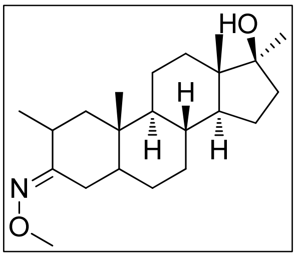


$^{1}$H NMR (301 MHz, CHLOROFORM-D) δ 3.79 (s, 3H), 2.93 (dd, *J* = 14.6, 3.2 Hz, 1H), 2.38-2.30 (m, 1H), 1.85 (dd, *J* = 12.9, 4.8 Hz, 2H), 1.77 – 1.57 (m, 4H), 1.58 – 1.40 (m, 4H), 1.39 – 1.30 (m, 2H), 1.29 – 1.18 (m, 5H), 1.18 (s, 3H), 1.04 (d, *J* = 6.2 Hz, 3H), 0.91 (d, *J* = 5.2 Hz, 3H), 0.88 (s, 2H), 0.83 (s, 3H), 0.64-0.58 (m, 1H).$^{13}$C NMR (76 MHz, CHLOROFORM-D) δ 162.3, 81.8, 61.1, 54.2, 50.7, 48.7 46.2, 45.6, 39.0 36.4, 36.2, 32.9, 31.7, 31.6, 28.5, 27.9, 25.9, 23.3, 20.9, 16.7, 14.1, 12.44. EI-MS: 347 ($M^+$). HRMS (ESI, m/z) calcd for $C_{22}H_{37}NO_2$ $[M+H]^+$: 348.2897 , found: 348.2896. $[\alpha]_D^{23}$+14.3° (1, DCM)

**(10S,13R,14R,17R,E)-4,4,10,13,14-pentamethyl-17-((R)-6-methylheptan-2-yl)-4,5,6,7,10,11,12,13,14,15,16,17-dodecahydro-1H-cyclopenta[a]phenanthren-3(2H)-one O-methyl oxime (1k):** colorless solid, m.p.113°C-115°C

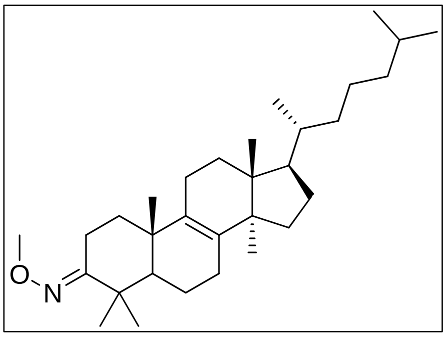


$^{1}$H NMR (600 MHz, CHLOROFORM-D) δ 3.81 (s, 3H), 3.00 (ddd, *J* = 15.3, 5.1, 3.6 Hz, 1H), 2.14 (ddd, *J* = 15.3, 12.8, 5.6 Hz, 1H), 2.07 – 1.99 (m, 4H), 1.96 – 1.89 (m, 1H), 1.81 (ddd, *J* = 12.8, 5.5, 3.7 Hz, 1H), 1.70 (m, 3H), 1.61 – 1.51 (m, 3H), 1.47 – 1.42 (m, 1H), 1.37 – 1.29 (m, 6H), 1.16 (s, 3H), 1.15 – 1.09 (m, 4H), 1.08 (s, 3H), 1.07 (s, 3H), 1.00 – 0.97 (m, 1H), 0.89 (d, *J* = 6.5 Hz, 3H), 0.87 (d, *J* = 3.1 Hz, 3H), 0.86 (d, *J* = 2.2 Hz, 6H), 0.70 (s, 3H). $^{13}$C NMR (76 MHz, CHLOROFORM-D) δ 166.2, 135.0, 134.0, 61.2, 51.6, 50.7, 50.0, 44.6 40.2, 39.7, 37.2, 36.6, 35.9, 31.2, 31.0, 28.4, 28.2, 27.2, 26.6, 24.4, 24.3, 23.4, 23.0, 22.7, 21.2, 19.1, 18.8 18.3, 16.0. EI-MS: 455 ($M^+$). HRMS (ESI, m/z) calcd for $C_{31}H_{3753}NO$ $[M+H]^+$: 456.4200 , found: 456.4199. $[\alpha]_D^{23}$+17.7° (1, DCM)

**(2S,4aS,6aS,6bR,12aS,12bR,14bS)-methyl 2,4a,6a,6b,9,9,12a-heptamethyl-10,13-dioxo-1,2,3,4,4a,5,6,6a,6b,7,8,8a,9,10,11,12,12a,12b,13,14b-icosahydropicene-2-carboxylate (1l')**

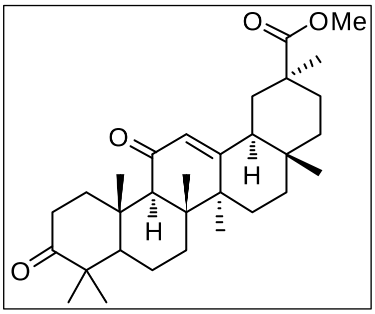


$^{1}$H NMR (400 MHz, CHLOROFORM-D) δ 5.70 (s, 1H), 3.70 (s, 3H), 2.96 (ddd, *J* = 13.4, 7.0, 4.1 Hz, 1H), 2.62 (ddd, *J* = 15.9, 11.0, 7.1 Hz, 1H), 2.36 (ddd, *J* = 15.8, 6.5, 4.1 Hz, 1H), 2.15 – 1.83 (m, 5H), 1.76 – 1.67 (m, 1H), 1.64 (dd, *J* = 24.1, 10.6 Hz, 2H), 1.55 (d, *J* = 8.7 Hz, 2H), 1.51 – 1.41 (m, 3H), 1.39 (s, 3H), 1.32 (dd, *J* = 9.4, 5.1 Hz, 4H), 1.27 (s, 3H), 1.22 (d, *J* = 4.4 Hz, 1H), 1.17 (s, 3H), 1.15 (s, 3H), 1.11 (s, 3H), 1.07 (s, 3H), 0.83 (s, 3H). $^{13}$C NMR (101 MHz, CHLOROFORM-D) δ 216.9, 199.2, 176.7, 169.6, 128.3, 61.0, 55.3, 51.7, 48.3, 47.6, 45.1, 44.0, 43.2, 41.1, 39.6, 37.7, 36.6, 34.1, 32.0, 31.8, 31.0, 28.5, 28.2, 26.5, 26.4, 26.3, 23.3, 21.3, 18.7, 18.4, 15.6.

**(*2S,4aS,6aS,6bR,12aS,12bR,14bS,E*)-methyl 10-(methoxyimino)-2,4a,6a,6b,9,9,12a-heptamethyl-13-oxo-1,2,3,4,4a,5,6,6a,6b,7,8,8a,9,10,11,12,12a,12b,13,14b-icosahydropicene-2-carboxylate (1l):** colorless solid, m.p.251°C-253°C

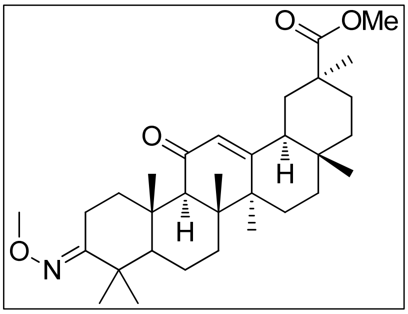


$^1$H NMR (400 MHz, CHLOROFORM-D) δ 5.66 (s, 1H), 3.79 (s, 3H), 3.67 (s, 3H), 2.92 (dt, J = 15.5, 4.2 Hz, 1H), 2.85 – 2.74 (m, 1H), 2.36 (s, 1H), 2.20 (ddd, J = 15.8, 12.8, 5.7 Hz, 1H), 2.11 – 2.03 (m, 1H), 2.02 – 1.94 (m, 2H), 1.90 (d, J = 13.7 Hz, 1H), 1.83 (dd, J = 13.7, 4.0 Hz, 1H), 1.72 – 1.64 (m, 1H), 1.60 (d, J = 13.8 Hz, 2H), 1.48 (t, J = 12.5 Hz, 1H), 1.41 (d, J = 12.4 Hz, 2H), 1.33 (s, 3H), 1.29 (d, J = 9.5 Hz, 2H), 1.22 (s, 3H), 1.20 (s, 1H), 1.16 (s, 3H), 1.13 (s, 6H), 1.05 (s, 3H), 0.98 (m, 3H), 0.79 (s, 3H). $^{13}$C NMR (101 MHz, CHLOROFORM-D) δ 200.0, 177.0, 169.5, 165.8, 128.6, 61.47 (s), 61.1, 55.7, 51.9, 48.5, 45.5, 44.2, 43.4, 41.2, 40.2, 39.2, 37.9, 37.1, 32.6, 32.0, 31.3, 28.7, 28.4, 27.4, 26.6, 26.5, 23.6, 23.4, 18.8, 18.3, 17.9, 15.8. HRMS (ESI, m/z) calcd for $C_{32}H_{49}NO_4$ $[M+H]^+$: 512.3734 , found: 512.3732. $[\alpha]_D^{23}$+49.2° (1, DCM)

**III. Experimental Procedure for the Optimization Study**

*The yield of the product was confirmed by NMR using trichloroethylene as an internal.*

1. Selection for catalyst and preliminary optimization on solvent.

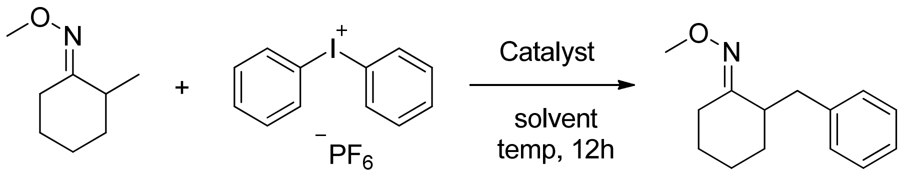


| Entry | Catalyst (5 mmol%) | solvent | Temp (°C) | Yield |
|---|---|---|---|---|
| 1 | CuCl | DCE | 50 | N.R. |
| 2 | CuBr | DCE | 50 | N.R |
| 3 | $Cu(OTf)_2$ | DCE | 50 | N.R |
| 4 | CuCl | Toluene | 50 | N.R |
| 5 | $Pd(OAc)_2$ | DCE | 85 | 17 % |
| 6 | $Pd(OAc)_2$ | DCE | 60 | trace |
| 7 | $Pd(OAc)_2$ | $CH_3CN$ | 80 | N.R. |
| 8 | $Pd(OAc)_2$ | DCM/DMSO | 80 | N.R. |
| 9 | Pd(OAc) | EtOH | 80 | N.R. |
| 10 | $PdCl_2$ | DCE | 80 | N.R. |
| 11 | $Pd(COOCF_3)_2$ | DCE | 80 | N.R. |
| 12 | $PdCl_2(PPh_3)_2$ | DCE | 80 | N.R. |

2. Further optimization for solvent, base, additive and reacting time.

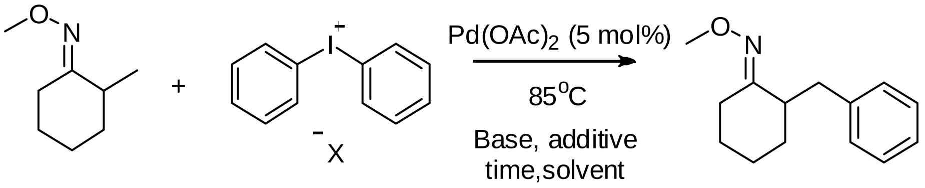


| Entry | Solvent | X | Base | Additive | Time | Yield |
|---|---|---|---|---|---|---|
| 1 | DCE | $PF_6$ | $K_2CO_3$ (1 equiv) | None | 12h | 10% |
| 2 | DCE | $PF_6$ | $NaHCO_3$ (1 equiv) | None | 12h | 15% |
| 3 | DCE | $PF_6$ | $Na_2CO_3$ (1 equiv) | None | 12h | 8% |
| 4 | DCE | $PF_6$ | $Ag_2CO_3$ (1 equiv) | None | 12h | 23% |
| 5 | DCE:tert-Butanol (4:1) | $PF_6$ | $Ag_2CO_3$ (2 equiv) | None | 12h | 27% |
| 6 | DCE | $PF_6$ | $Ag_2CO_3$ (2 equiv) | PivOH (0.3 equiv) | 12h | 44% |
| 7 | DCE:tert-Butanol (4:1) | $PF_6$ | $Ag_2CO_3$ (2 equiv) | PivOH (0.6 equiv) | 12h | 57% |
| 8 | DCE:HFIP(3:1) | $PF_6$ | $Ag_2CO_3$ (2 equiv) | PivOH (0.6 equiv) | 12h | 82% |
| 9 | DCE:HFIP(1:1) | $PF_6$ | $Ag_2CO_3$ (2 equiv) | PivOH (0.6 equiv) | 12h | 40% |
| 10 | DCE:HFIP(1:1) | $PF_6$ | $Ag_2CO_3$ (2 equiv) | PivOH (0.6 equiv) | 12h | 51% |
| 11 | DCE:HFIP(3:1) | OTf | $Ag_2CO_3$ (2 equiv) | PivOH (0.6 equiv) | 5h | 87% |
| 12 | DCE:HFIP(3:1) | $BF_4$ | $Ag_2CO_3$ (2 equiv) | PivOH (0.6 equiv) | 5h | trace |
| 13 | DCE | $PF_6$ | $Ag_2CO_3$ (2 equiv) | PivOH (0.6 equiv) | 5h | 50% |
| 14[a] | DCE:HFIP(3:1) | OTf | $Ag_2CO_3$ (2 equiv) | None | 5h | 30% |
| 15[a] | DCE:HFIP(3:1) | OTf | $Ag_2CO_3$ (2 equiv) | PivOH (0.6 equiv) | 5h | 68% |

[a] 5 mol% of $Pd(OPiv)_2$[11] was employed catalyst instead of $Pd(OAc)_2$

## IV. Experimental Procedure for the $sp^3$ C−H Arylation

### 1. General Procedure for the Pd-Catalyzed Arylation of Oximes.

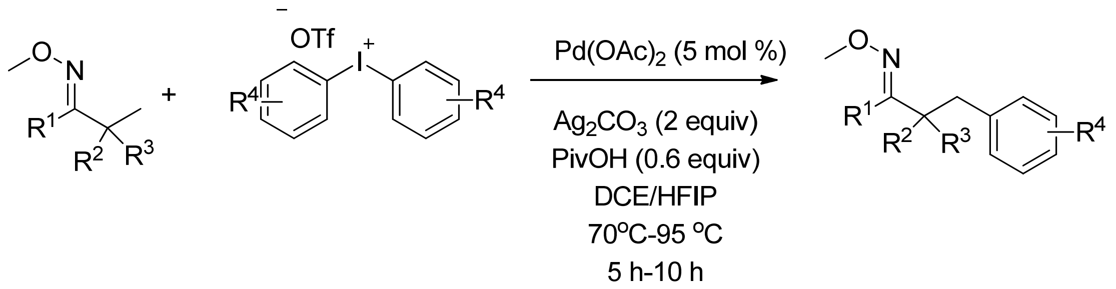


Diaryliodonium salts (0.25 mmol), $Pd(OAc)_2$ (0.0125 mmol, 2.8 mg) along with $Ag_2CO_3$ (0.5 mmol, 137.9 mg) were placed in a schlenk tube. The tube was evacuated and recharged with $N_2$ for 3 times. Mixing solvent of DCE (1.5 ml) and HFIP (0.5 ml) was dropped in under $N_2$ atmosphere. Then appropriate ketoximes (0.25 mmol) and PivOH (0.15 mmol, 16.9 μl) was slowly added to the mixture. The tube was sealed and the mixture was allowed to stir at 70-90 °C for 5h. After completion which was monitored by GC/MS, the mixture was cooled to room temperature, then $NaHCO_3$ aq. (5 mL) was added. The mixture was extracted with ethyl acetate (5 mL x 3) and dried over anhydrous $MgSO_4$. Evaporation of the solvent followed by purification on silica gel (petroleum ether/ethyl acetate 100/1 to 50/1) provided the corresponding ketoximes.

### 2. General Procedure for Obtaining Arylated Ketone and Aldehydes[

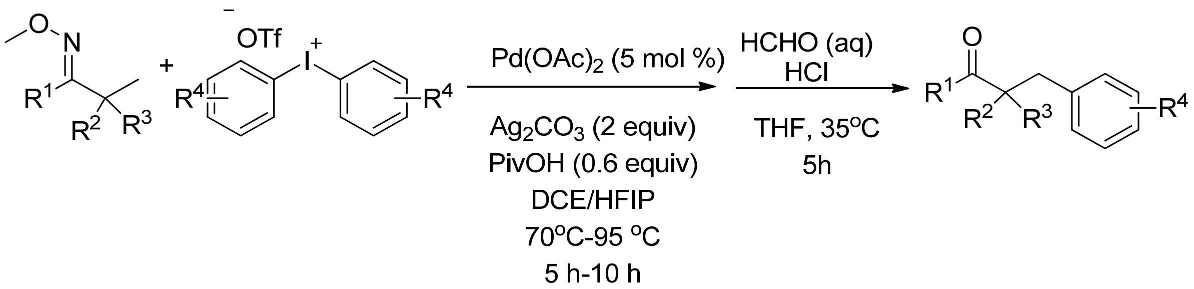


Diaryliodonium salts (0.25mmol), $Pd(OAc)_2$ (0.0125 mmol, 2.8 mg) and $Ag_2CO_3$ (0.5 mmol, 137.9mg) were placed in a tube. The tube was evacuated and recharged with $N_2$ for 3 times. Mixing solvent of DCE (1.5 ml) and HFIP (0.5 ml) was dropped in. Then appropriate ketoximes (0.25 mmol) and PivOH (0.15 mmol, 16.9 μl) was slowly added to the mixture. The tube was sealed and the mixture was allowed to stir at 70-90 °C for 5h. After completion, the mixture was filtered through celite and THF (2 ml) was added in the filtrate. Then 35% aqueous formaldehyde solution (1ml) as well as 10% aqueous HCl solution (0.5 ml) was added to the reaction mixture. Being stirred at 35°C for 5 h, the mixture was diluted with ethyl acetate (10 mL), neutralized with $NaHCO_3$ aq. and washed with water (10 mL). The organic layer was dried over anhydrous $MgSO_4$. Evaporation of the solvent followed by purification on silica gel (petroleum ether/ethyl acetate 100/1 to 50/1) provided the corresponding ketones.

## 3. Spectroscopic Data of Corresponding Products

***(E)*-2-benzylcyclohexanone *O*-methyl oxime (3aa):** colorless liquid; yield: 83%

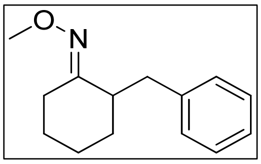


$^1$H NMR (301 MHz, CHLOROFORM-D) δ 7.31 – 7.23 (m, 2H), 7.18 (dd, *J* = 6.9, 4.5 Hz, 3H), 3.83 (s, 3H), 3.12 (dd, *J* = 13.4, 4.7 Hz, 1H), 2.82 – 2.70 (m, 1H), 2.59 (dd, *J* = 13.4, 9.8 Hz, 1H), 2.53 – 2.41 (m, 1H), 2.20 (m, 1H), 1.75 – 1.63 (m, 3H), 1.65 – 1.51 (m, 2H), 1.39 (m, 2H).$^{13}$C NMR (76 MHz, CHLOROFORM-D) δ 162.0, 140.9, 129.4 128.3, 126.0 61.22, 43.8, 37.3, 31.8, 26.4, 24.3, 23.8. IR (neat, $\nu_{C=N}$) 1736 cm$^{-1}$. EI-MS: 217 (M$^+$). HRMS (ESI, m/z) calcd for $C_{14}H_{19}NO$ [M+H]$^+$: 218.1539, found: 218.1535

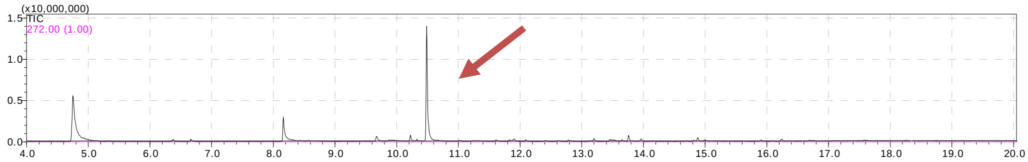


*GC-MS spectra of the extracted reaction mixture: red arrow indicated the peak of the product*

***(E)*-2-(4-bromobenzyl)cyclohexanone *O*-methyl oxime (3ad)**: colorless liquid; yield: 80%

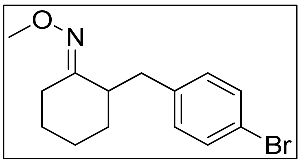


$^1$H NMR (400 MHz, CHLOROFORM-D) δ 7.39 (d, *J* = 8.3 Hz, 2H), 7.06 (d, *J* = 8.3 Hz, 2H), 3.08 (dd, *J* = 13.7, 5.3 Hz, 1H), 2.80 (dt, *J* = 13.7, 5.6 Hz, 1H), 2.54 (dd, *J* = 13.7, 9.6 Hz, 1H), 2.41 (dt, *J* = 8.5, 4.5 Hz, 1H), 2.13 (ddd, *J* = 14.0, 9.6, 4.5 Hz, 1H), 1.76 – 1.65 (m, 3H), 1.60 – 1.50 (m, 1H), 1.42 (dt, *J* = 9.0, 6.2 Hz, 1H), 1.33 – 1.22 (m, 1H).$^{13}$C NMR (101 MHz, CHLOROFORM-D) δ 161.4, 139.9, 131.3, 131.1, 119.7, 61.2, 43.7, 36.7, 32.0, 26.3, 24.4, 24.0IR (neat, $\nu_{C=N}$) 1736 cm$^{-1}$. EI-MS: 295 (M$^+$). HRMS (ESI, m/z) calcd for $C_{14}H_{18}{}^{79}BrNO$ [M+Na]$^+$: 296.0645, found: 296.0646

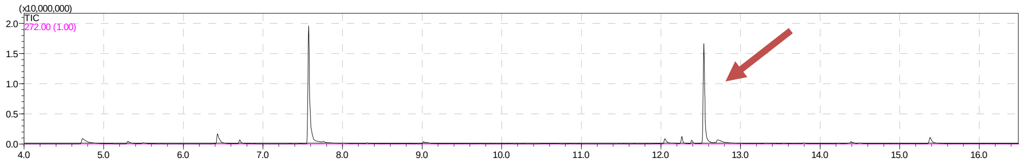


*GC-MS spectra of the extracted reaction mixture: red arrow indicated the peak of the product*

***(E)*-2-(4-(trifluoromethyl)benzyl)cyclohexanone *O*-methyl oxime (3ah):** colorless liquid; yield: 86%

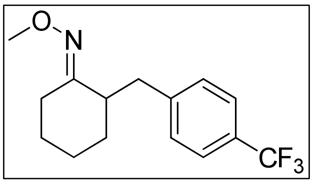


$^1$H NMR (400 MHz, CHLOROFORM-D) δ 7.52 (d, *J* = 8.0 Hz, 2H), 7.29 (d, *J* = 8.0 Hz, 2H), 3.19 (dd, *J* = 13.8, 5.3 Hz, 1H), 2.84 (dt, *J* = 13.8, 5.3 Hz, 1H), 2.64 (dd, *J* = 13.7, 9.0 Hz, 1H), 2.54 – 2.39 (m, 1H), 2.14-2.08 (m, 1H), 1.75 – 1.67 (m, 3H), 1.57 – 1.50 (m, 1H), 1.47 – 1.39 (m, 1H), 1.39 – 1.31 (m, 1H).$^{13}$C NMR (101 MHz,

CHLOROFORM-D) δ 161.2, 145.2, 129.7, 128.35 (d, *J* = 32.2 Hz), 125.2, 61.3, 43.7, 37.2, 32.3, 26.3, 24.5, 24.IR (neat, $\nu_{C=N}$) 1737 $cm^{-1}$. EI-MS: 285 ($M^+$). EI-MS: 295 ($M^+$). HRMS (ESI, m/z) calcd for $C_{15}H_{18}F_3NO$ $[M+Na]^+$: 286.1413, found: 286.1417.

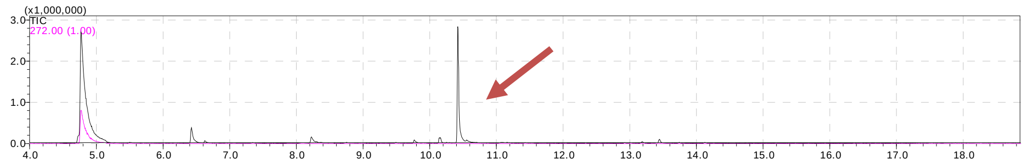


*GC-MS spectra of the extracted reaction mixture: red arrow indicated the peak of the product*

***(E)*-2-(2-fluorobenzyl)cyclohexanone *O*-methyl oxime (3aj)**: colorless liquid; yield: 78%

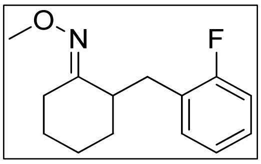

$^1H$ NMR (400 MHz, CHLOROFORM-D) δ 7.18 (m, 2H), 7.02 (m, 2H), 3.81 (s, 3H), 3.11 (dd, *J* = 13.7, 5.6 Hz, 1H), 2.86 – 2.76 (m, 1H), 2.70 (dd, *J* = 13.7, 9.2 Hz, 1H), 2.56 – 2.43 (m, 1H), 2.16 (m, 1H), 1.77 – 1.64 (m, 3H), 1.56 (dd, *J* = 8.8, 4.0 Hz, 1H), 1.42 (m, 2H).$^{13}C$ NMR (101 MHz, CHLOROFORM-D) δ 161.5 (d, *J* = 244.8 Hz), 161.5, 131.8 (d, *J* = 5.0 Hz), 127.7 (d, *J* = 8.2 Hz), 123.9 (s), 115.2 (d, *J* = 22.4 Hz), 61.2, 42.7, 32.2, 30.6, 24.3, 24.0. IR (neat, $\nu_{C=N}$) 1737 $cm^{-1}$. EI-MS: 235 ($M^+$). HRMS (ESI, m/z) calcd for $C_{15}H_{18}FNO$ $[M+Na]^+$:236.1445 , found: 236.1442.

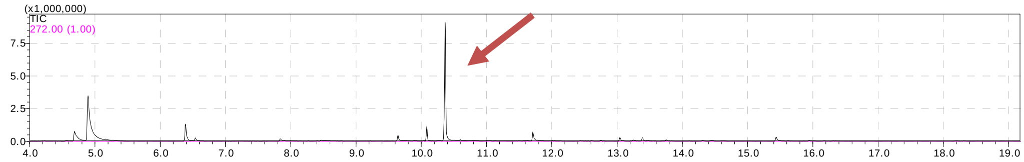


*GC-MS spectra of the extracted reaction mixture: red arrow indicated the peak of the product*

***(E)*-2-(2-chlorobenzyl)cyclohexanone *O*-methyl oxime (3ak)** :colorless liquid; yield: 72%

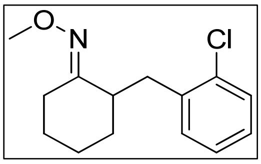

$^1H$ NMR (400 MHz, CHLOROFORM-D) δ 7.25 (d, *J* = 7.5 Hz, 1H), 7.16 (dd, *J* = 9.2, 4.2 Hz, 1H), 7.06 (dt, *J* = 7.3, 4.2 Hz, 2H), 3.74 (s, 3H), 3.17 (dd, *J* = 13.7, 5.5 Hz, 1H), 2.80 (dt, *J* = 13.8, 5.1 Hz, 1H), 2.68 (dd, *J* = 13.7, 8.9 Hz, 1H), 2.55 – 2.42 (m, 1H), 2.07 – 1.94 (m, 1H), 1.74 – 1.58 (m, 3H), 1.52 – 1.41 (m, 1H), 1.40 – 1.25 (m, 2H). $^{13}C$ NMR (101 MHz, CHLOROFORM-D) δ 161.4, 138.6, 134.5 131.9, 129.6, 127.5, 126.5, 61.2, 42.2, 34.9, 32.5, 26.4, 24.5, 24.4. IR (neat, $\nu_{C=N}$) 1737 $cm^{-1}$, EI-MS: 251 ($M^+$). HRMS (ESI, m/z) calcd for $C_{15}H_{18}{}^{35}ClNO$ $[M+H]^+$: 252.1150 , found: 252.1152.

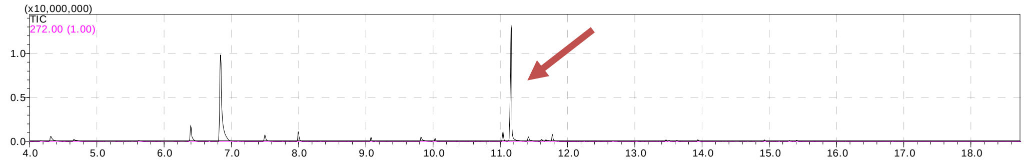


*GC-MS spectra of the extracted reaction mixture: red arrow indicated the peak of the product*

***(E)*-methyl 4-((2-(methoxyimino)cyclohexyl)methyl)benzoate (3ag):** colorless solid; yield: 87%; m.p.67-69°C

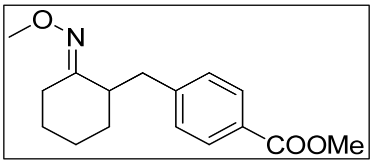


$^1$H NMR (301 MHz, CHLOROFORM-D) δ 7.95 (m, 2H), 7.25 (m, 2H), 3.90 (s, 3H), 3.81 (s, 3H), 3.19 (dd, *J* = 13.6, 5.3 Hz, 1H), 2.83 (m, 1H), 2.63 (dd, *J* = 13.6, 9.2 Hz, 1H), 2.47 (tt, *J* = 8.8, 4.4 Hz, 1H), 2.12 (m, 1H), 1.70 (m, 3H), 1.58 – 1.48 (m, 1H), 1.48 – 1.24 (m, 2H).$^{13}$C NMR (76 MHz, CHLOROFORM-D) δ 167.2, 161.2, 146.6, 129.6, 129.4, 128.0, 61.2, 52.1, 43.6, 37.4, 32.2, 26.3, 24.4, 24.0. IR (neat, $\nu_{C=O}$) 1722 cm$^{-1}$(The peak of $\nu_{C=N}$ was overlapped). EI-MS: 275 (M$^+$). HRMS (ESI, m/z) calcd for $C_{16}H_{21}NO_3$ [M+H]$^+$: 276.1594 , found: 276.1591. $[\alpha]_D^{23}$+49.2° (1, DCM)

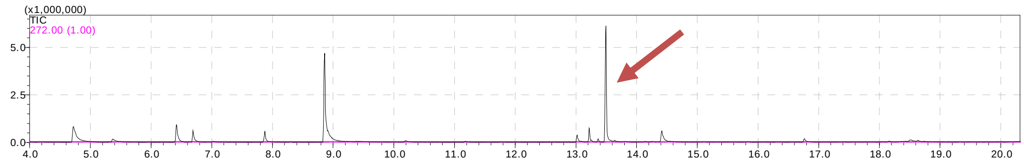


*GC-MS spectra of the extracted reaction mixture: red arrow indicated the peak of the product*

***(E)*-2-(4-methoxybenzyl)cyclohexanone *O*-methyl oxime (3ai):** colorless liquid yield: 80%

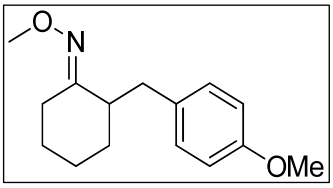


$^1$H NMR (400 MHz, CHLOROFORM-D) δ 7.08 (m, 2H), 6.81 (m, 2H), 3.81 (s, 3H), 3.77 (s, 3H), 3.03 (dd, *J* = 13.7, 5.0 Hz, 1H), 2.78 – 2.67 (m, 1H), 2.53 (dd, *J* = 13.7, 9.7 Hz, 1H), 2.42 (dt, *J* = 12.9, 4.7 Hz, 1H), 2.22 (m, 1H), 1.68 (mz, 3H), 1.57 (m, 1H), 1.44 – 1.34 (m, 2H).$^{13}$C NMR (101 MHz, CHLOROFORM-D) δ 162.1, 158.0, 132.8, 130.3, 113.7, 61.2, 55.4, 43.9, 36.4, 31.6, 26.4, 24.2, 23.7. IR (neat, $\nu_{C=N}$) 1737 cm$^{-1}$, EI-MS: 251 (M$^+$). HRMS (ESI, m/z) calcd for $C_{15}H_{21}NO_2$ [M+H]$^+$: 248.1645 , found: 248.1645.

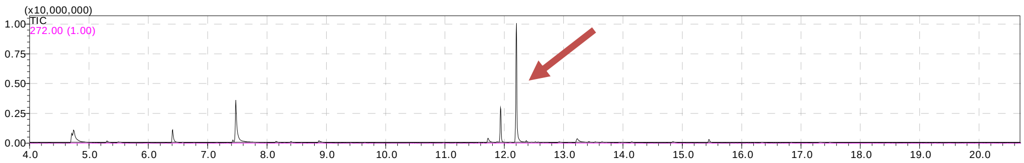


*GC-MS spectra of the extracted reaction mixture: red arrow indicated the peak of the product*

**(*R,E*)-methyl4-((4-isopropyl-6-(methoxyimino)cyclohex-1-en-1-yl)methyl)benzoate (3jg):** colorless liquid; yield: 38%

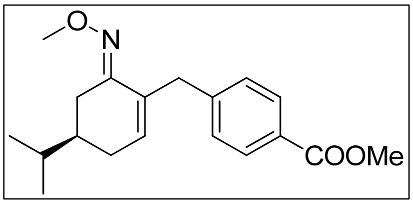


$^1$H NMR (400 MHz, CHLOROFORM-D) δ 7.93 (m, 2H), 7.29 (m, 2H), 5.88 (m, 1H), 3.90 (s, 3H), 3.86 (s, 3H), 3.61 (m, 2H), 3.06 (dd, *J* = 16.5, 2.5 Hz, 1H), 2.27 – 2.12 (m, 1H), 1.87 (m, 1H), 1.77 (dd, *J* = 16.5, 12.7 Hz, 1H), 1.56 – 1.43 (m, 2H), 0.89 (m, 6H).$^{13}$C NMR (101 MHz, CHLOROFORM-D) δ 167.4, 155.2, 146.6, 134.2, 133.8, 129.5, 129.4, 127.8, 61.9, 52.1, 39.4, 37.2, 32.2, 28.9, 26.7, 19.9, 19.8. IR (neat, $\nu_{C=O}$) 1723 cm$^{-1}$(The peak of $\nu_{C=N}$ was overlapped). EI-MS: 315 (M$^+$). HRMS (ESI, m/z)

calcd for $C_{19}H_{25}NO_3$ $[M+H]^+$: 316.1907 , found: 316.1911. $[\alpha]_D^{23}$-15.8° (1, DCM)

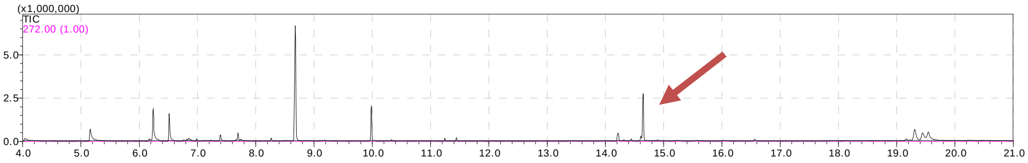


*GC-MS spectra of the extracted reaction mixture: red arrow indicated the peak of the product*

**Methyl 4-((*1S,2R,3S,5R,7S*)-1-((*E*)-1-(methoxyimino)ethyl)adamantan-2-yl)benzoate (3hg):** colorless liquid; yield: 47%

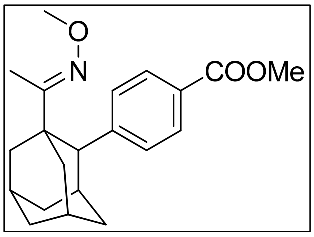


$^1$H NMR (400 MHz, CHLOROFORM-D) δ 7.90 (d, $J$ = 8.3 Hz, 2H), 7.45 (d, $J$ = 8.3 Hz, 2H), 3.89 (s, 3H), 3.77 (s, 3H), 3.24 (m, 1H), 2.58 (d, $J$ = 13.6 Hz, 1H), 2.11 (dd, $J$ = 6.0, 3.0 Hz, 3H), 2.05 – 1.94 (m, 4H), 1.75 (s, 2H), 1.67 (s, 1H), 1.59 (d, $J$ = 9.0 Hz, 1H), 1.54 (s, 3H), 1.48 (d, $J$ = 13.1 Hz, 1H). $^{13}$C NMR (101 MHz, CHLOROFORM-D) δ 167.3, 162.2, 150.0, 129.3, 129.0, 127.5, 61.3, 52.1, 51.5, 44.3, 42.0, 39.7, 37.5, 36.4, 35.1, 30.5, 28.5, 27.9, 9.5. IR (neat, $\nu_{C=O}$) 1723 cm$^{-1}$(The peak of $\nu_{C=N}$ was overlapped). EI-MS: 342 ($M^+$). HRMS (ESI, m/z) calcd for $C_{19}H_{25}NO_3$ $[M+H]^+$: 342.2064 , found: 342.2069

***(E)*-methyl 4-((3-(methoxyimino)-2,4-dimethylbicyclo[2.2.1]heptan-2-yl)methyl)benzoate**

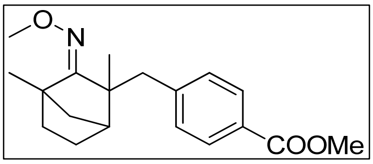


**(3ig):** colorless solid; yield: 65%; m.p.74-76°C

$^1$H NMR (400 MHz, CHLOROFORM-D) δ 7.94 (d, $J$ = 8.0 Hz, 2H), 7.27 (d, $J$ = 8.0 Hz, 2H), 3.90 (s, 3H), 3.80 (s, 3H), 3.10 (d, $J$ = 13.6 Hz, 1H), 2.91 (d, $J$ = 13.6 Hz, 1H), 1.75 (m, 2H), 1.71 – 1.41 (m, 3H), 1.43 – 1.31 (m, 2H), 1.26 (s, 3H), 1.17 (s, 3H).$^{13}$C NMR (101 MHz, CHLOROFORM-D) δ 171.6, 167.4 145.5, 130.6, 129.2, 127.9, 61.4, 54.3, 52.1, 47.9, 45.0, 40.1, 37.3, 31.4, 24.8, 23.2, 22.7. IR (neat, $\nu_{C=O}$) 1723 cm$^{-1}$(The peak of $\nu_{C=N}$ was overlapped), EI-MS: 315 ($M^+$). HRMS (ESI, m/z) calcd for $C_{19}H_{25}NO_3$ $[M+H]^+$: 316.1907 , found: 316.1911. $[\alpha]_D^{23}$+13.7° (1, DCM)

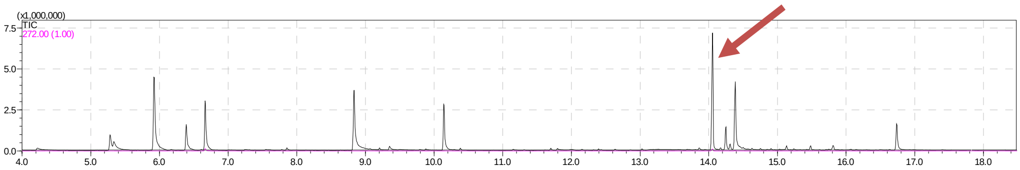


*GC-MS spectra of the extracted reaction mixture: red arrow indicated the peak of the product*

**dimethyl 4,4'-(((*6aR,6bS,8aS,11S,12aS,14aR,14bS,E*)-11-(methoxycarbonyl)-3-(methoxyimino)-6a,6b,8a,11,14b-pentamethyl-14-oxo-1,2,3,4,4a,5,6,6a,6b,7,8,8a,9,10,11,12,12a,14,14a,14b-icosahydropicene-4,4-diyl)bis(methylene))dibenzoate (3lg)**: colorless solid; yield: 73%; m.p.277°C-279°C

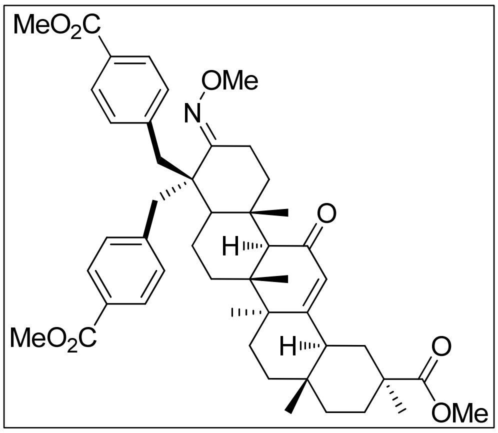


$^1$H NMR (400 MHz, CHLOROFORM-D) δ 7.92 (d, *J* = 8.2 Hz, 2H), 7.86 (d, *J* = 8.2 Hz, 2H), 7.33 (d, *J* = 8.3 Hz, 2H), 7.09 (d, *J* = 8.1 Hz, 2H), 5.61 (s, 1H), 3.90 (s, 3H), 3.88 (s, 3H), 3.70 (s, 3H), 3.67 (s, 3H), 3.38 (d, *J* = 13.7 Hz, 1H), 3.17 (t, *J* = 14.5 Hz, 2H), 2.78 (t, *J* = 14.6 Hz, 2H), 2.54 (d, *J* = 13.9 Hz, 1H), 2.17 (dd, *J* = 14.5, 4.9 Hz, 1H), 2.06 (m, 1H), 2.00 (d, *J* = 14.5 Hz, 2H), 1.83 (m, 2H), 1.76 (m, 2H), 1.53 (m, 2H), 1.42 (s, 3H), 1.38 (m, 4H), 1.30 (s, 1H), 1.27 (m, 2H), 1.15 (s, 3H), 1.11 (s, 3H), 1.02 (s, 3H), 0.86 (m 2H), 0.78 (s, 3H). $^{13}$C NMR (101 MHz, CHLOROFORM-D) δ 199.7, 177.0, 169.5 167.4, 167.3, 158.1, 146.0, 143.2, 131.5, 131.3, 129.0, 128.7, 128.3, 128.3, 127.9, 61.7, 61.5, 52.1, 52.1, 51.9, 49.5, 49.2, 48.6, 45.4, 44.1, 43.3, 42.1, 41.1, 39.2, 37.8, 37.3, 36.7, 32.0, 31.6, 31.3, 28.7, 28.4, 27.1, 26.4, 23.0, 19.2, 18.5, 18.4, 16.0. IR (neat, $\nu_{C=O}$) 1724 cm$^{-1}$(The peak of $\nu_{C=N}$ was overlapped). HRMS (ESI, m/z) calcd for $C_{48}H_{61}NO_8$ [M+H]$^+$: 780.4470 , found: 780.4474. $[\alpha]_D^{23}$+54.8° (1, DCM)

**dimethyl4,4'-(((*10S,13R,14R,17R,E*)-3-(methoxyimino)-10,13,14-trimethyl-17-((*R*)-6-methylheptan-2-yl)-2,3,4,5,6,7,10,11,12,13,14,15,16,17-tetradecahydro-1H-cyclopenta[a]phenanthrene-4,4-diyl)bis(methylene))dibenzoate:** colorless solid; yield: 73%; m.p.143-145°C

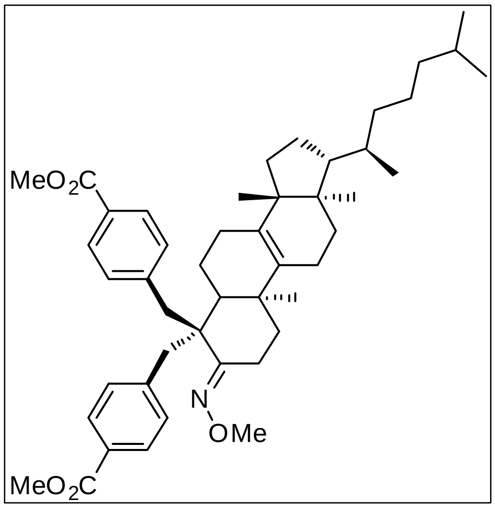


$^1$H NMR (400 MHz, CHLOROFORM-D) δ 7.93 (d, *J* = 8.2 Hz, 2H), 7.83 (d, *J* = 8.3 Hz, 2H), 7.30 (d, *J* = 8.3 Hz, 2H), 7.10 (d, *J* = 8.2 Hz, 2H), 3.91 (s, 3H), 3.87 (s, 3H), 3.71 (s, 3H), 3.30 (d, *J* = 13.5 Hz, 1H), 3.18 (d, *J* = 13.5 Hz, 1H), 2.77 (d, *J* = 13.3 Hz, 1H), 2.59 (d, *J* = 13.6 Hz, 1H), 2.15 – 1.73 (m, 10H), 1.65 (m, 2H), 1.51 (m, 2H), 1.31 (m, 8H), 1.21 (s, 3H), 1.12 (m, 3H), 1.02 – 0.91 (m, 2H), 0.86 (m, 9H), 0.67 (d, *J* = 4.0 Hz, 6H). $^{13}$C NMR (101 MHz, CHLOROFORM-D) δ 167.4, 158.2, 145.6, 143.7, 135.0, 134.1 131.5, 131.5, 128.9 128.7, 128.1, 127.7, 61.5, 52.1, 52.1, 50.6, 49.9, 49.2, 45.1, 44.5, 42.0, 39.6, 39.1, 36.9, 36.6, 34.2, 31.0, 30.9, 29.9, 28.3, 28.1, 25.8, 24.2, 24.3, 24.1, 24.1, 23.0, 22.7, 21.1, 19.4, 19.3, 19.1, 18.8, 16.0. IR (neat, $\nu_{C=N}$, $\nu_{C=O}$,$\nu_{C=C}$) 1737 ,1726,1609 cm$^{-1}$. HRMS (ESI, m/z) calcd for $C_{47}H_{65}NO_5$ [M+Na]$^+$: 746.4755 , found: 746.4753. $[\alpha]_D^{23}$+20.5° (1, DCM)

**X-ray crystal structure analysis of compound 3kg:** Single crystals suitable for X-ray analysis were obtained by slow evaporation of its solution in hexane. The crystal structure has been deposited at the Cambridge Crystallographic Data Centre and allocated the deposition number: **CCDC 1057827.** Formula: $C_{47}H_{65}NO_5$, $M$ = 724.00, colourless crystal, 0.28 x 0.15 x 0.05 mm, $a$ = 35.188(7), $b$ = 7.4837(15), $c$ = 16.526(3) Å, α = 90.00, β =98.87(3), γ = 90.00 , $V$ = 4299.8(15) Å$^3$, $\rho_{calc}$ = 1.118 gcm$^{-3}$, μ = 0.071 mm$^{-1}$, $Z$ = 4, Monoclinic, space group C2, λ = 0.71073 Å, $T$ = 173(2) K. Data completeness = 0.998, Theta (max) = 27.48

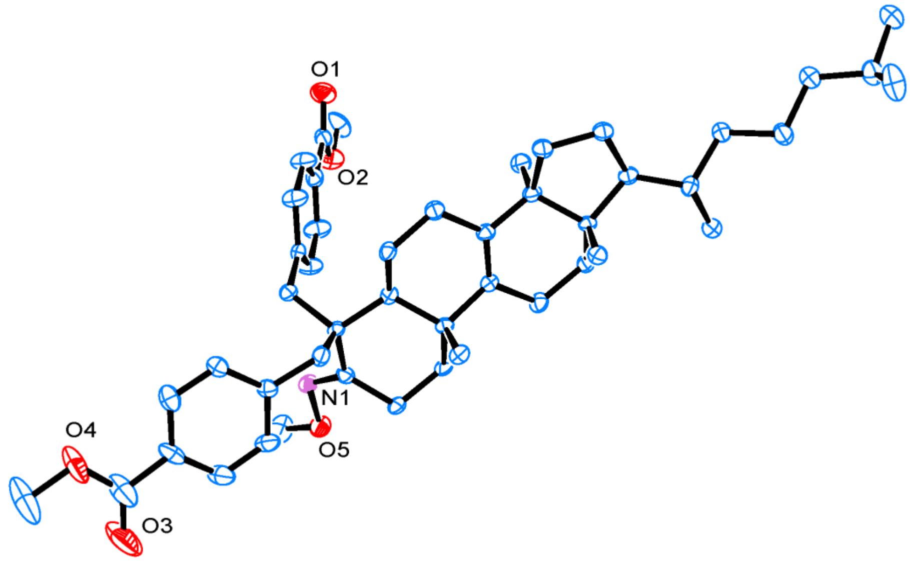

**2-benzylcyclohexanone (4aa)[8]:** colorless liquid; yield: 80%

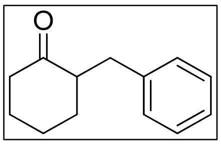

$^{1}$H NMR (301 MHz, CHLOROFORM-D) δ 7.27 (dd, *J* = 9.4, 4.8 Hz, 2H), 7.21 – 7.10 (m, 3H), 3.24 (dd, *J* = 13.7, 4.6 Hz, 1H), 2.62 – 2.49 (m, 1H), 2.42 (dt, *J* = 13.4, 6.4 Hz, 2H), 2.31 (dd, *J* = 13.4, 5.8 Hz, 1H), 2.13 – 1.98 (m, 2H), 1.90 – 1.78 (m, 1H), 1.72 – 1.54 (m, 2H), 1.35 (ddd, *J* = 24.8, 12.4, 3.5 Hz, 1H).$^{13}$C NMR (76 MHz, CHLOROFORM-D) δ 212.7, 140.5, 129.3, 128.4 126.1, 52.6, 42.3, 35.6, 33.6, 28.2, 25.2. IR (neat, $\nu_{C=O}$) 1706cm$^{-1}$. EI-MS: 188 (M$^{+}$). HRMS (ESI, m/z) calcd for $C_{13}H_{16}O$ [M+Na]$^{+}$: 211.1093 , found: 211.1093.

**2-(4-fluorobenzyl)cyclohexanone (4ab)[8]** :colorless liquid; yield: 80%.

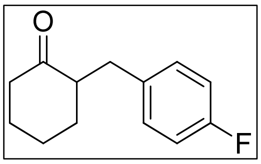

$^{1}$H NMR (400 MHz, CHLOROFORM-D) δ 7.18 – 7.04 (m, 2H), 6.94 (m, 2H), 3.17 (dd, *J* = 13.9, 4.6 Hz, 1H), 2.58 – 2.45 (m, 1H), 2.48 – 2.28 (m, 3H), 2.12 – 1.97 (m, 2H), 1.84 (dd, *J* = 10.6, 2.4 Hz, 1H), 1.73 – 1.53 (m, 2H), 1.39 – 1.28 (m, 1H).

$^{13}$C NMR (101 MHz, CHLOROFORM-D) δ 212.5, 161.4 (d, *J* = 243.7 Hz), 136.0, 130.6 (d, *J* = 7.8 Hz), 115.1 (d, *J* = 21.1 Hz), 52.64 (s), 42.3, 34.8, 33.6, 28.1, 25.2. IR (neat, $\nu_{C=O}$) 1711cm$^{-1}$, EI-MS: 206 (M$^{+}$). EI-MS: 188 (M$^{+}$). HRMS (ESI, m/z) calcd for $C_{13}H_{15}FO$ [M+Na]$^{+}$: 229.0999 , found: 229.0997.

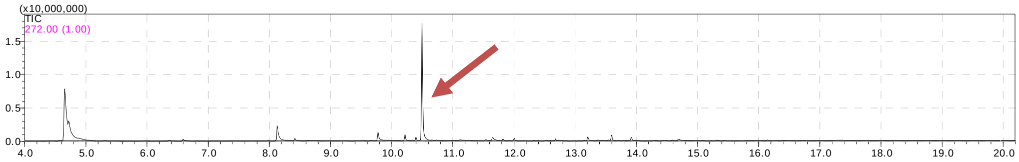


*GC-MS spectra of the extracted reaction mixture: red arrow indicated the peak of the aimed product in form of oxime without hydrolysis*

**2-(4-chlorobenzyl)cyclohexanone (4ac)[9]:** colorless liquid; yield: 78%

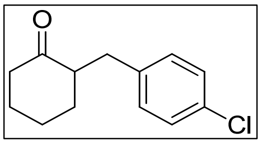

$^{1}$H NMR (400 MHz, CHLOROFORM-D) δ 7.23 (d, *J* = 8.4 Hz, 2H), 7.09 (d, *J* = 8.3 Hz, 2H), 3.17 (dd, *J* = 13.9, 5.1 Hz, 1H), 2.60 – 2.46 (m, 1H), 2.47 – 2.36 (m, 2H), 2.34 – 2.24 (m, 1H), 2.04 (dddd, *J* = 21.8, 12.9, 5.8, 2.7 Hz, 2H), 1.84 (ddd, *J* = 11.6, 5.6, 3.3 Hz, 1H), 1.74 – 1.53 (m, 2H), 1.34 (ddd, *J* = 25.0, 12.3, 3.6 Hz, 1H).$^{13}$C NMR (101 MHz, CHLOROFORM-D) δ 212.3, 139.0, 131.8, 130.6, 128.5, 52.5, 42.3 35.0, 33.61, 28.1, 25.2. IR (neat, $\nu_{C=O}$) 1709cm$^{-1}$, EI-MS: 226 (M$^{+}$). HRMS (ESI, m/z) calcd for $C_{14}H_{15}{}^{35}ClO$ [M+Na]$^{+}$: 245.0704 , found: 245.0708.

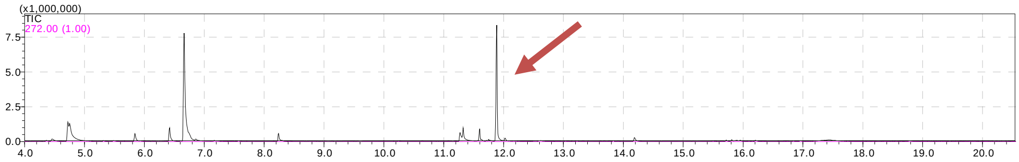


*GC-MS spectra of the extracted reaction mixture: red arrow indicated the peak of the aimed product in form of oxime without hydrolysis*

**2-(4-bromobenzyl)cyclohexanone (4ad)**[10] :colorless liquid; yield: 78%

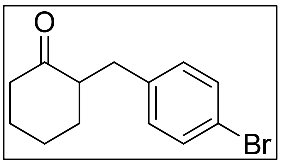


$^{1}$H NMR (400 MHz, CHLOROFORM-D) δ 7.38 (d, *J* = 8.3 Hz, 2H), 7.03 (d, *J* = 8.3 Hz, 2H), 3.16 (dd, *J* = 13.9, 5.1 Hz, 1H), 2.56 – 2.47 (m, 1H), 2.45 – 2.26 (m, 3H), 2.04 (dddd, *J* = 12.8, 8.2, 5.9, 2.7 Hz, 2H), 1.88 – 1.80 (m, 1H), 1.70 – 1.56 (m, 2H), 1.40 – 1.29 (m, 1H). $^{13}$C NMR (101 MHz, CHLOROFORM-D) δ 212.3, 139.5, 131.5, 131.1, 120.0, 52.5, 42.3, 35.1, 33.6, 28.2, 25.3. IR (neat, $\nu_{C=O}$) 1708cm$^{-1}$. EI-MS: 266 (M$^{+}$). HRMS (ESI, m/z) calcd for $C_{13}H_{15}{}^{79}BrO$ [M+Na]$^{+}$: 289.0198 , found: 289.0199.

**2-(4-(trifluoromethyl)benzyl)cyclohexanon (4ah)**: colorless liquid; yield: 84%

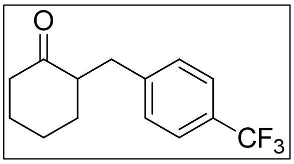


$^{1}$H NMR (400 MHz, CHLOROFORM-D) δ 7.52 (d, *J* = 7.9 Hz, 2H), 7.27 (d, *J* = 9.6 Hz, 2H), 3.26 (dd, *J* = 13.6, 4.8 Hz, 1H), 2.63 – 2.53 (m, 1H), 2.53 – 2.42 (m, 2H), 2.33 (td, *J* = 13.0, 5.9 Hz, 1H), 2.05 (dd, *J* = 26.0, 12.4 Hz, 2H), 1.85 (d, *J* = 12.0 Hz, 1H), 1.65 (dt, *J* = 21.7, 12.6 Hz, 2H), 1.38 (dt, *J* = 21.6, 7.8 Hz, 1H).$^{13}$C NMR (101 MHz, CHLOROFORM-D) δ 212.02 (s), 144.8, 129.6, 128.5 (d, *J* = 32.3 Hz), 125.3 (d, *J* = 3.7 Hz), 124.5 (d, *J* = 271.9 Hz), 52.3, 42.3, 35.5, 33.8, 28.1, 25.3. IR (neat, $\nu_{C=O}$) 1711cm$^{-1}$, EI-MS: 256 (M$^{+}$). HRMS (ESI, m/z) calcd for $C_{14}H_{15}F_{3}O$ [M-H]$^{-}$: 255.1002 , found: 255.1005.

**2-(4-methylbenzyl)cyclohexanone (4ae)**: colorless liquid; yield: 77%

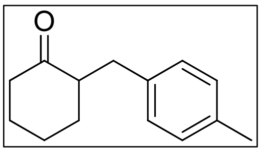


$^{1}$H NMR (400 MHz, CHLOROFORM-D) δ 7.05 (m, 4H), 3.18 (dd, *J* = 13.9, 4.7 Hz, 1H), 2.56 – 2.47 (m, 1H), 2.46 – 2.31 (m, 3H), 2.28 (m, 3H), 2.09 – 1.96 (m, 2H), 1.81 (dd, *J* = 12.9, 3.6 Hz, 1H), 1.71 – 1.52 (m, 2H), 1.40 – 1.29 (m, 1H).$^{13}$C NMR (101 MHz, CHLOROFORM-D) δ 212.9, 137.4, 135.6, 129.2, 129.1, 52.7, 42.3, 35.1, 33.5, 28.2, 25.2, 21.1IR (neat, $\nu_{C=O}$) 1711cm$^{-1}$, EI-MS: 202 (M$^{+}$). HRMS (ESI, m/z) calcd for $C_{14}H_{18}O$ [M+Na]$^{+}$: 225.1250 , found: 225.1245.

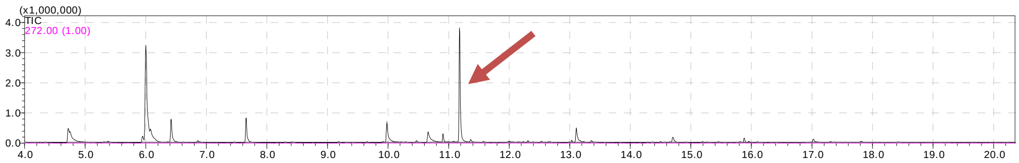


*GC-MS spectra of the extracted reaction mixture: red arrow indicated the peak of the aimed product in form of oxime without hydrolysis.*

**2-(2-chlorobenzyl)cyclohexanone (4ak):** colorless liquid; yield: 69%

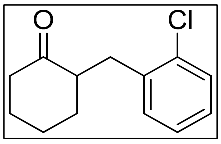


$^{1}$H NMR (301 MHz, CHLOROFORM-D) δ 7.33 (dd, *J* = 7.2, 2.1 Hz, 1H), 7.25 – 7.20 (m, 1H), 7.20 – 7.08 (m, 2H), 3.33 (dd, *J* = 13.6, 5.0 Hz, 1H), 2.75 – 2.63 (m, 1H), 2.64 – 2.51 (m, 1H), 2.47 – 2.28 (m, 2H), 2.10 – 1.94 (m, 2H), 1.90 – 1.80 (m, 1H), 1.75 – 1.59 (m, 2H), 1.41 (m, 1H).$^{13}$C NMR (76 MHz, CHLOROFORM-D) δ 212.4, 138.2 134.3, 132.0, 129.6, 127.7, 126.7, 50.7, 42.4, 33.8, 33.5, 28.3, 25.4 IR (neat, $\nu_{C=O}$) 1709cm$^{-1}$, EI-MS: 222 (M$^{+}$). HRMS (ESI, m/z) calcd for $C_{14}H_{15}{}^{35}ClO$ [M+Na]$^{+}$: 245.0704 , found: 245.0707.

**2-(2-fluorobenzyl)cyclohexanone (4aj):** colorless liquid; yield: 74%

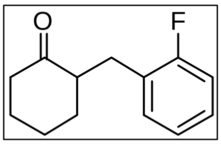

$^{1}$H NMR (301 MHz, CHLOROFORM-D) δ 7.22 – 7.08 (m, 2H), 7.14 – 6.93 (m, 2H), 3.20 (dd, *J* = 13.2, 4.1 Hz, 1H), 2.68 – 2.30 (m, 4H), 2.11 – 1.96 (m, 2H), 1.93 – 1.78 (m, 1H), 1.77 – 1.58 (m, 2H), 1.49 – 1.34 (m, 1H).$^{13}$C NMR (76 MHz, CHLOROFORM-D) δ 212.5 (s), 161.4 (d, *J* = 244.5 Hz), 131.9 (d, *J* = 5.0 Hz), 127.9 (d, *J* = 8.2 Hz), 127.3 (d, *J* = 15.8 Hz), 124.0, 115.3 (d, *J* = 22.2 Hz), 51.2, 42.3, 33.6 29.1 28.2, 25.2. IR (neat, $\nu_{C=O}$) 1709cm$^{-1}$, EI-MS: 206 (M$^{+}$). HRMS (ESI, m/z) calcd for $C_{14}H_{15}FO$ [M+Na]$^{+}$: 229.0999 , found: 229.0996.

**2-(2-bromobenzyl)cyclohexanone (4al)**[9] **:** colorless liquid; yield: 56%

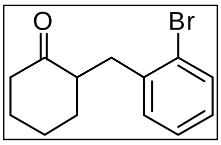

$^{1}$H NMR (400 MHz, CHLOROFORM-D) δ 7.45 (d, *J* = 8.0 Hz, 1H), 7.22 – 7.09 (m, 2H), 6.98 M, 1H), 3.28 (dd, *J* = 13.8, 5.2 Hz, 1H), 2.68 – 2.59 (m, 1H), 2.49 (dd, *J* = 13.8, 8.1 Hz, 1H), 2.36 (dd, *J* = 13.6, 3.5 Hz, 1H), 2.27 (td, *J* = 12.9, 5.8 Hz, 1H), 2.07 – 1.91 (m, 2H), 1.83 – 1.71 (m, 1H), 1.68 – 1.51 (m, 2H), 1.45 – 1.31 (m, 1H).$^{13}$C NMR (101 MHz, CHLOROFORM-D) δ 212.3, 139.9, 132.9, 132.0, 127.9, 127.3, 124.9, 50.7 42.4, 35.9, 33.9, 28.3, 25.4. IR (neat, $\nu_{C=O}$) 1711cm$^{-1}$, EI-MS: 266 (M$^{+}$). HRMS (ESI, m/z) calcd for $C_{14}H_{15}{}^{79}BrO$ [M+Na]$^{+}$: 289.0198 , found: 289.0194.

**methyl 4-((2-oxocyclohexyl)methyl)benzoate (4ag)**: colorless liquid; yield: 81%

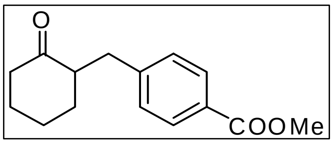

$^{1}$H NMR (301 MHz, CHLOROFORM-D) δ 7.94 (d, *J* = 8.2 Hz, 2H), 7.23 (d, *J* = 8.1 Hz, 2H), 3.90 (s, 3H), 3.27 (dd, *J* = 13.4, 4.5 Hz, 1H), 2.95 – 2.68 (m, 1H), 2.58 – 2.32 (m, 3H), 2.14 – 1.95 (m, 2H), 1.84 (d, *J* = 9.7 Hz, 1H), 1.73 – 1.51 (m, 2H), 1.46 – 1.31 (m, 1H).$^{13}$C NMR (76 MHz, CHLOROFORM-D) δ 212.1, 167.2, 146.2, 129.8, 129.3, 128.1, 52.3, 52.2, 42.3, 35.7, 33.7, 28.1, 25.3. IR (neat, $\nu_{C=O}$) 1709, 1722 cm$^{-1}$, EI-MS: 246 (M$^{+}$).HRMS (ESI, m/z) calcd for $C_{15}H_{18}O_3$ [M+Na]$^{+}$: 269.1148 , found: 269.1150.

**2-(4-methoxybenzyl)cyclohexanone (4ai)** : colorless liquid; yield: 76%

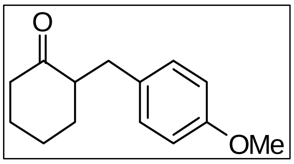

$^{1}$H NMR (400 MHz, CHLOROFORM-D) δ 7.07 (d, *J* = 8.3 Hz, 2H), 6.82 (d, *J* = 8.3 Hz, 2H), 3.78 (s, 3H), 3.16 (dd, *J* = 13.9, 4.3 Hz, 1H), 2.38 (tdd, *J* = 19.3, 18.9, 5.4 Hz, 4H), 2.04 (s, 2H), 1.83 (d, *J* = 13.1 Hz, 1H), 1.75 – 1.56 (m, 2H), 1.41 – 1.32 (m, 1H).$^{13}$C NMR (101 MHz, CHLOROFORM-D) δ 212.9, 158.0, 132.5, 130.2, 113.8, 55.4, 52.8, 42.3, 34.7, 33.5, 28.2, 25.2. IR (neat, $\nu_{C=O}$) 1709, 1722 cm$^{-1}$, EI-MS: 218 (M$^{+}$). HRMS (ESI, m/z) calcd for $C_{14}H_{18}O_2$ [M+H]$^{+}$: 241.1199 , found: 241.1193.

**2-(4-(tert-butyl)benzyl)cyclohexanone (4af)** :colorless liquid; yield: 67%

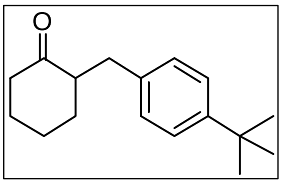

$^{1}$H NMR (400 MHz, CHLOROFORM-D) δ 7.29 (d, *J* = 8.2 Hz, 3H), 7.28 (s, 2H), 7.08 (d, *J* = 8.1 Hz, 2H), 7.08 (d, *J* = 8.1 Hz, 2H), 3.20 (dd, *J* = 14.0, 4.7 Hz, 1H), 3.20 (dd, *J* = 14.0, 4.7 Hz, 1H), 2.53 (dd, *J* = 7.7, 4.4

Hz, 1H), 2.53 (dd, $J$ = 7.7, 4.4 Hz, 1H), 2.38 (m, 3H), 2.38 (m, 3H), 2.21 – 1.94 (m, 2H), 2.13 – 2.01 (m, 2H), 1.94 – 1.75 (m, 1H), 1.87 – 1.77 (m, 1H), 1.75 – 1.53 (m, 2H), 1.73 – 1.55 (m, 2H), 1.43 – 1.34 (m, 2H), 1.30 (s, 9H). $^{13}$C NMR (101 MHz, CHLOROFORM-D) δ 212.9, 148.9, 137.4, 128.9, 125.3, 52.6, 42.3, 35.0, 34.5, 33.6, 31.5, 28.2, 25.2. IR (neat, $\nu_{C=O}$) 1709cm$^{-1}$, EI-MS: 244 (M$^+$). HRMS (ESI, m/z) calcd for $C_{17}H_{24}O$ [M+H]$^+$: 245.1900 , found: 245.1900.

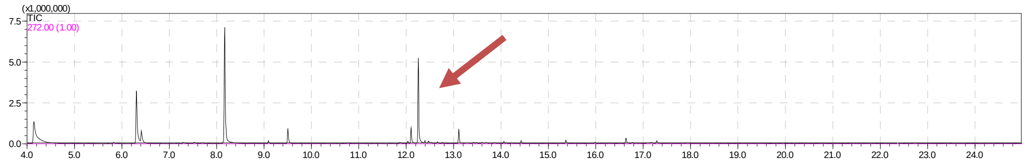

*GC-MS spectra of the extracted reaction mixture: red arrow indicated the peak of the aimed product in form of oxime without hydrolysis.*

**4-(4-bromophenyl)-3-methylbutan-2-one (4bd)**: colorless liquid; yield: 80%

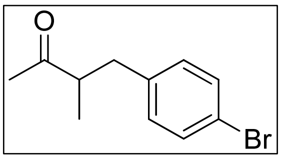

$^1$H NMR (400 MHz, CHLOROFORM-D) δ 7.38 (d, $J$ = 8.3 Hz, 2H), 7.01 (d, $J$ = 8.3 Hz, 2H), 2.94 (dd, $J$ = 13.6, 7.0 Hz, 1H), 2.78 (dd, $J$ = 14.2, 7.1 Hz, 1H), 2.50 (dd, $J$ = 13.6, 7.4 Hz, 1H), 2.08 (s, 3H), 1.07 (d, $J$ = 7.0 Hz, 3H).$^{13}$C NMR (101 MHz, CHLOROFORM-D) δ 211.8, 138.8, 131.6, 130.8, 120.2, 48.8, 38.3 29.0, 16.5. IR (neat, $\nu_{C=O}$) 1712cm$^{-1}$, EI-MS: 240 (M$^+$). HRMS (ESI, m/z) calcd for $C_{11}H_{13}{}^{79}BrO$ [M+Na]$^+$: 263.0072 , found: 263.0074.

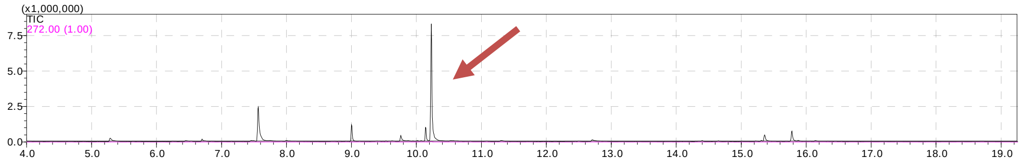

*GC-MS spectra of the extracted reaction mixture: red arrow indicated the peak of the aimed product in form of oxime without hydrolysis*

**1-(4-bromophenyl)pentan-3-one(4cd)**: colorless liquid; yield: 75%

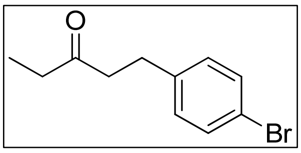

$^1$H NMR (400 MHz, CHLOROFORM-D) δ 7.38 (d, $J$ = 8.3 Hz, 2H), 7.05 (d, $J$ = 8.2 Hz, 2H), 2.85 (t, $J$ = 7.5 Hz, 2H), 2.70 (t, $J$ = 7.4 Hz, 2H), 2.40 (q, $J$ = 7.3 Hz, 2H), 1.04 (t, $J$ = 7.3 Hz, 3H). $^{13}$C NMR (101 MHz, CHLOROFORM-D) δ 210.3, 140.3, 131.6, 130.3, 120.0, 43.7, 36.3, 29.3, 7.9. IR (neat, $\nu_{C=O}$) 1712cm$^{-1}$, EI-MS: 240 (M$^+$). HRMS (ESI, m/z) calcd for $C_{11}H_{13}{}^{79}BrO$ [M+Na]$^+$: 263.0042 , found: 263.0041.

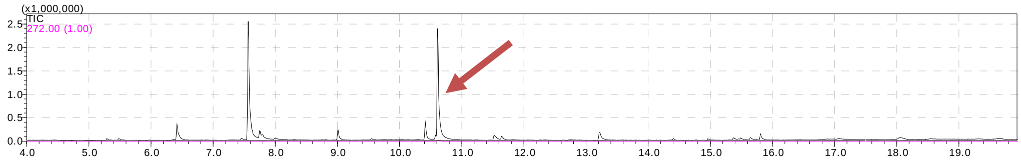

*GC-MS spectra of the extracted reaction mixture: red arrow indicated the peak of the aimed product in form of oxime without hydrolysis.*

**4-(4-bromophenyl)-3,3-dimethylbutan-2-one (4dd)**: colorless liquid; yield: 83%

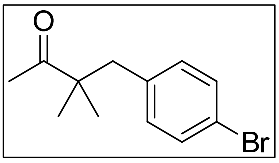


$^{1}H$ NMR (400 MHz, CHLOROFORM-D) δ 7.36 (d, *J* = 8.2 Hz, 2H), 6.95 (d, *J* = 8.3 Hz, 2H), 2.75 (s, 2H), 2.10 (s, 3H), 1.10 (s, 6H).$^{13}C$ NMR (101 MHz, CHLOROFORM-D) δ 213.6, 137.0, 132.1, 131.3, 120.5, 48.6, 44.6, 26.2, 24.5. IR (neat, $\nu_{C=O}$) 1705cm$^{-1}$, EI-MS: 254 ($M^{+}$). HRMS (ESI, m/z) calcd for $C_{12}H_{15}{}^{79}BrO$ [M+Na]$^{+}$: 277.0193 , found: 277.0190.

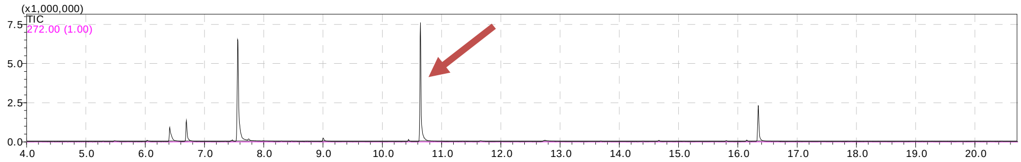


*GC-MS spectra of the extracted reaction mixture: red arrow indicated the peak of the aimed product in form of oxime without hydrolysis.*

**3-(4-bromophenyl)-2,2-dimethylpropanal (4ed)**: colorless liquid; yield: 65%

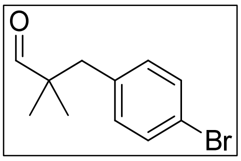


$^{1}H$ NMR (400 MHz, CHLOROFORM-D) δ 9.56 (s, 1H), 7.39 (d, *J* = 8.2 Hz, 2H), 6.97 (d, *J* = 8.3 Hz, 2H), 2.73 (s, 2H), 1.04 (s, 6H).$^{13}C$ NMR (101 MHz, CHLOROFORM-D) δ 205.7 136.1 132.1 131.4, 120.7, 47.0, 42.5 21.5. IR (neat, $\nu_{C=O}$) 1736cm$^{-1}$. EI-MS: 240 ($M^{+}$). HRMS (ESI, m/z) calcd for $C_{11}H_{13}{}^{79}BrO$ [M+Na]$^{+}$: 263.0042 , found: 263.0044.

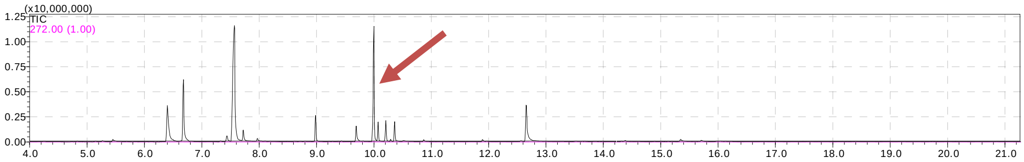


*GC-MS spectra of the extracted reaction mixture: red arrow indicated the peak of the aimed product in form of oxime without hydrolysis*

**1-(4-bromophenyl)-2,4-dimethylpentan-3-one (4fd)**: colorless liquid: 85%

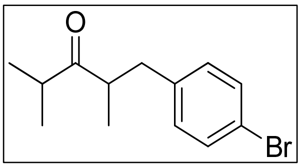


$^{1}H$ NMR (301 MHz, CHLOROFORM-D) δ 7.37 (d, *J* = 8.3 Hz, 2H), 7.00 (d, *J* = 8.3 Hz, 2H), 3.05 – 2.83 (m, 2H), 2.64 – 2.42 (m, 2H), 1.07 (d, *J* = 6.6 Hz, 3H), 1.01 (d, *J* = 6.9 Hz, 3H), 0.87 (d, *J* = 6.9 Hz, 3H). $^{13}C$ NMR (76 MHz, CHLOROFORM-D) δ 217.5, 139.2, 131.5, 130.9, 120.1, 46.5, 40.43 (s), 38.9, 18.1, 18.0, 17.4. IR (neat, $\nu_{C=O}$) 1709 cm$^{-1}$, EI-MS: 268 ($M^{+}$). HRMS (ESI, m/z) calcd for $C_{12}H_{17}{}^{79}BrO$ [M+Na]$^{+}$: 291.0355 , found: 291.0343.

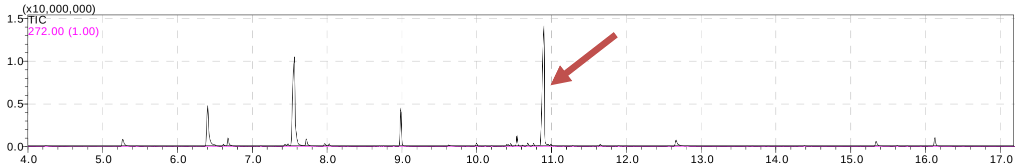


*GC-MS spectra of the extracted reaction mixture: red arrow indicated the peak of the aimed product in form of oxime without hydrolysis.*

**1-(2-(4-bromophenyl)cyclohexyl)ethanone (4gd)**: colorless liquid; yield: 42%

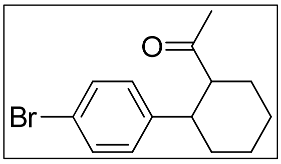


$^1$H NMR (400 MHz, CHLOROFORM-D) δ 7.38 (d, *J* = 8.3 Hz, 2H), 7.05 (d, *J* = 8.3 Hz, 2H), 2.84 – 2.64 (m, 2H), 1.99 – 1.85 (m, 4H), 1.84 (s, 3H), 1.48 – 1.35 (m, 4H).$^{13}$C NMR (101 MHz, CHLOROFORM-D) δ 211.8, 144.1, 131.7, 129.2, 120.2, 57.3, 45.7 34.4, 30.1, 29.7, 26.3, 25.6. IR (neat, $\nu_{C=O}$) 1706 cm$^{-1}$, EI-MS: 280 (M$^+$). HRMS (ESI, m/z) calcd for $C_{14}H_{17}{}^{79}BrO$ [M+Na]$^+$: 303.0355 , found: 303.0352.

**1-(4'-bromo-[1,1'-biphenyl]-2-yl)propan-1-one (4od):** colorless liquid; yield: 64%

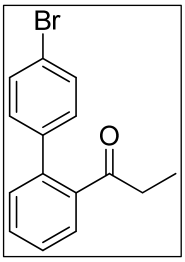


$^1$H NMR (400 MHz, CHLOROFORM-D) δ 7.55 (d, *J* = 8.3 Hz, 2H), 7.49 (d, *J* = 9.0 Hz, 2H), 7.43 (d, *J* = 8.1 Hz, 1H), 7.35 (d, *J* = 6.9 Hz, 1H), 7.19 (d, *J* = 8.4 Hz, 2H), 2.35 (qd, *J* = 7.2, 2.5 Hz, 2H), 0.94 (tt, *J* = 17.4, 8.7 Hz, 3H). $^{13}$C NMR (101 MHz, CHLOROFORM-D) δ 208.2, 140.9, 139.8, 138.9, 131.9, 130.7, 130.2, 130.2, 128.0, 127.9, 122.3, 36.3, 8.7. IR (neat, $\nu_{C=O}$) 1706 cm$^{-1}$, EI-MS: 288 (M$^+$). HRMS (ESI, m/z) calcd for $C_{15}H_{13}{}^{79}BrO$ [M+Na]$^+$: 311.0042 , found: 303.0039, 313.0013

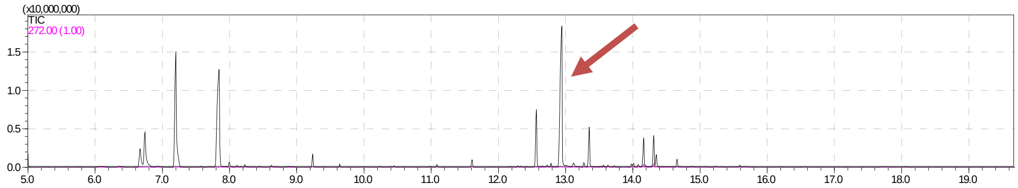


*GC-MS spectra of the extracted reaction mixture: red arrow indicated the peak of the aimed product in form of oxime without hydrolysis.*

**ethyl 1-(4-bromobenzyl)-2-oxocyclohexanecarboxylate (4nd):** colorless solid; yield: 80%, m.p.65°C

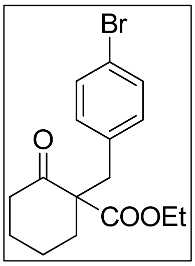


$^1$H NMR (400 MHz, CHLOROFORM-D) δ 7.35 (d, *J* = 7.6 Hz, 2H), 7.01 (d, *J* = 7.8 Hz, 2H), 4.17 – 4.01 (m, 2H), 3.23 (d, *J* = 13.8 Hz, 1H), 2.82 (d, *J* = 13.8 Hz, 1H), 2.44 (qd, *J* = 13.1, 6.1 Hz, 3H), 2.02 (dd, *J* = 6.5, 3.8 Hz, 1H), 1.80 – 1.59 (m, 3H), 1.43 (td, *J* = 12.7, 4.5 Hz, 1H), 1.17 (t, *J* = 7.1 Hz, 3H).

$^{13}$C NMR (101 MHz, CHLOROFORM-D) δ 207.3, 171.1, 135.8, 132.3, 131.2, 120.8, 62.2, 61.5, 41.4, 40.0, 36.3, 27.7, 22.7, 14.1.IR (neat, $\nu_{C=O}$) 1709cm$^{-1}$, 1722 cm$^{-1}$, EI-MS: 288 (M$^+$). HRMS (ESI, m/z) calcd for $C_{16}H_{19}{}^{79}BrO_3$ [M+Na]$^+$: 361.0410 , found: 361.0407, 363.0380

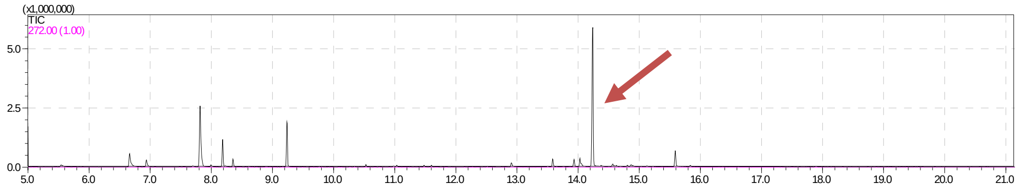


*GC-MS spectra of the extracted reaction mixture: red arrow indicated the peak of the aimed product in form of oxime without hydrolysis.*

**(*8R,9S,10S,13S,14S,17S*)-2-(4-bromobenzyl)-17-hydroxy-10,13,17-trimethyltetradecahydro-1H-cyclopenta[a]phenanthren-3(2H)-one (4md):** colorless solid; yield: 81%; m.p.186-188°C

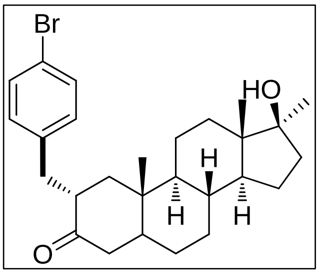


$^1$H NMR (301 MHz, CHLOROFORM-D) δ 7.45 – 7.33 (m, 2H), 7.03 (d, *J* = 8.3 Hz, 2H), 3.19 (dd, *J* = 14.0, 5.0 Hz, 1H), 2.59 (td, *J* = 13.0, 5.5 Hz, 1H), 2.11 (dd, *J* = 14.0, 3.6 Hz, 1H), 1.97 (dd, *J* = 13.0, 5.8 Hz, 1H), 1.88 – 1.65 (m, 4H), 1.59 – 1.41 (m, 6H), 1.39 – 1.25 (m, 7H), 1.20 (s, 3H), 1.11 – 1.03 (m, 1H), 1.00 (s, 3H), 0.90 (t, *J* = 5.4 Hz, 1H), 0.82 (d, *J* = 9.9 Hz, 3H), 0.74 – 0.61 (m, 1H)..$^{13}$C NMR (76 MHz, CHLOROFORM-D) δ 211.7, 139.6, 131.5, 131.0, 119.8, 81.8, 54.0, 50.6, 48.4, 48.2, 46.0, 45.7, 45.1, 39.1, 36.6, 36.2, 34.9, 31.6, 31.7, 28.7, 26.0, 23.4, 21.3, 14.1, 12.6. IR (neat, $\nu_{C=O}$) 1711cm$^{-1}$.HRMS (ESI, m/z) calcd for $C_{27}H_{37}{}^{79}BrO_2$ [M+Na]$^+$: 497.1853 , found: 497.1851.$[\alpha]_D^{23}$-24.7° (1, DCM)

## V. Experimental Procedure of hydrogenation

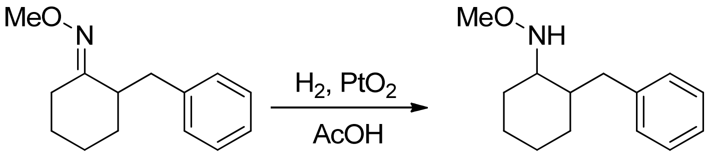


**3aa** **3aa-H**: 72%

($E$)-2-benzylcyclohexanone $O$-methyl oxime, **3aa** (108.3 mg, 0.5 mmol) in acetic acid (5 mL) was hydrogenated over platinum(IV) oxide (11.3 mg, 0.05 mmol) under hydrogen pressure (5 bar) for 24h. Then, the mixture filtered through celite and washed between dichloromethane and aqueous $Na_2CO_3$ solution. The organic layer was concentrated purified on silica gel (petroleum ether/ethyl acetate 100/1) to give provide **3aa-H** (78.8 mg, 0.36 mmol, 72%) as a colorless liquid.

**N-(2-benzylcyclohexyl)-O-methylhydroxylamine (3aa-H)**: colorless liquid; Yield: 72%

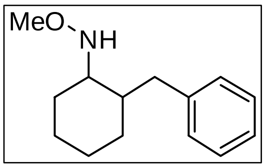


$^{1}$H NMR (400 MHz, CHLOROFORM-D) δ 7.29 – 7.24 (m, 2H), 7.21 – 7.15 (m, 3H), 3.56 (s, 3H), 3.10 (dt, $J$ = 7.1, 3.4 Hz, 1H), 2.79 (dd, $J$ = 13.5, 5.5 Hz, 1H), 2.49 (dd, $J$ = 13.4, 9.9 Hz, 1H), 2.08 – 1.93 (m, 1H), 1.74 – 1.64 (m, 2H), 1.62 – 1.57 (m, 1H), 1.44 – 1.23 (m, 6H). $^{13}$C NMR (101 MHz, CHLOROFORM-D) δ 141.6, 129.2, 128.3, 125.8, 62.2, 59.5, 40.3, 35.7, 27.1, 23.5, 22.6.IR (neat, $\nu_{N-H,}$ $\nu_{C-N}$) 2928, 1452 cm$^{-1}$, EI-MS: 219 ($M^+$), HRMS (ESI, m/z) calcd for $C_{14}H_{21}NO$ $[M+H]^+$: 220.1695 , found: 220.1690.

## VI. Palladium Complex[7]

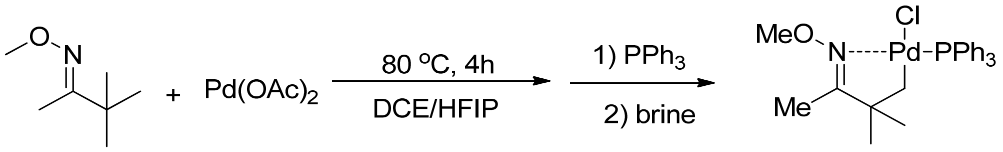


A schlenk tube was charged with $Pd(OAc)_2$ (157 mg, 0.7 mmol) and a solution of (*E*)-3,3-dimethylbutan-2-one O-methyl oxime (64.5 mg, 0.5 mmol) in dichloroethane (1.5 mL) and HFIP (0.5 mL). The tube was sealed and stirrd at 85°C for 4h. After this time the reaction mixture was cooled to room temperature and treated with triphenylphosphine (466 mg, 0.7 mmol). The tube was sealed 80 ˚C for another 2 hours. After this time the reaction mixture was cooled to room temperature and filtered through a thin pad of Celite, eluting with ethyl acetate (30 mL) and $CH_2Cl_2$ (10 mL) and the filtrate was concentrated under reduced pressure. The residue was then re-dissolved in $CH_2Cl_2$ (20 mL), washed with brine (2 x 20 mL) and dried over $Mg_2SO_4$ and concentrated under vacuum. The crude reaction mixture was purified by flash column chromatography (silica gel, gradient elution: 0% acetone in $CH_2Cl_2$ to 2.5% acetone in $CH_2Cl_2$) to provide the complex as a colorless solid (236mg, 89%). Crystals were grown by vial-in-vial diffusion of ether into dichloromethane solution of the title complex.

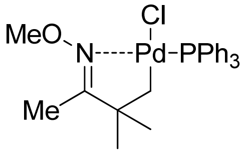


NMR: $^1$H NMR (400 MHz, CHLOROFORM-D) δ 7.78 – 7.67 (m, 6H), 7.47 – 7.34 (m, 9H), 4.15 (s, 3H), 1.95 (s, 3H), 1.51 (d, *J* = 3.8 Hz, 2H), 1.06 (s, 6H).$^{13}$C NMR (101 MHz, CHLOROFORM-D) δ 183.4, 134.7, 134.6, 131.8, 131.3, 130.3 128.3, 128.2, 62.8, 48.8, 46.1, 29.1, 27.0, 12.7

**X-ray crystal structure analysis of compound 6:** Single crystals suitable for X-ray analysis were obtained by slow evaporation of its solution in hexane. The crystal structure has been deposited at the Cambridge Crystallographic Data Centre and allocated the deposition number: **CCDC 1048079.** Formula: $C_{15}H_{29}ClNOPPd$, $M$ = 532.31, colourless crystal, 0.33x 0.21 x 0.11 mm, $a$ = 16.151(3), $b$ = 9.5824(19), $c$ = 16.403(3) Å, α = 90.00, β =111.38(3), γ = 90.00 , $V$ = 2363.9(8) Å$^3$, $\rho_{calc}$ = 1.496 gcm$^{-3}$, μ = 0.982 mm$^{-1}$, $Z$ = 4, Monoclinic, space group P2(1)/n, λ = 0.71073 Å, $T$ = 173(2) K. Data completeness = 0.985, Theta (max) = 25.00

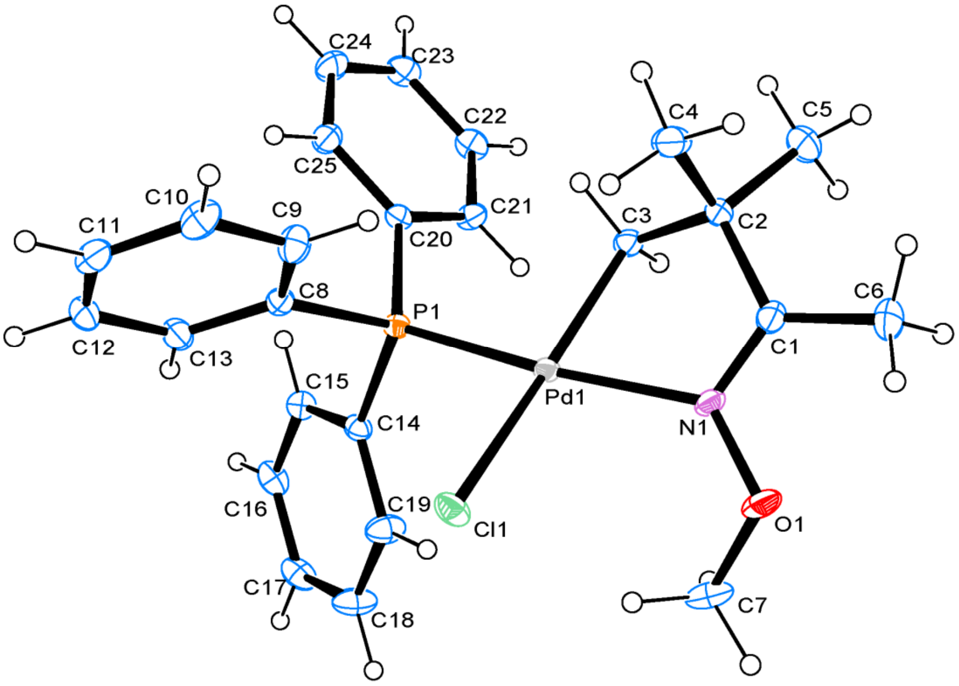

## VII. Discussions on Negative Results

### 1. Application of diaryliodonium salts bearing heteroaromatic rings

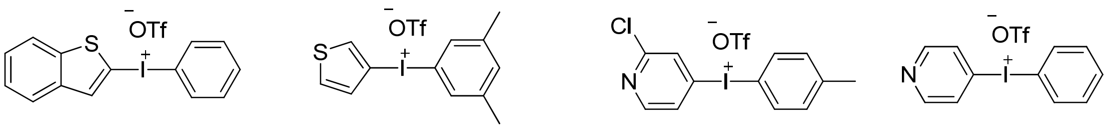

**2m** **2n** **2o** **2p**

When **2m** was employed to react with **1a**, the transformation of phenyl group was observed to generate **3aa.** As to **2n**, it is noted in the manuscript that asymmetric diaryliodonium salts Ar-$I^+$-Mes$X^-$ are not applicable in this reaction. And for **2o** and **2p**, no desired products were obtained probably due to the coordination of pyridine group with palladium catalysts.

### 2. Examination of aromatic oxime ether under the standard condition

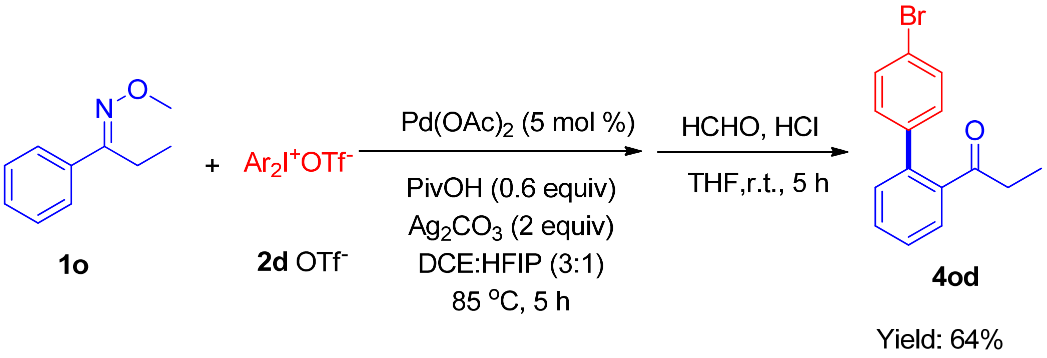


### 3. Results of the methylene C-H bond activation.

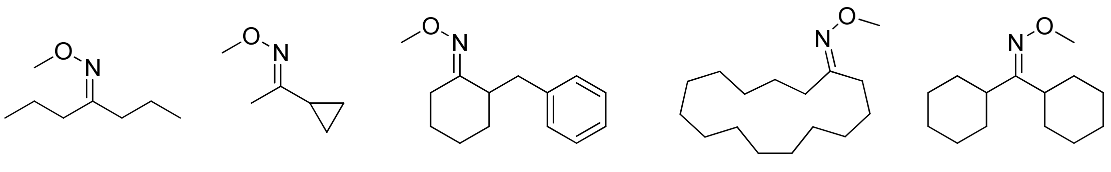

**1p** **1q** **1r** **1s** **1t**

Listed above are the oximes we examined under the optimized condition of this method with diaryliodonium salts **2d**. **1q-1s** were not be able be arylated while the arylated product of **1t** was observed with trace amounts in GC/MS. The scope of substrates is somehow limited for arylation on methylene C-H bond and it likely depends on the angle between the directing groups and aimed activated bonds. **1g** and **1h** can be arylated in only moderate yields since the convertion rate of starting materials were comparably low. We failed to further increase the yield by adding legands, additives, etc., while it helped a little by using more diaryliodonium salts as well as higher reacting temperature.

# VIII．Copies of $^{1}$H and $^{13}$C NMR spectra

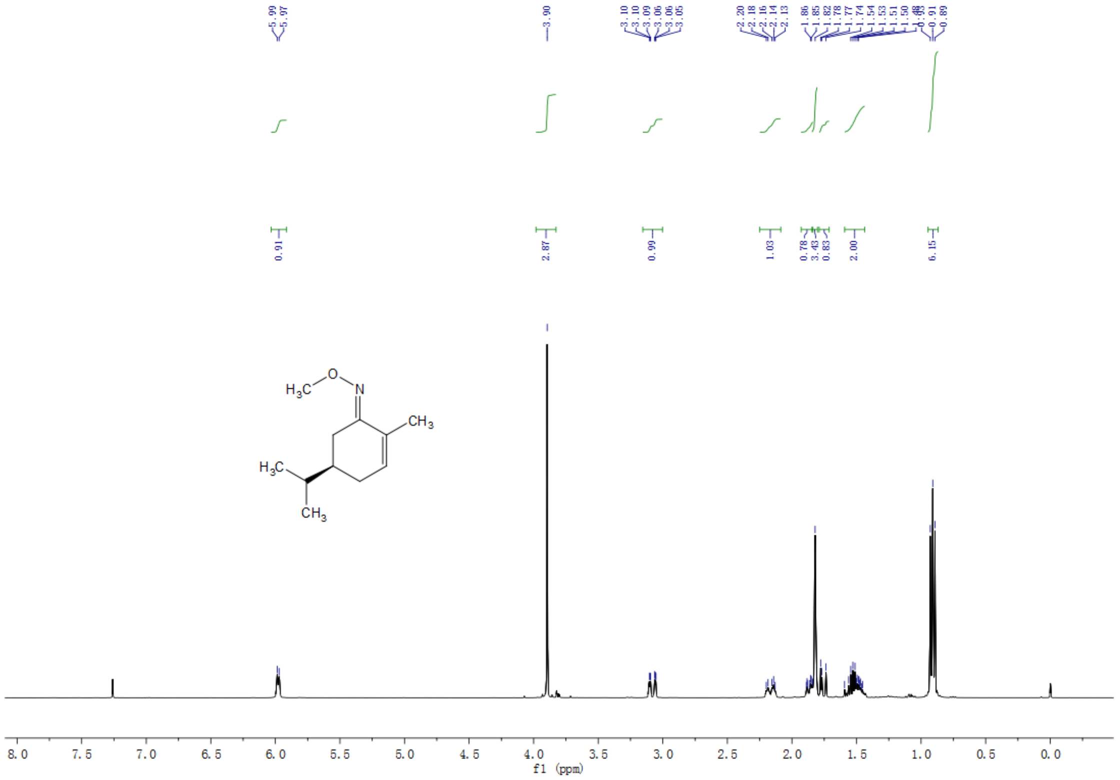


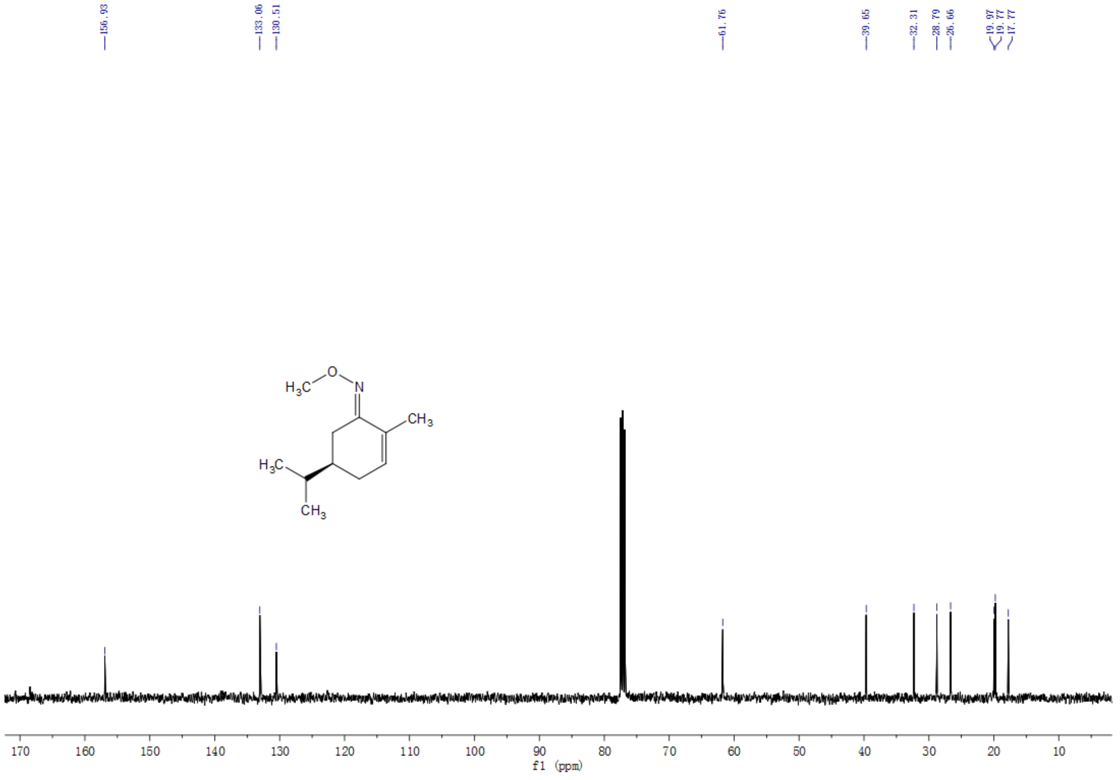


$^{1}$H NMR and $^{13}$C NMR spectra of compound **1j**

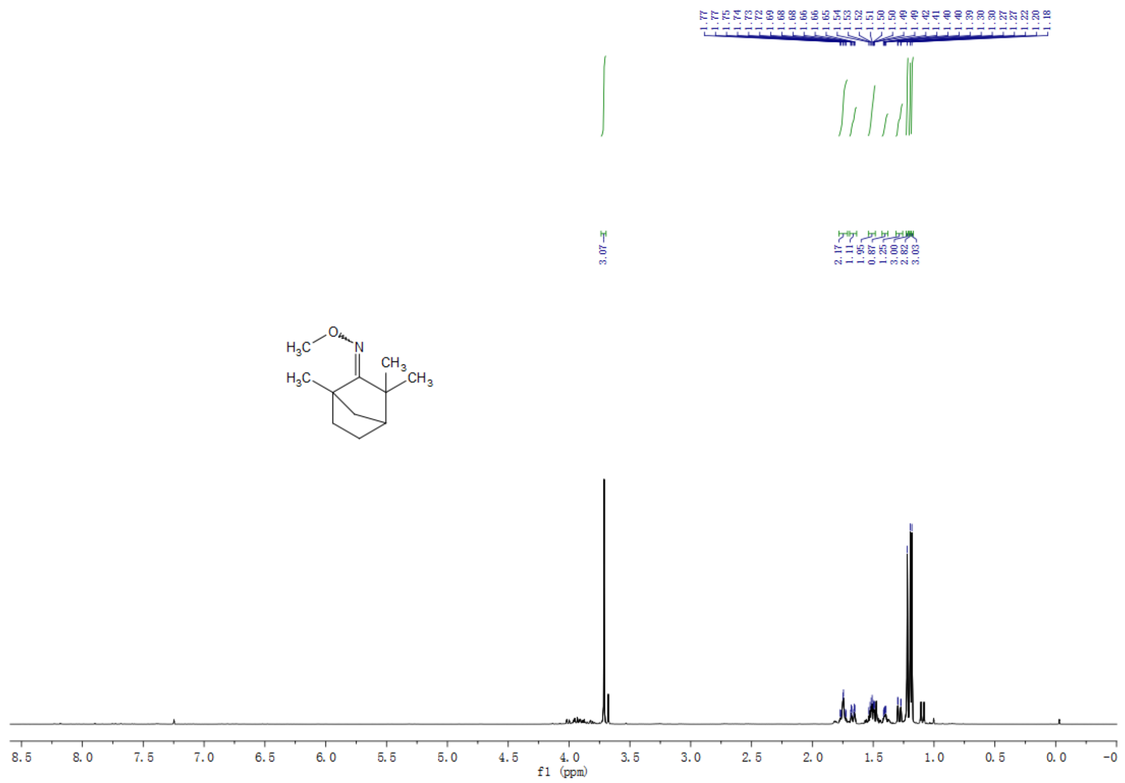

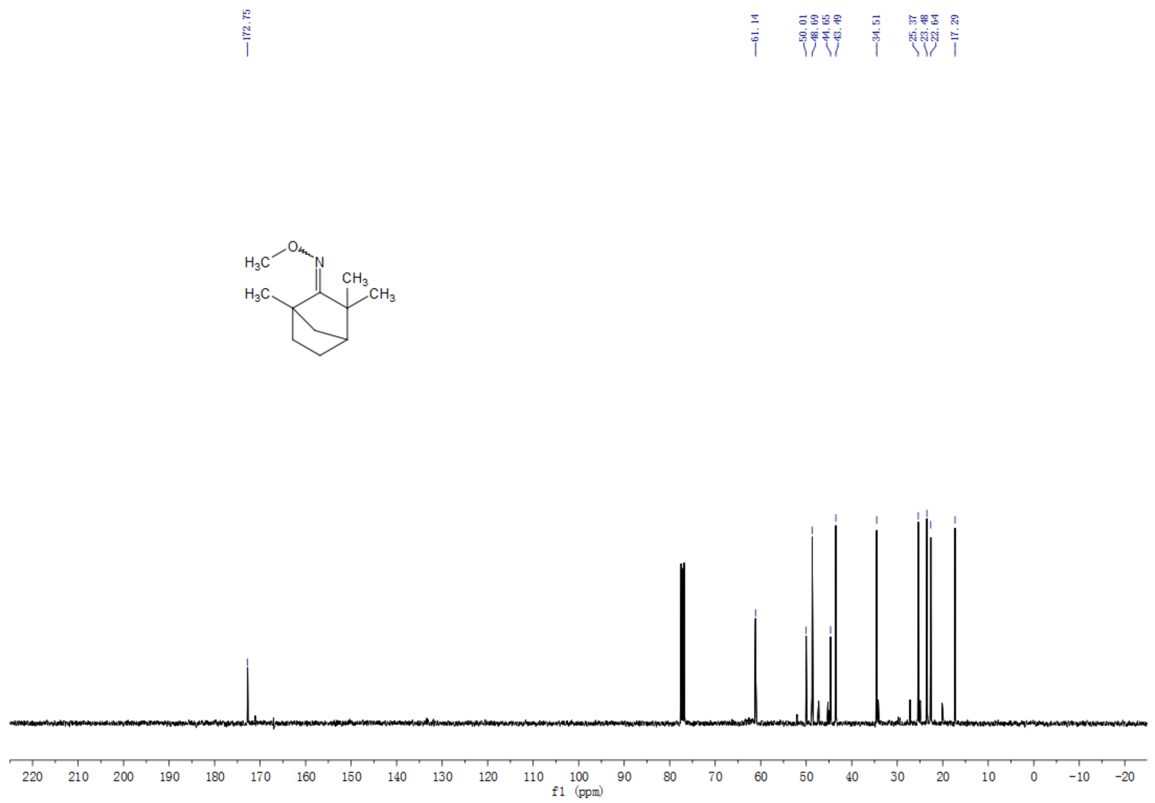

$^1$H NMR and $^{13}$C NMR spectra of compound **1i**

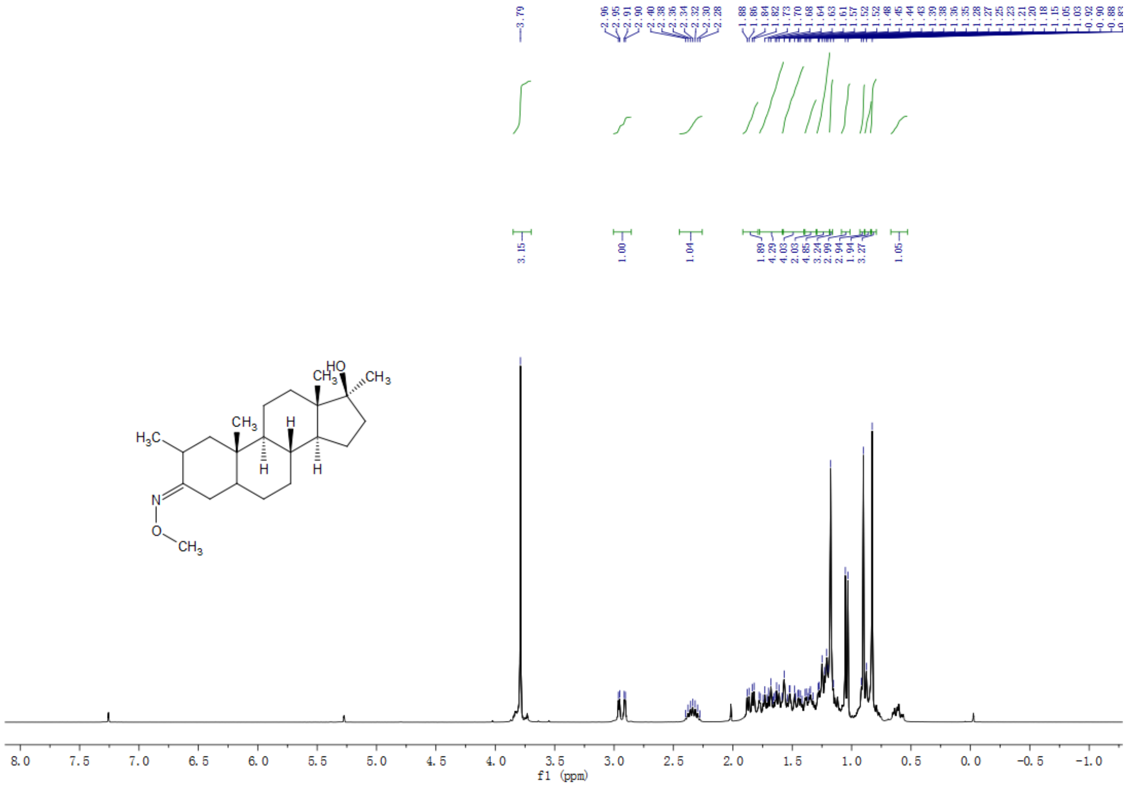


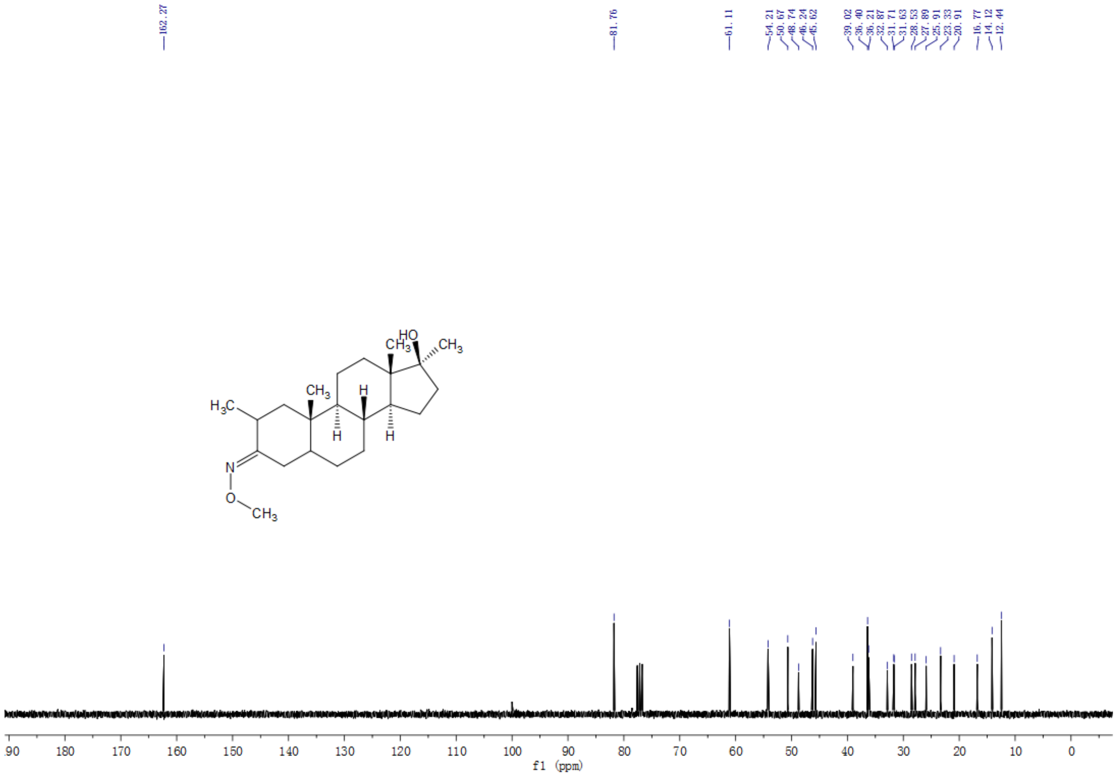


$^{1}H$ NMR and $^{13}C$ NMR spectra of compound **1m**

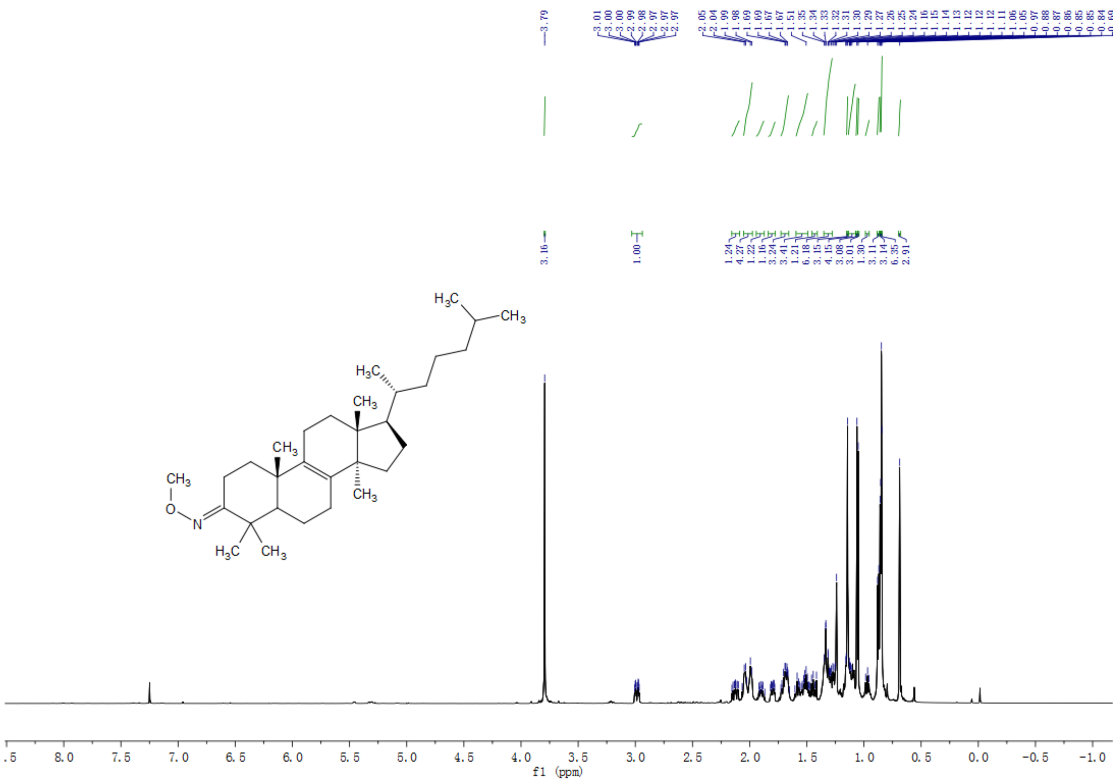

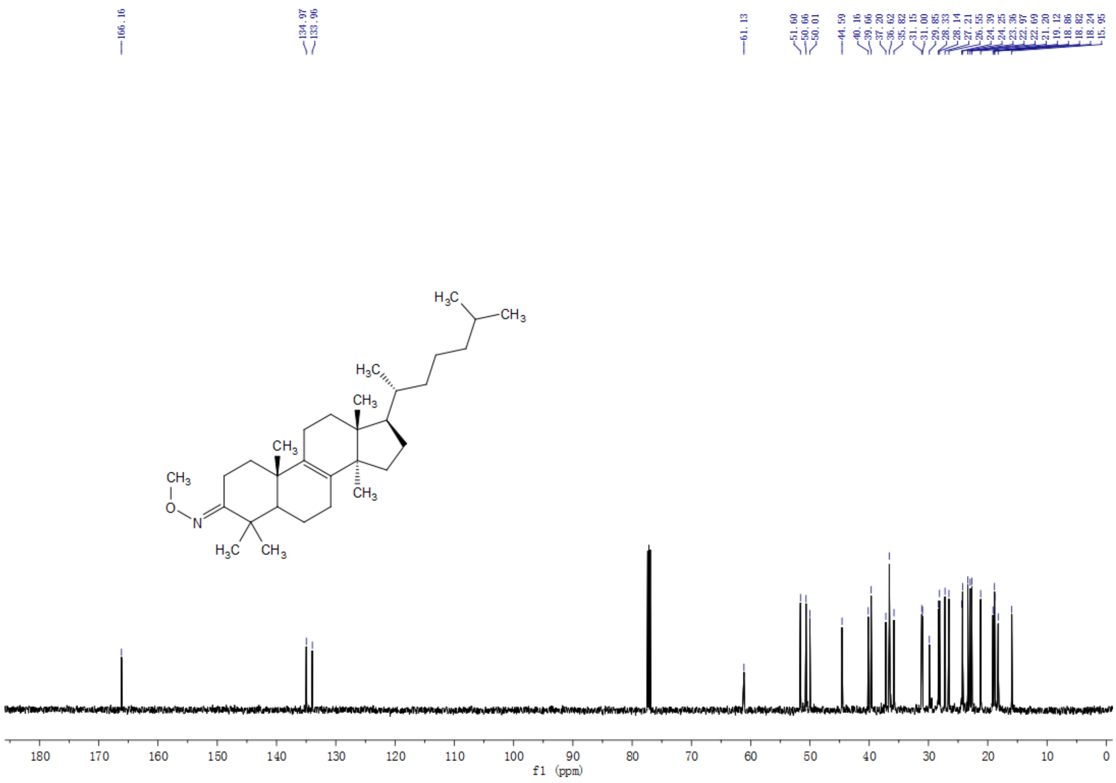

$^{1}H$ NMR and $^{13}C$ NMR spectra of compound **1k**

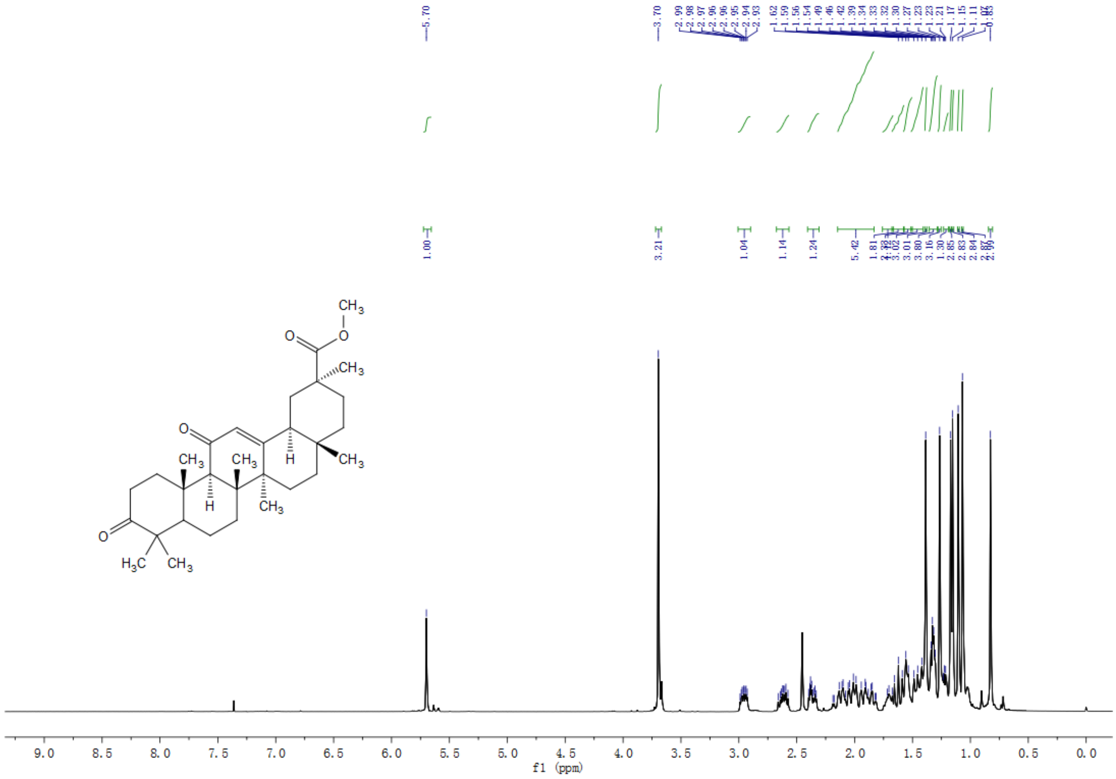

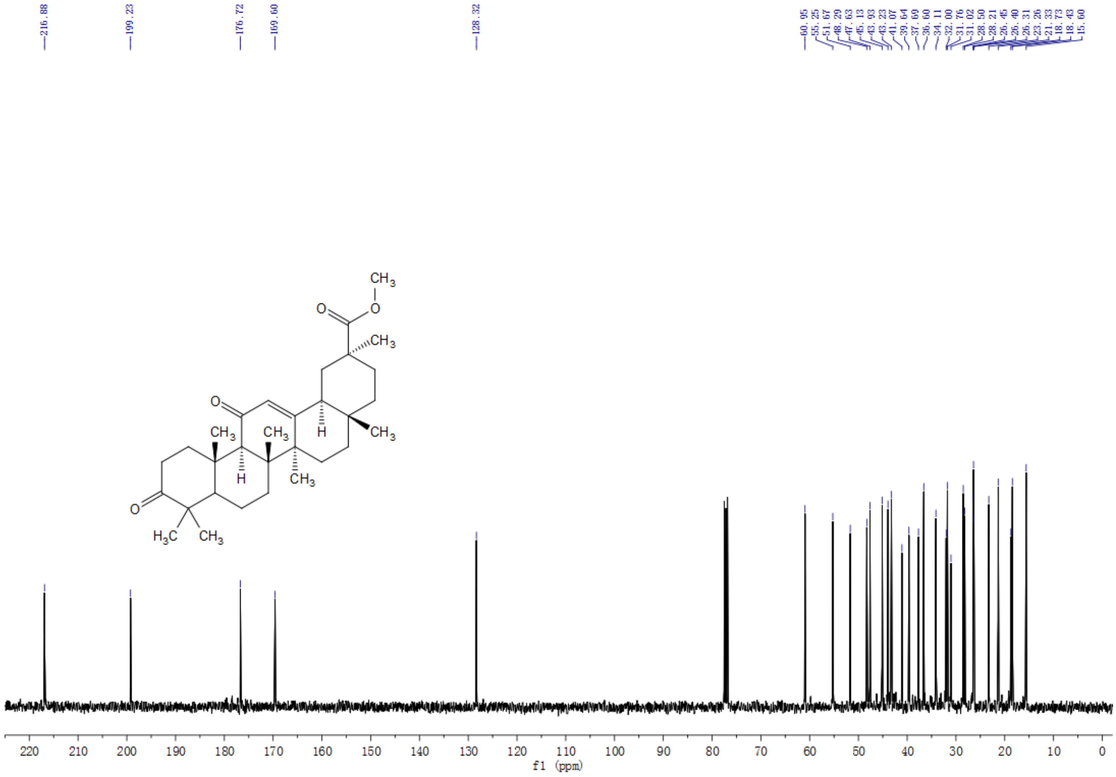

$^{1}$H NMR and $^{13}$C NMR spectra of compound **1l'**

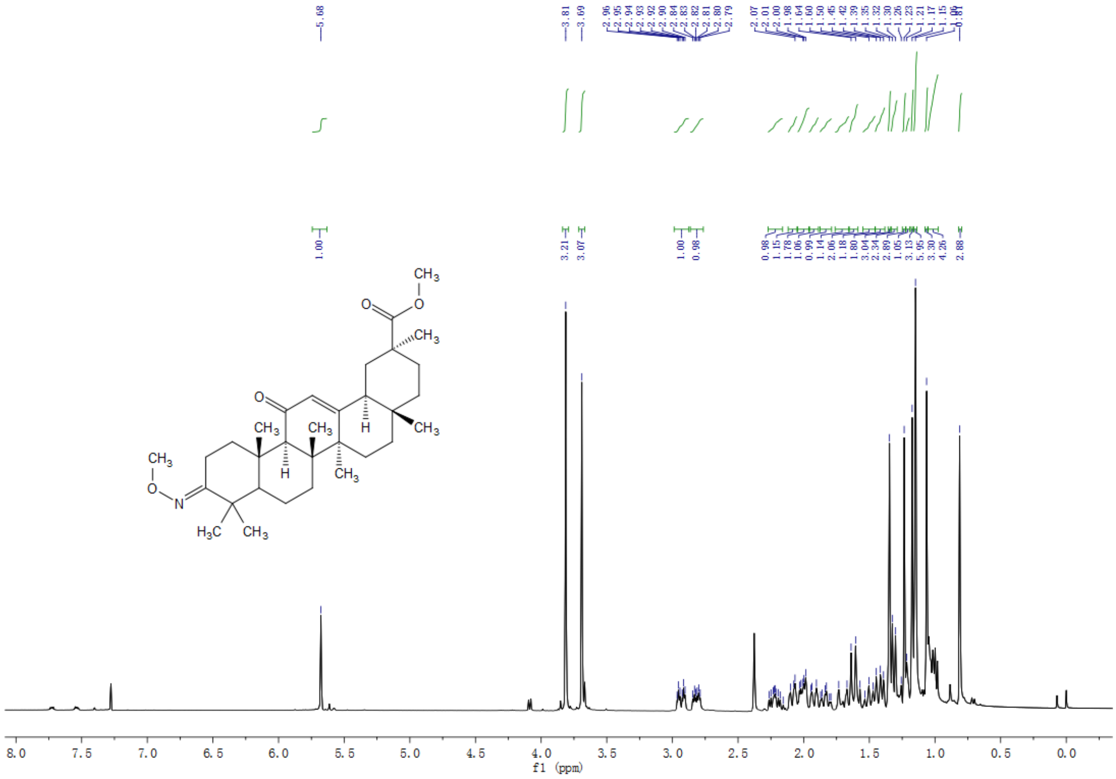

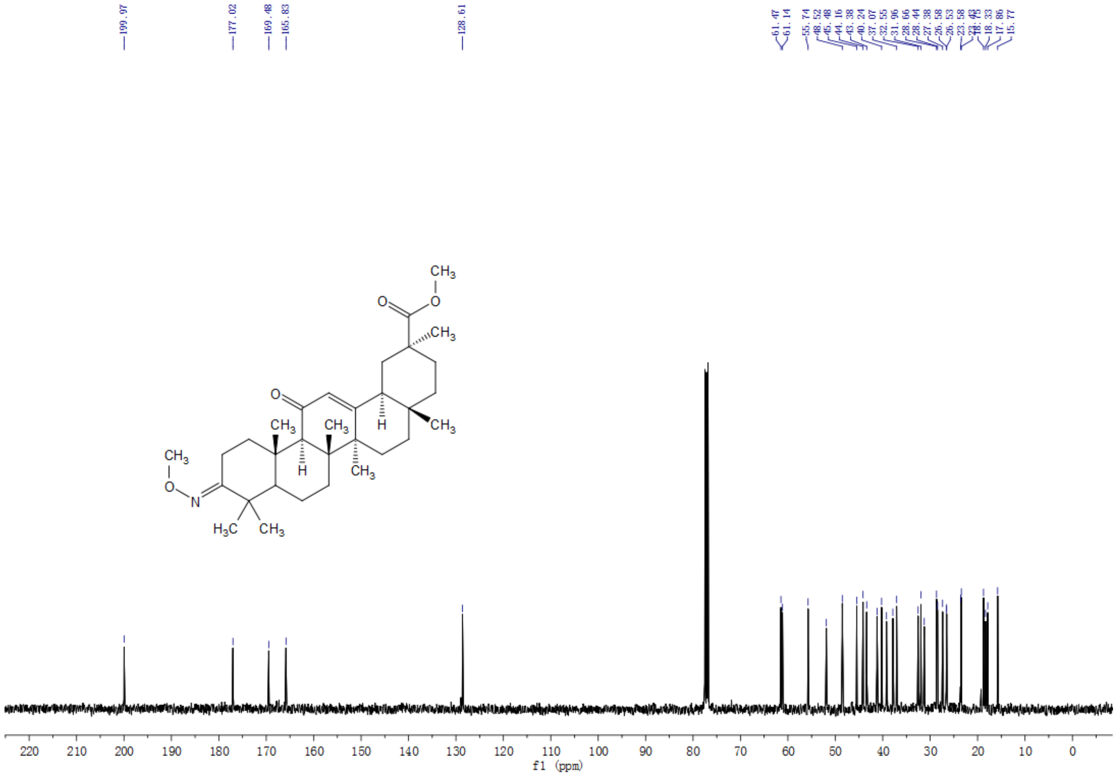

$^1$H NMR and $^{13}$C NMR spectra of compound **1l**

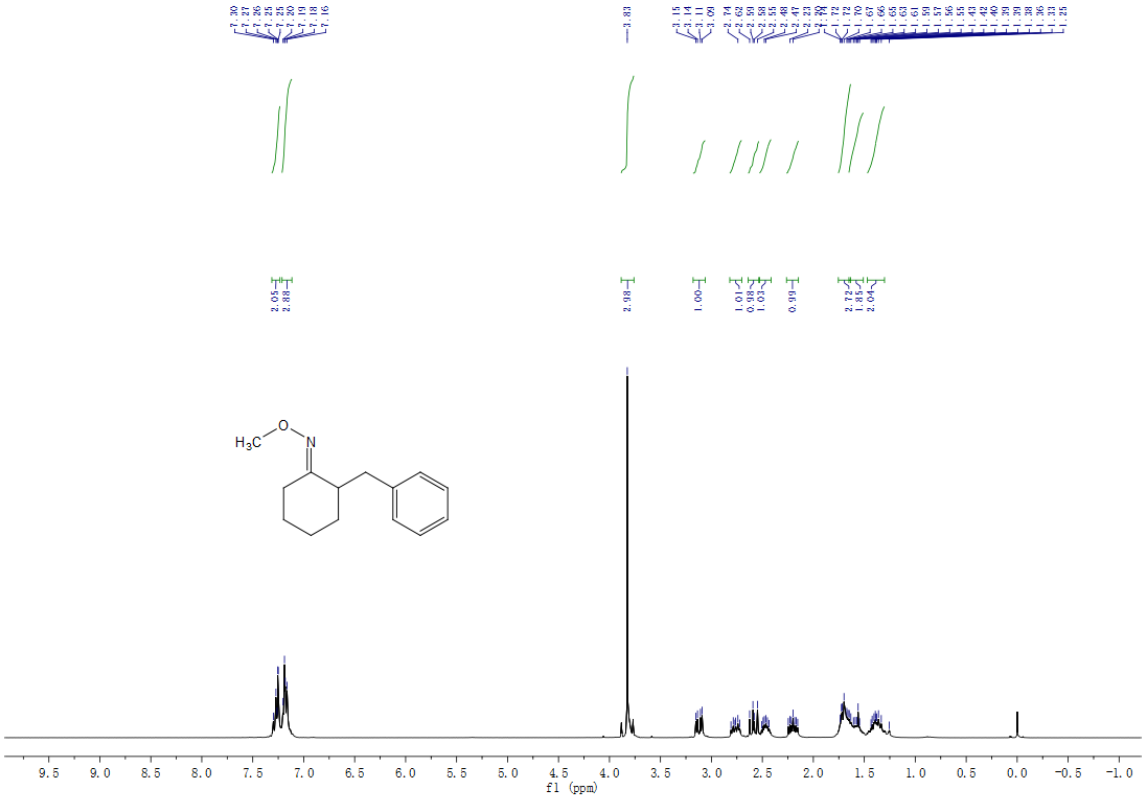


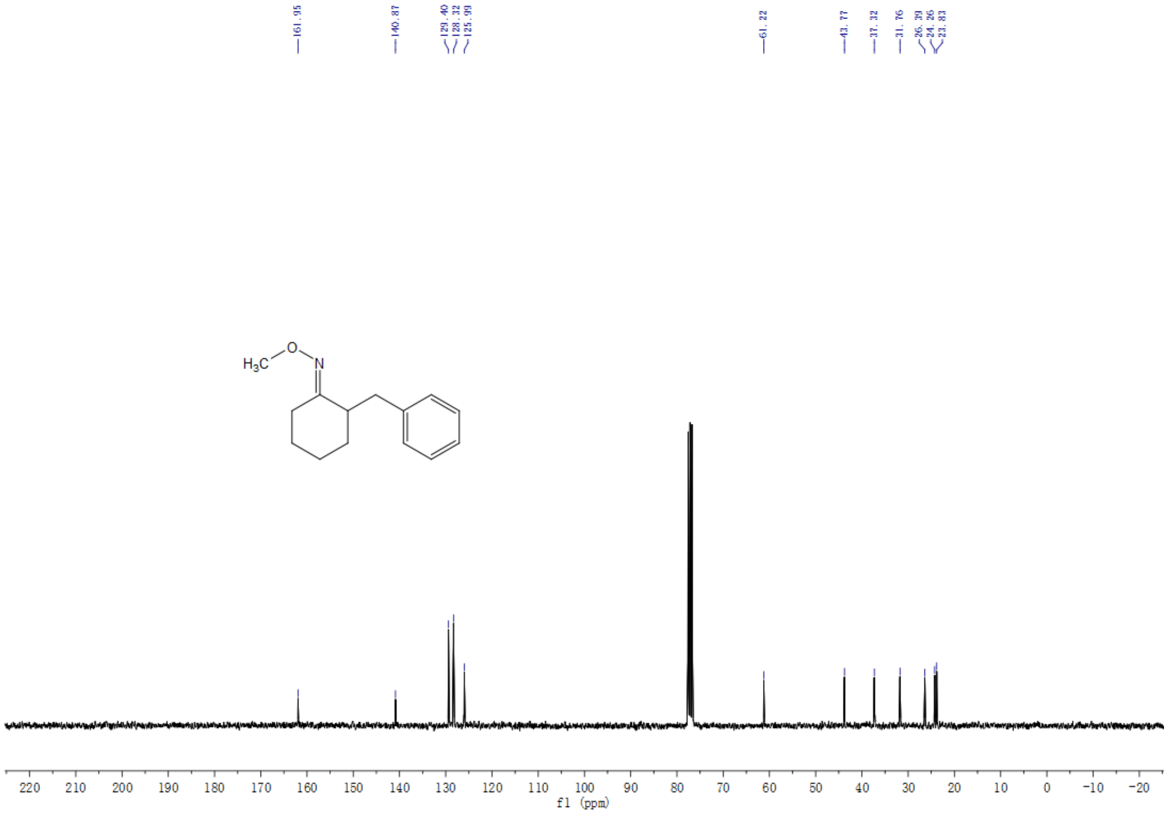


$^{1}$H NMR and $^{13}$C NMR spectra of compound **3aa**

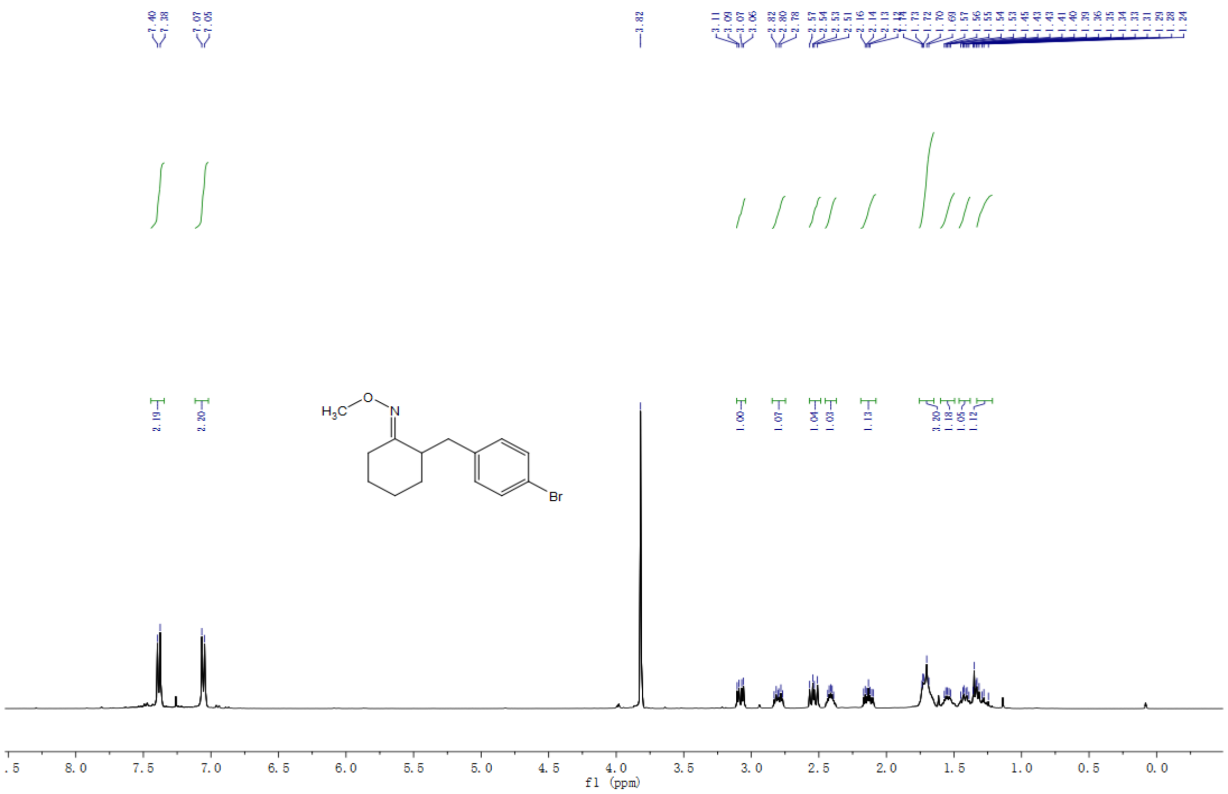

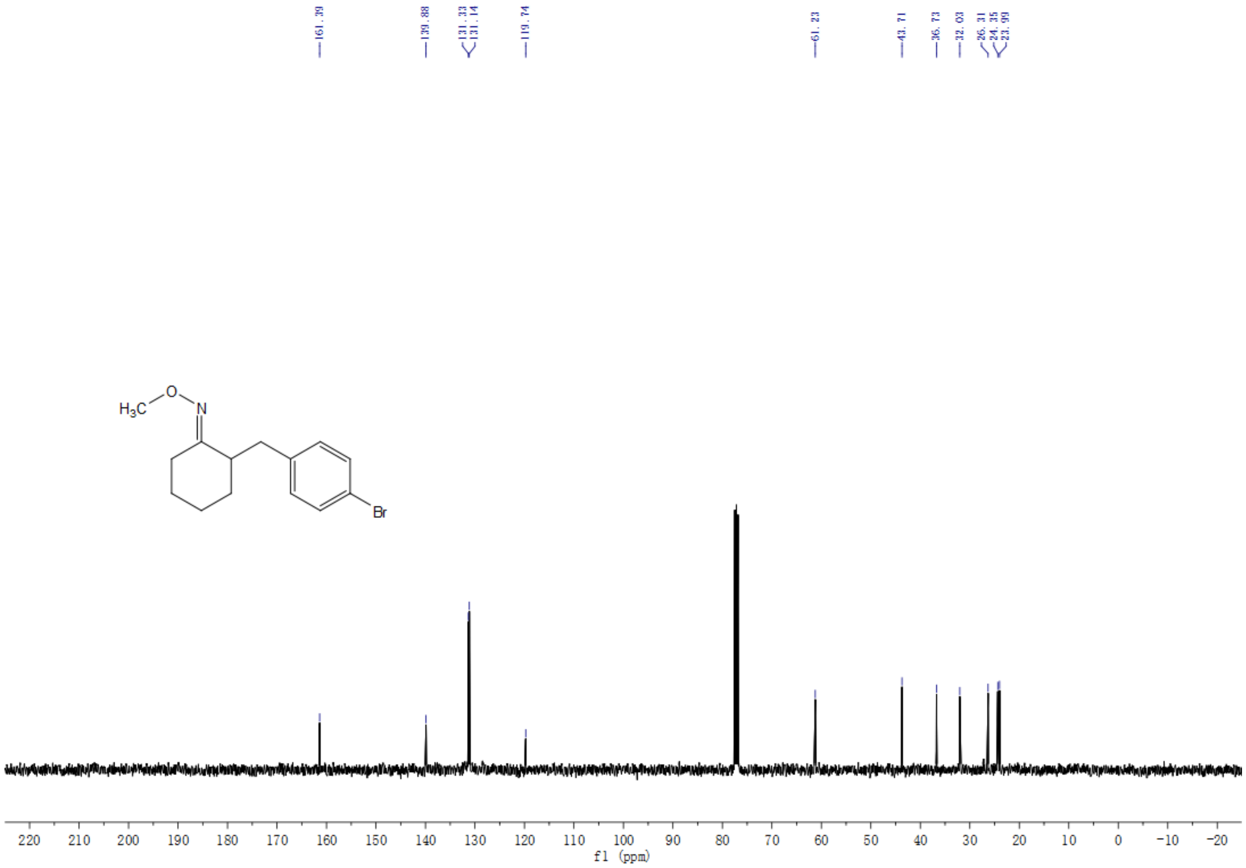

$^{1}$H NMR and $^{13}$C NMR spectra of compound **3ad**

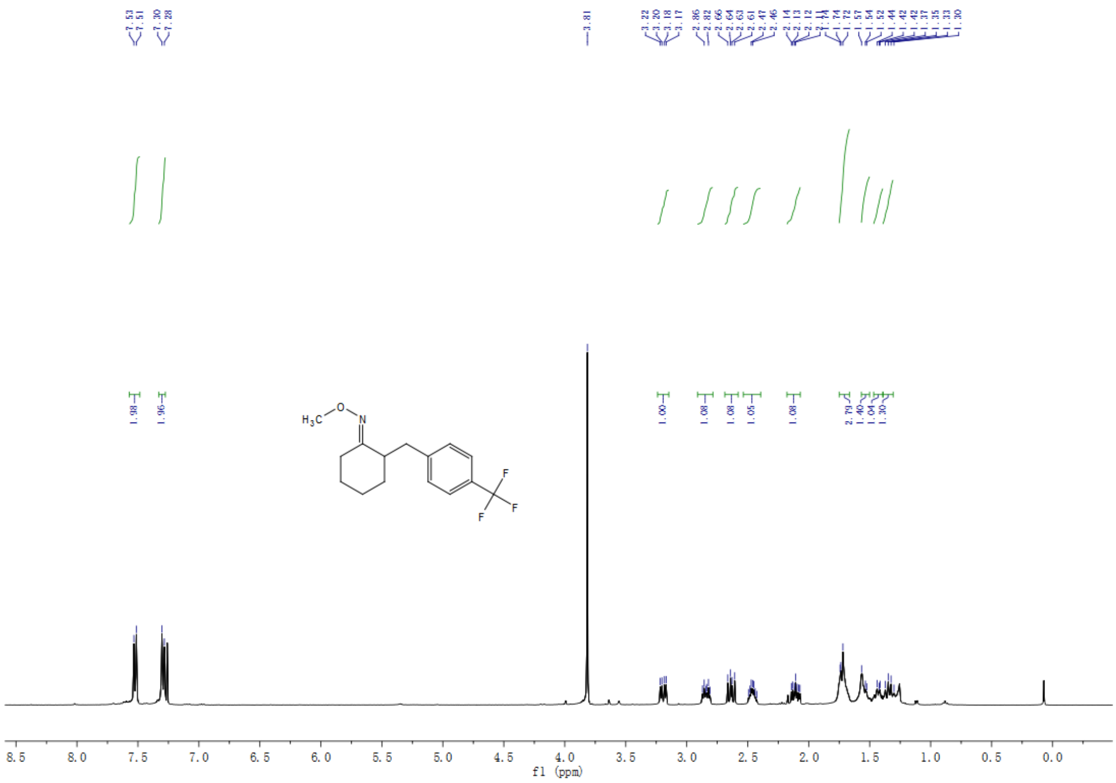

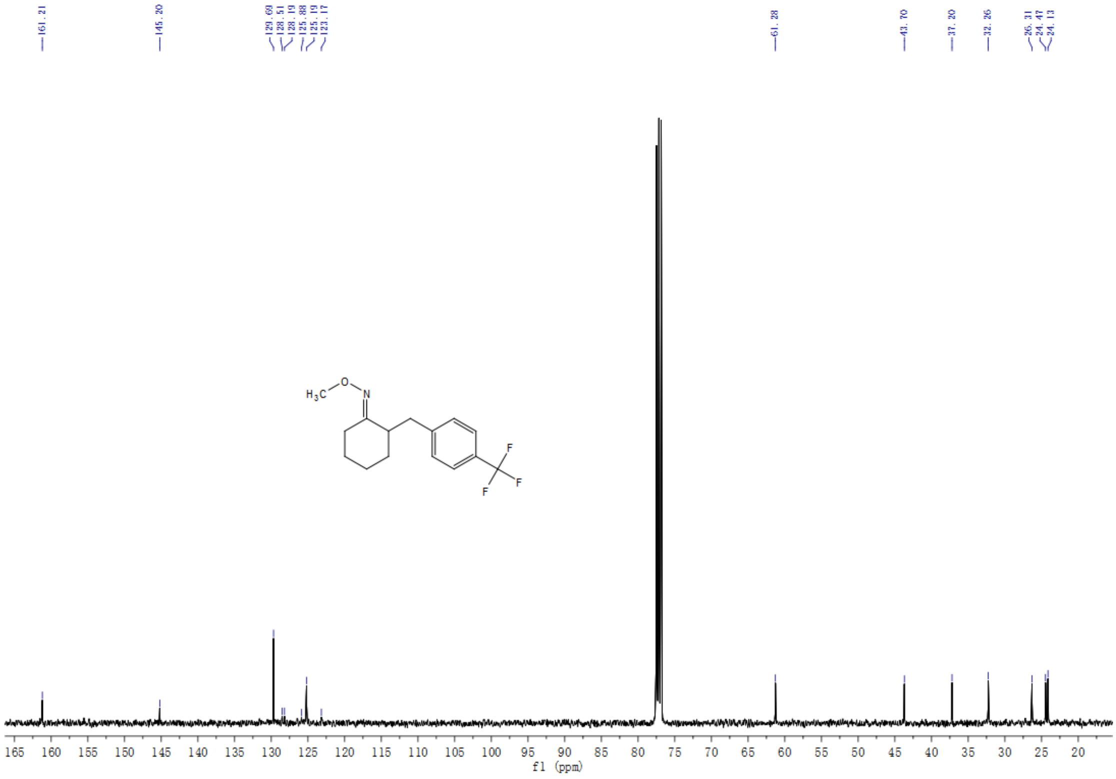

$^{1}H$ NMR and $^{13}C$ NMR spectra of compound **3ah**

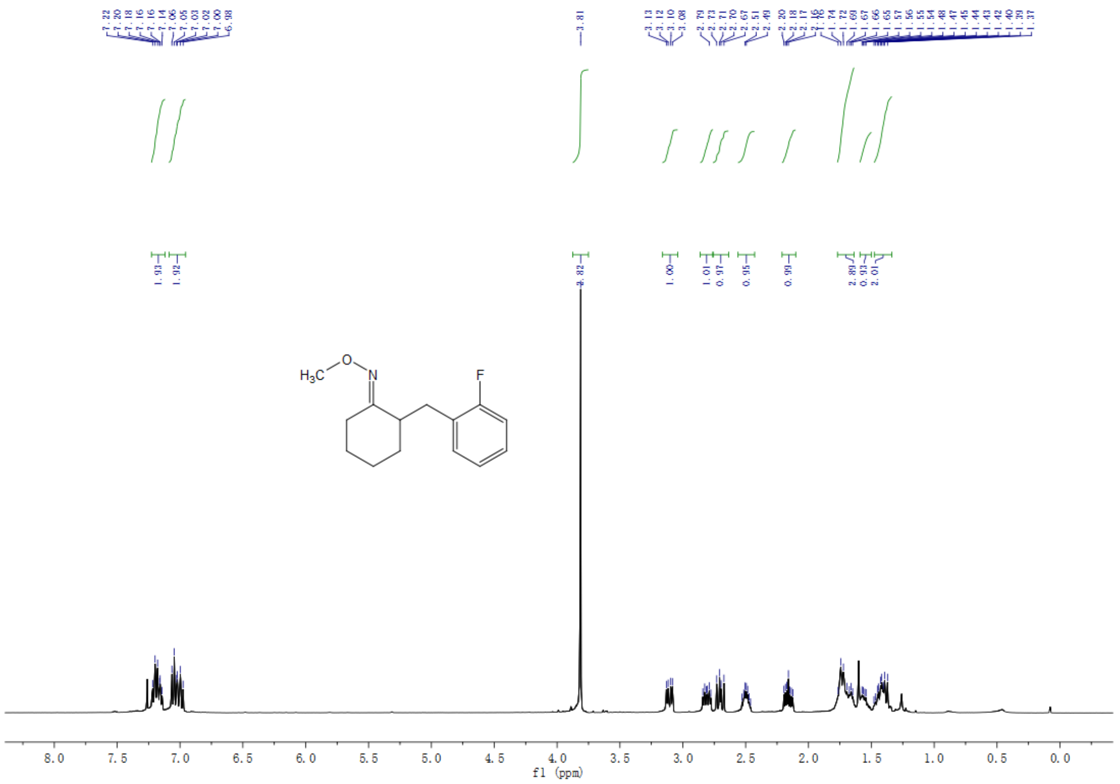

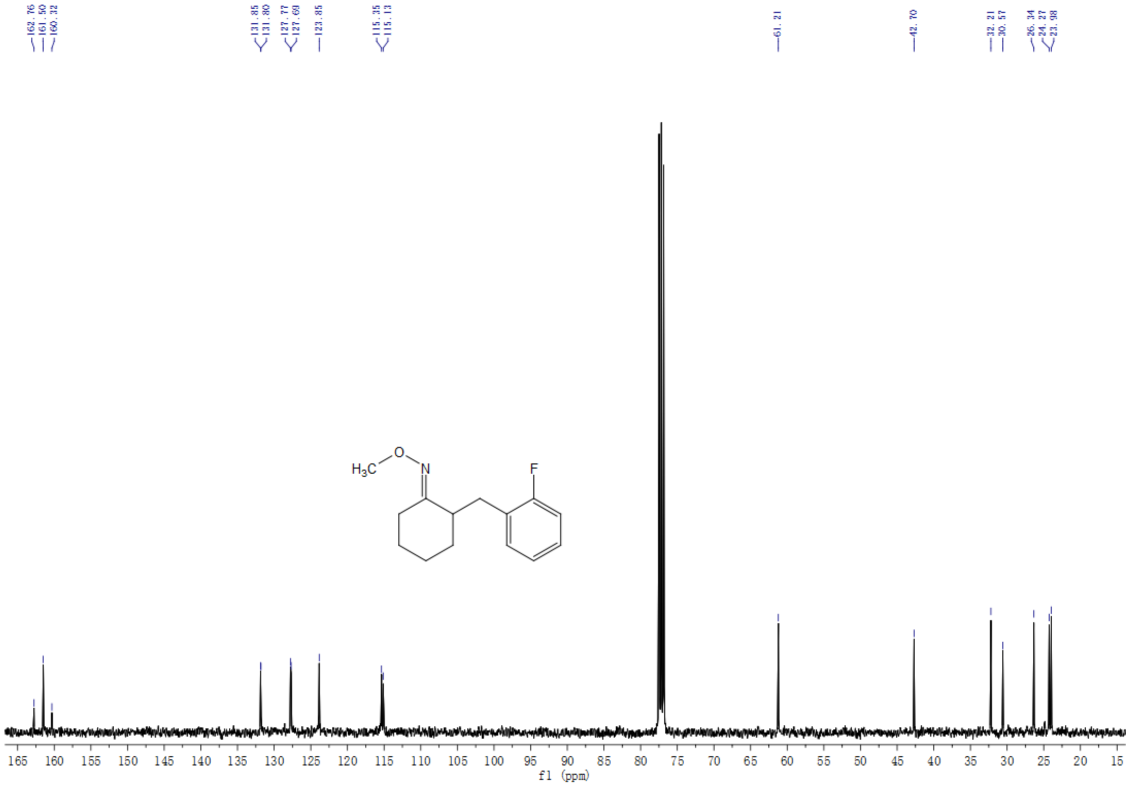

$^{1}$H NMR and $^{13}$C NMR spectra of compound **3aj**

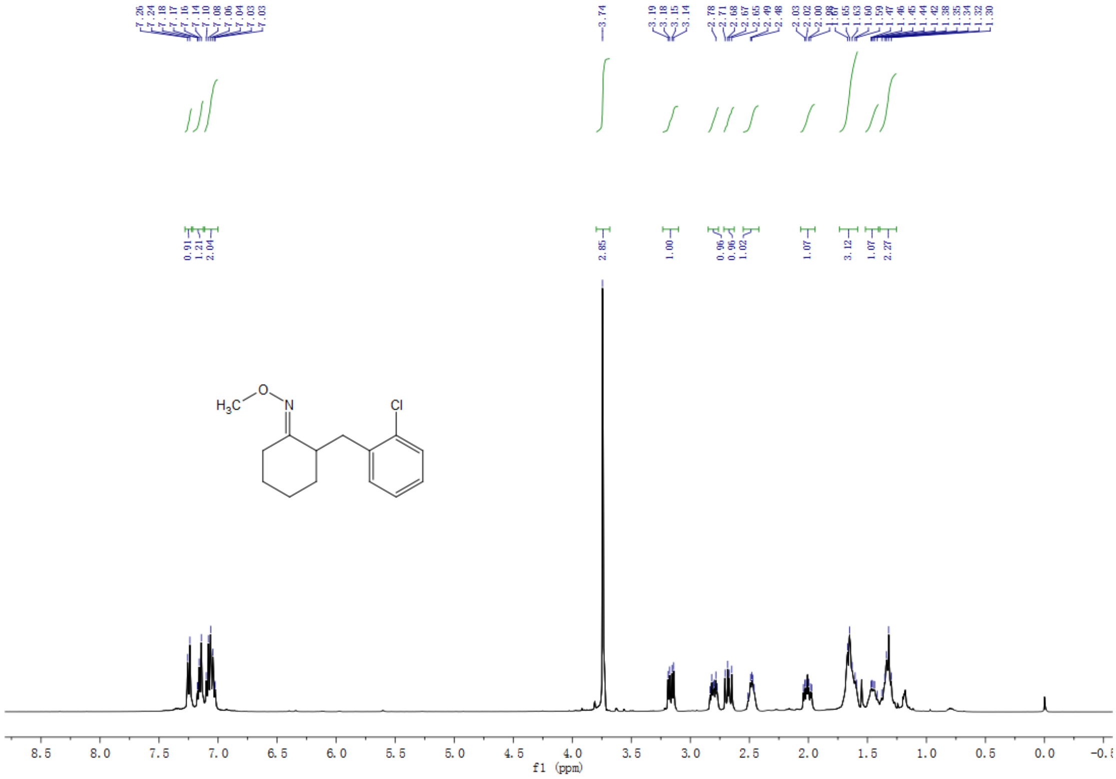

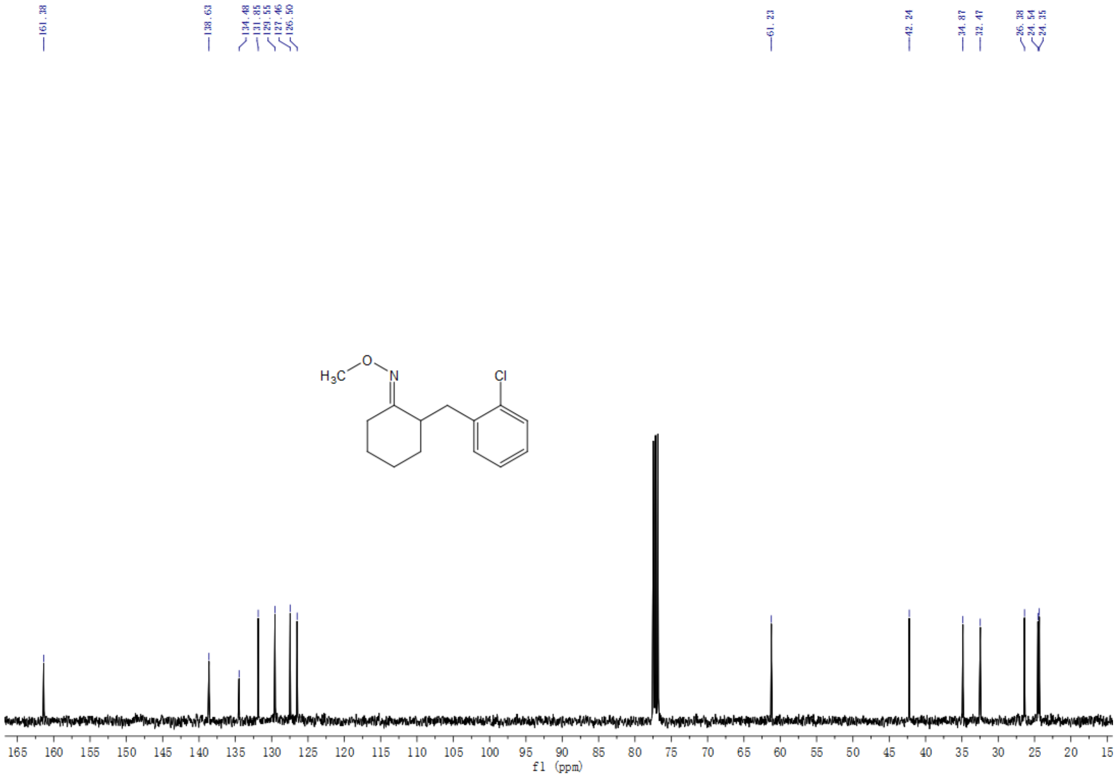

$^{1}H$ NMR and $^{13}C$ NMR spectra of compound **3ak**

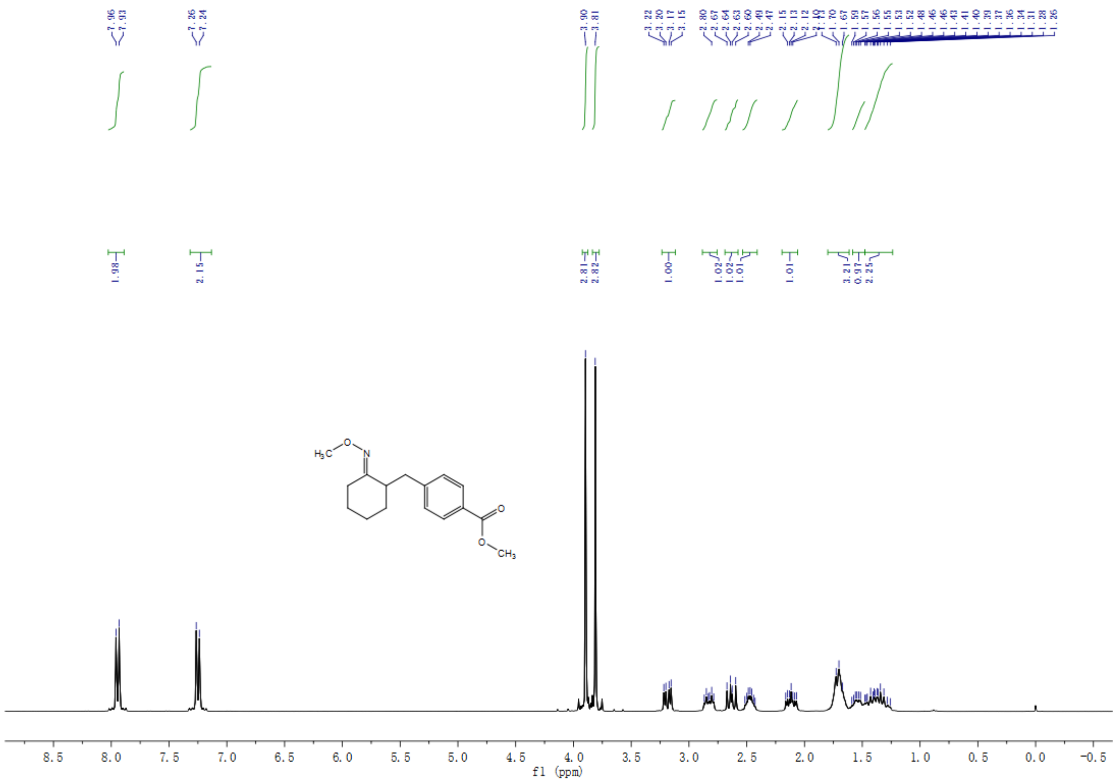

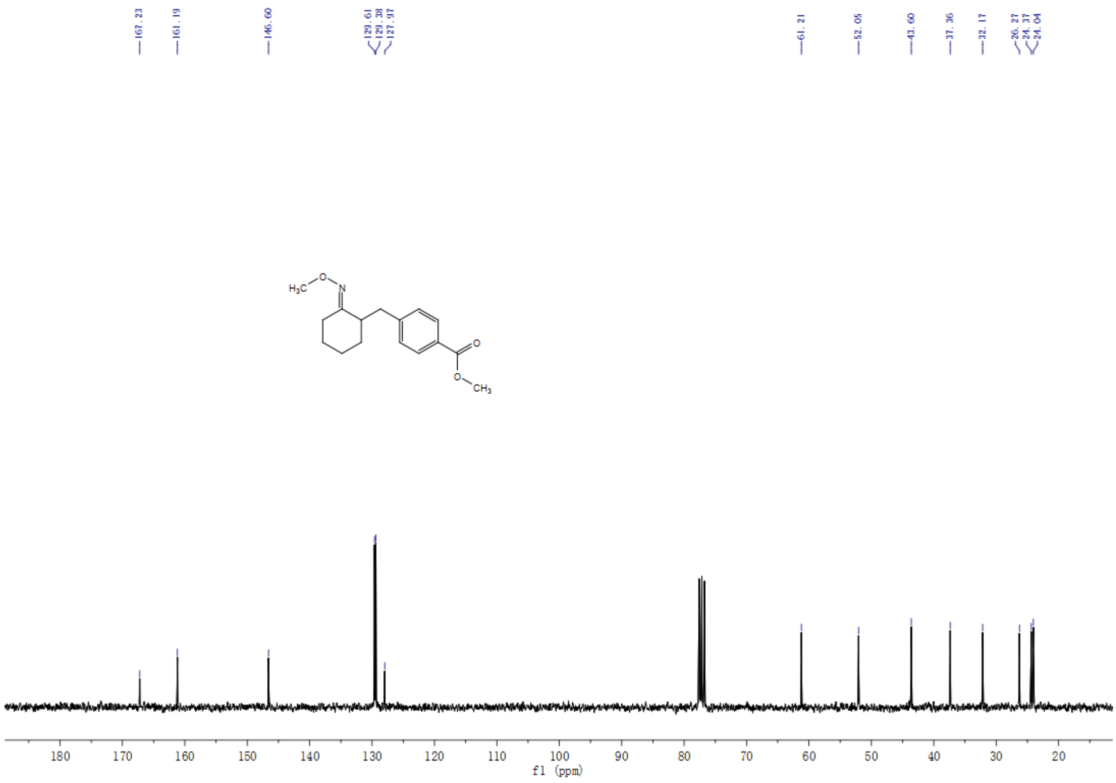

$^{1}$H NMR and $^{13}$C NMR spectra of compound **3ag**

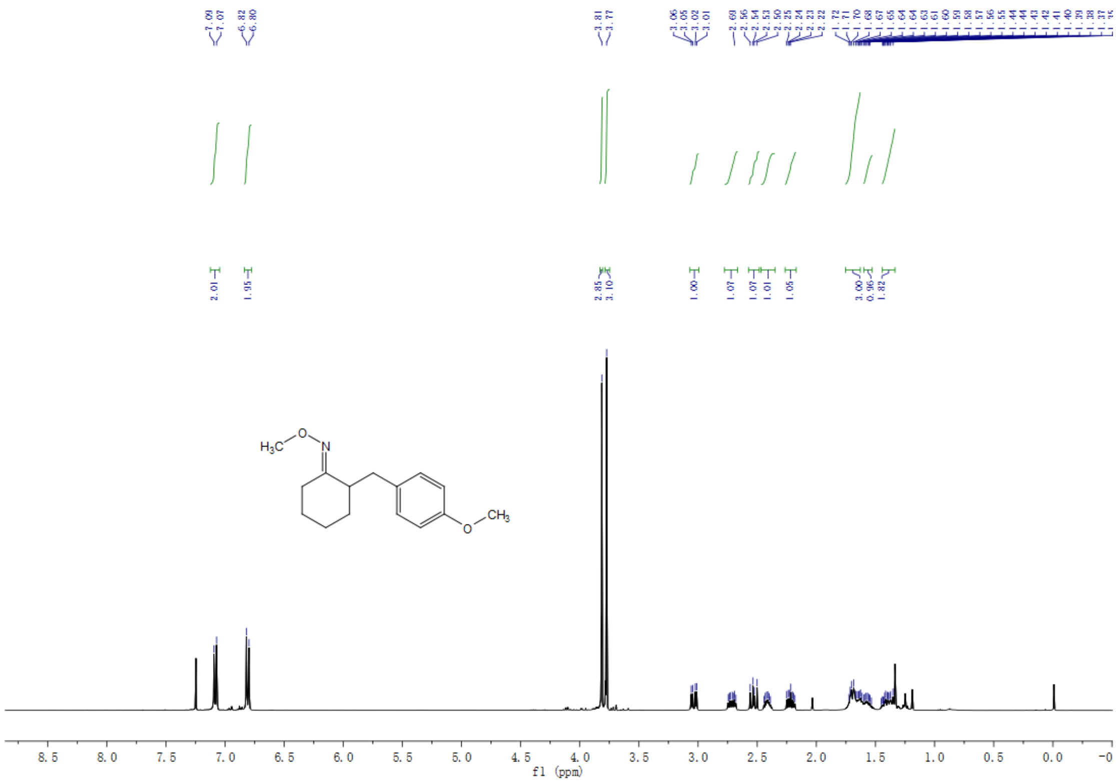

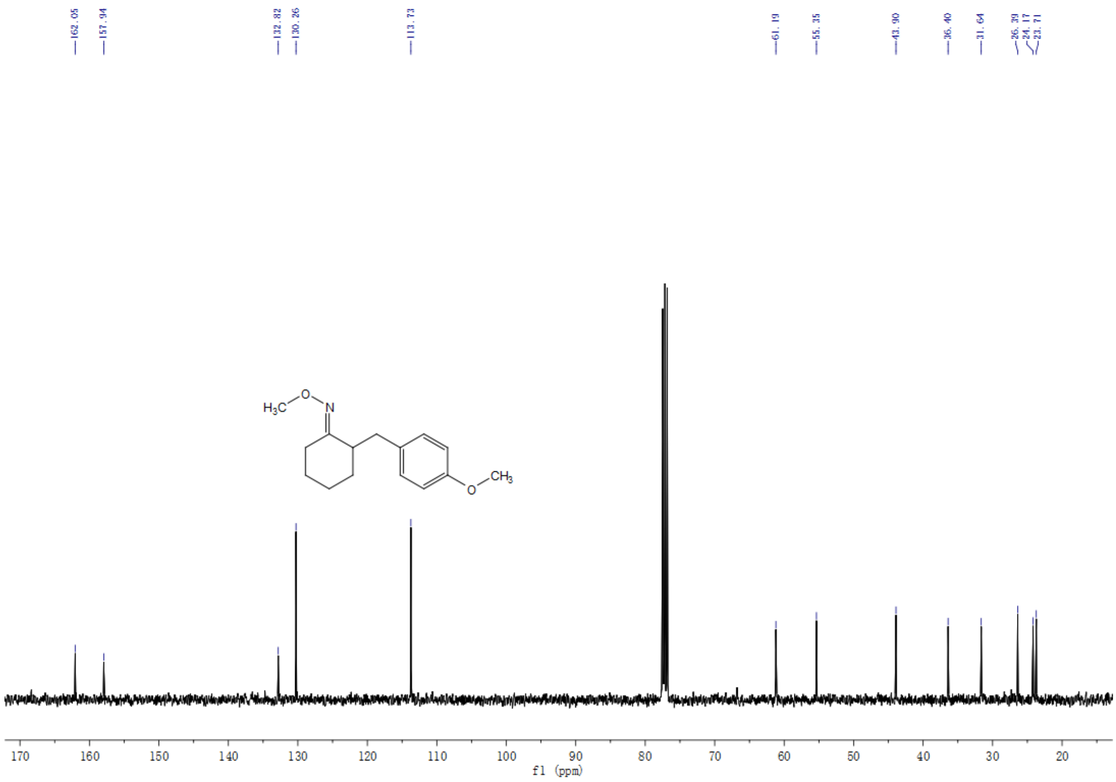

$^{1}$H NMR and $^{13}$C NMR spectra of compound **3ai**

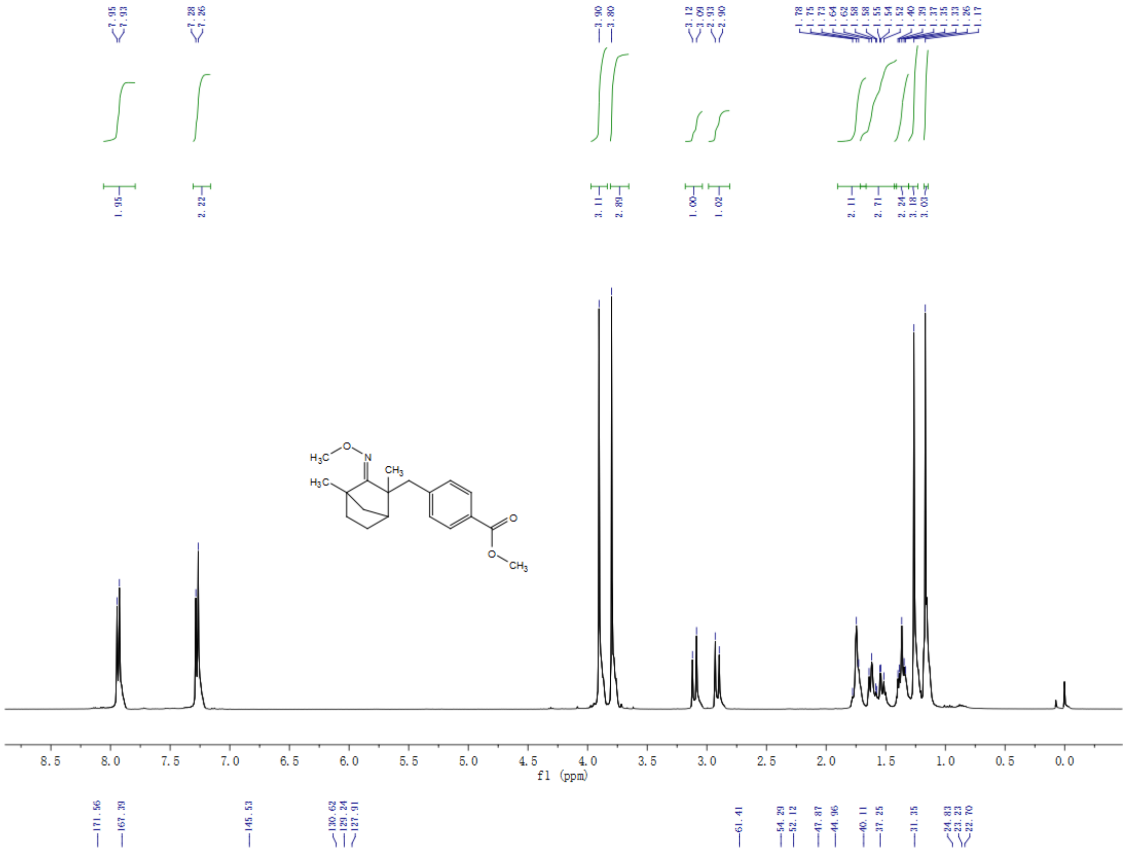

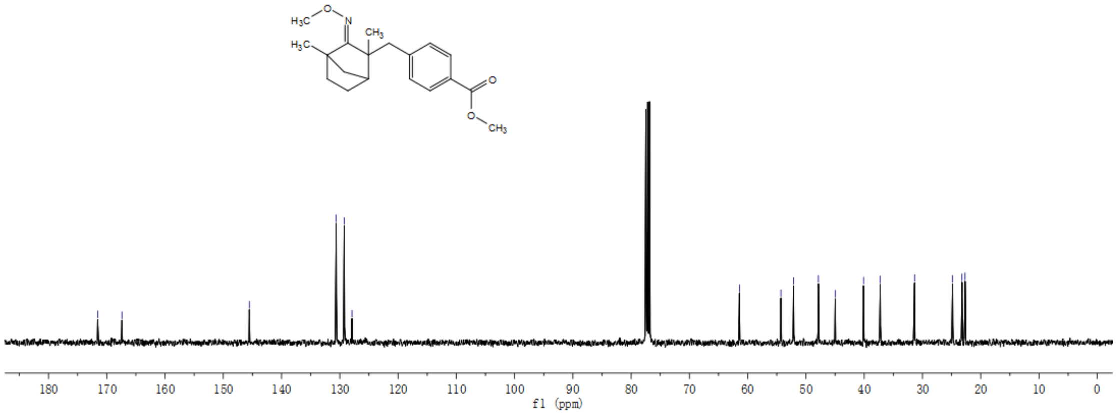

$^1$H NMR and $^{13}$C NMR spectra of compound **3ig**

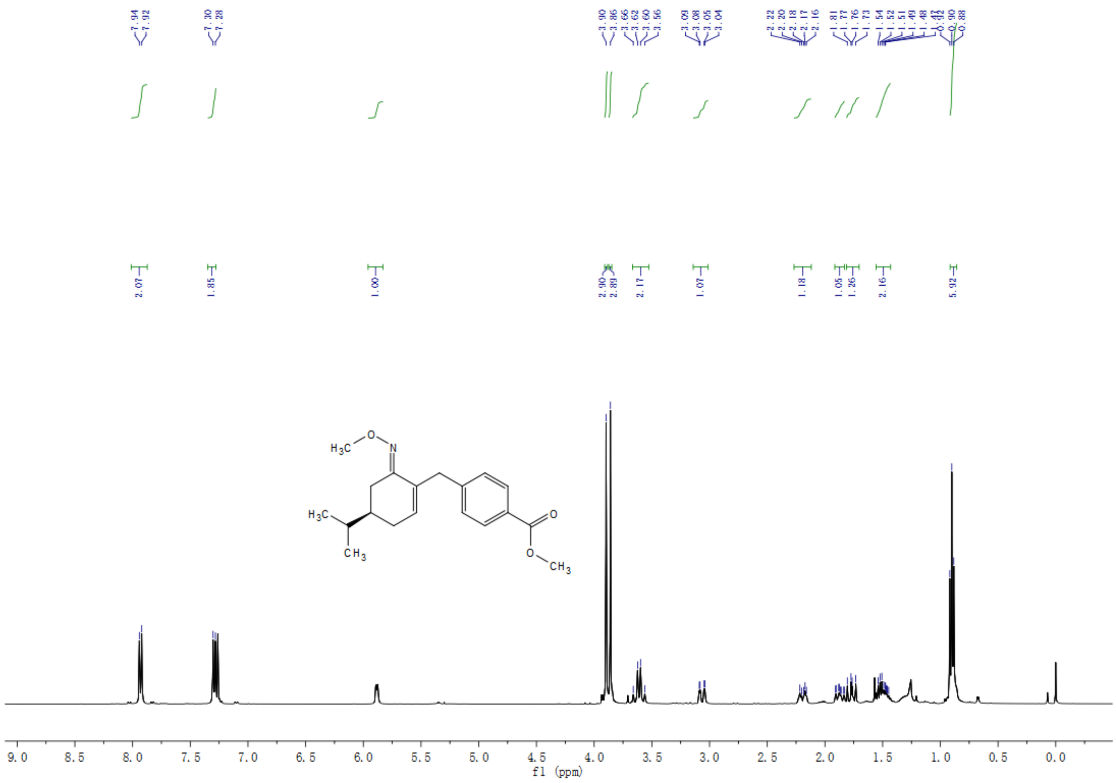

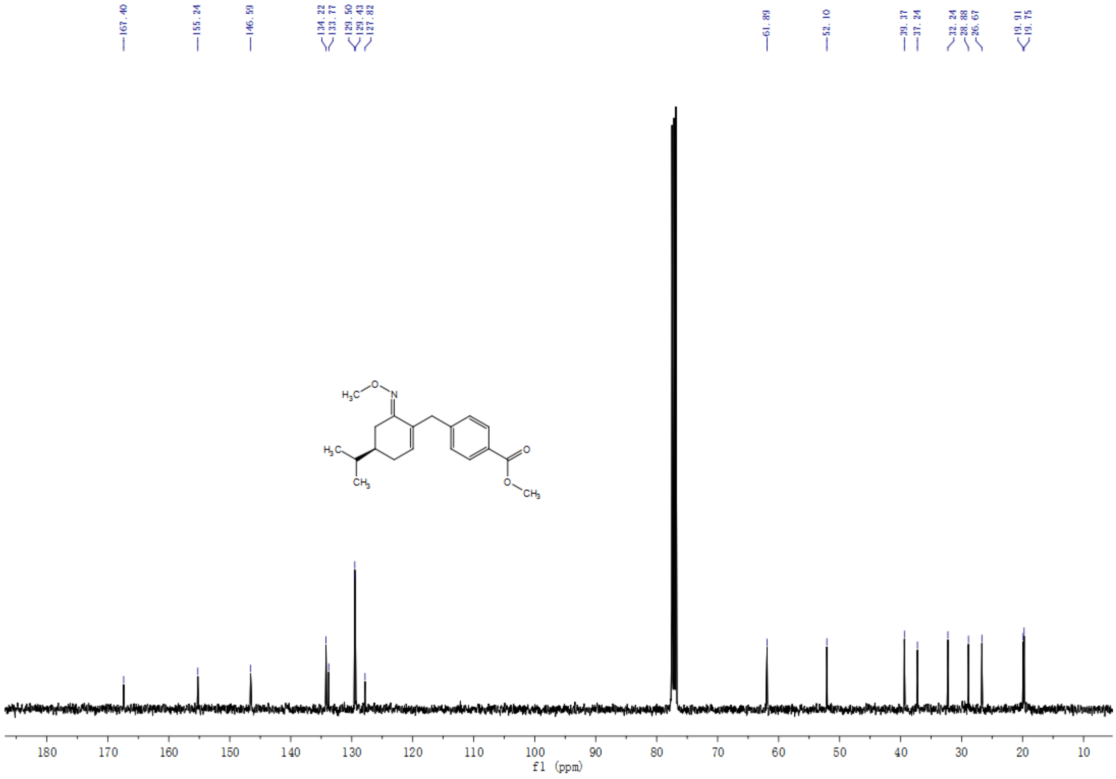

$^{1}H$ NMR and $^{13}C$ NMR spectra of compound **3jg**

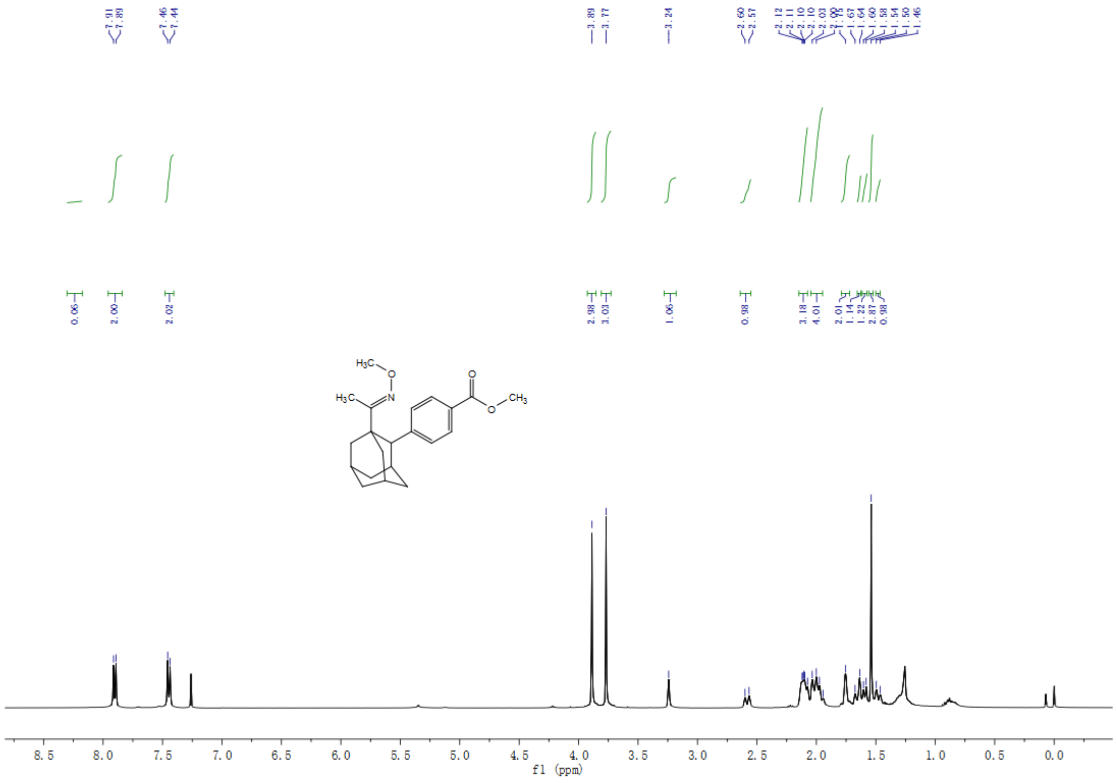


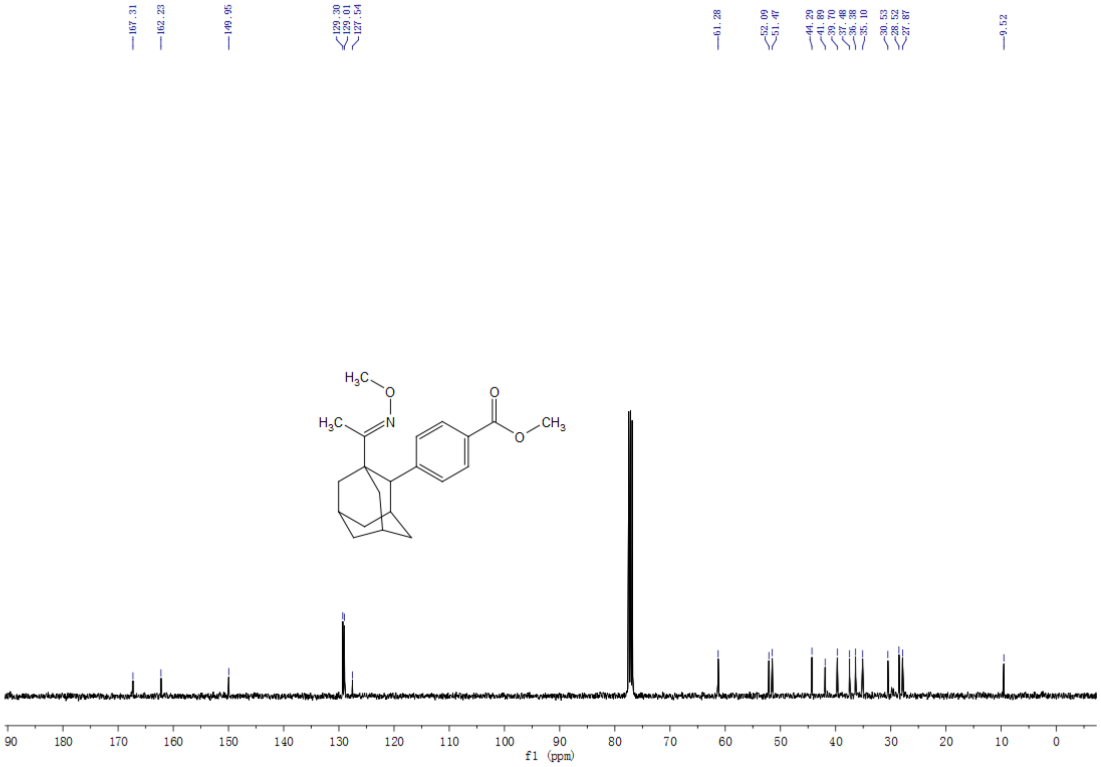


$^{1}H$ NMR and $^{13}C$ NMR spectra of compound **3hg**

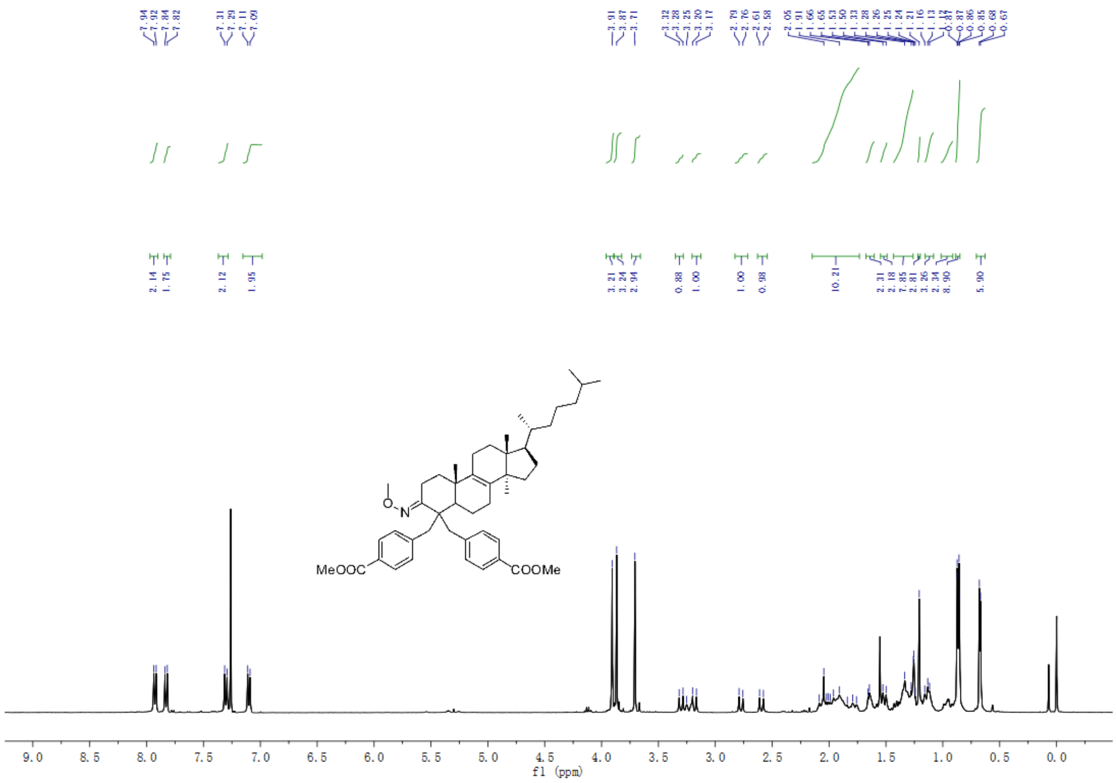

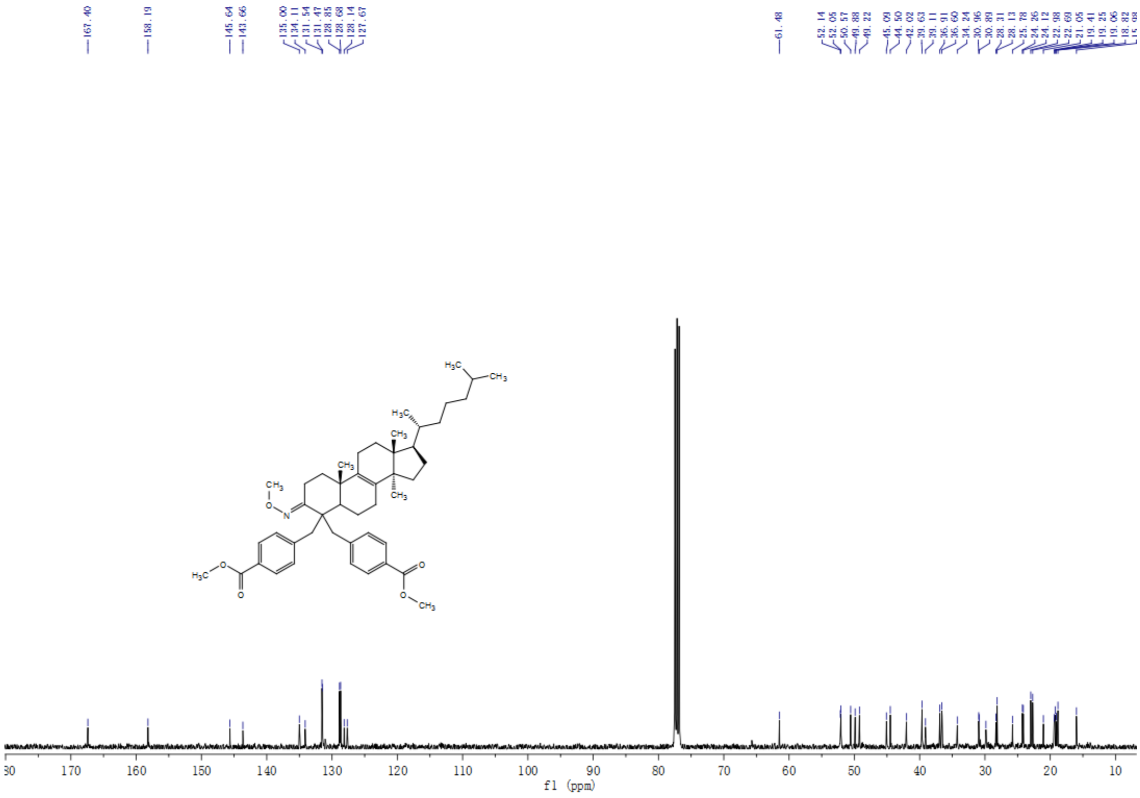

$^{1}$H NMR and $^{13}$C NMR spectra of compound **3kg**

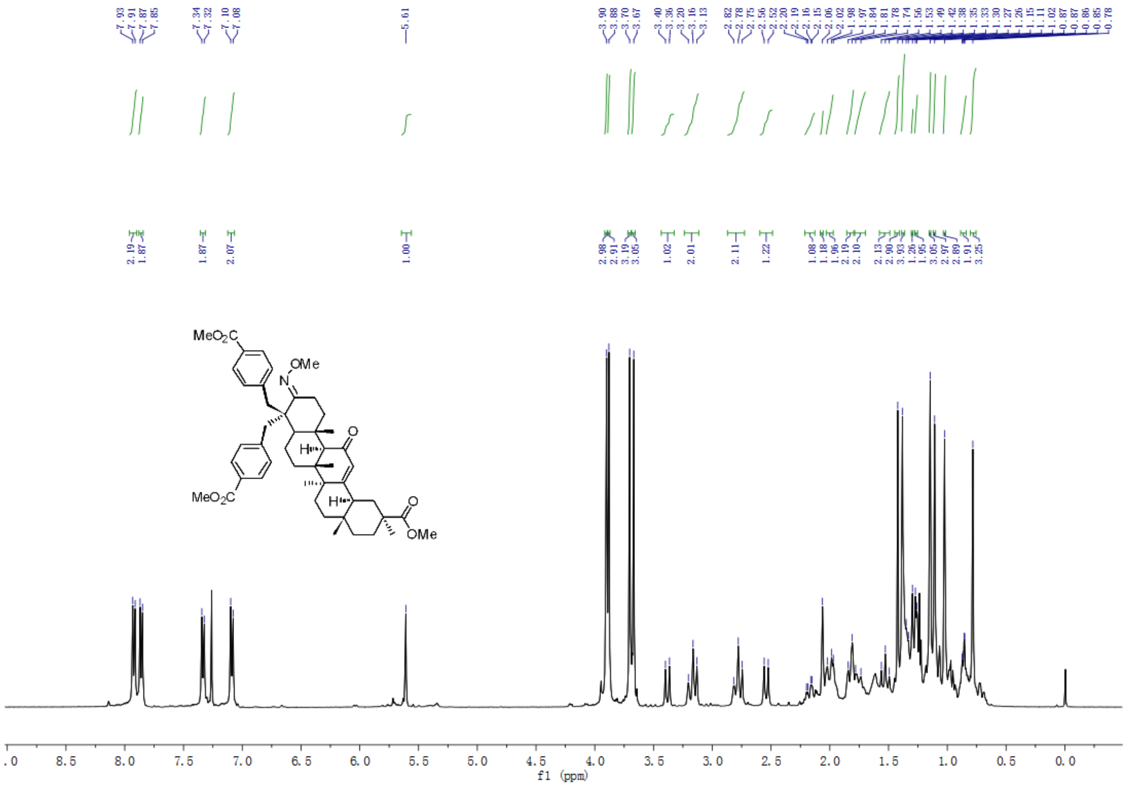

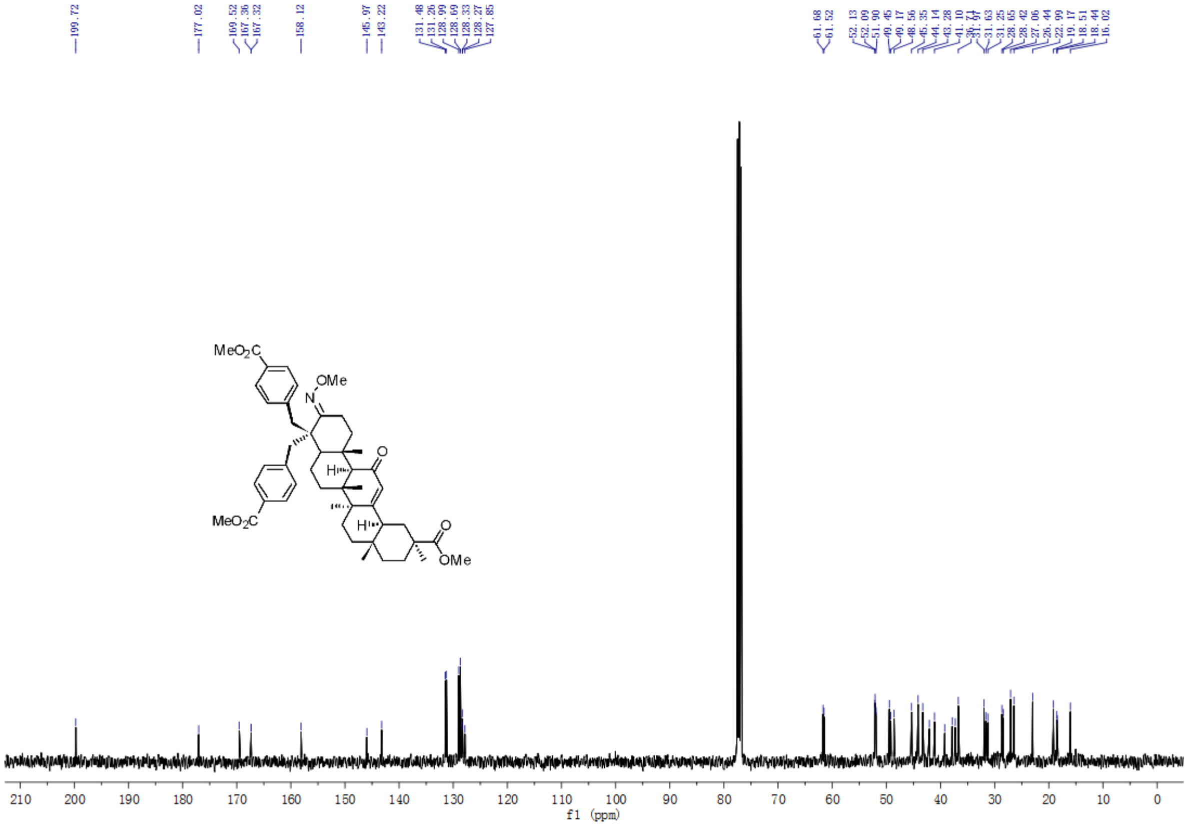

$^{1}H$ NMR and $^{13}C$ NMR spectra of compound **3jg**

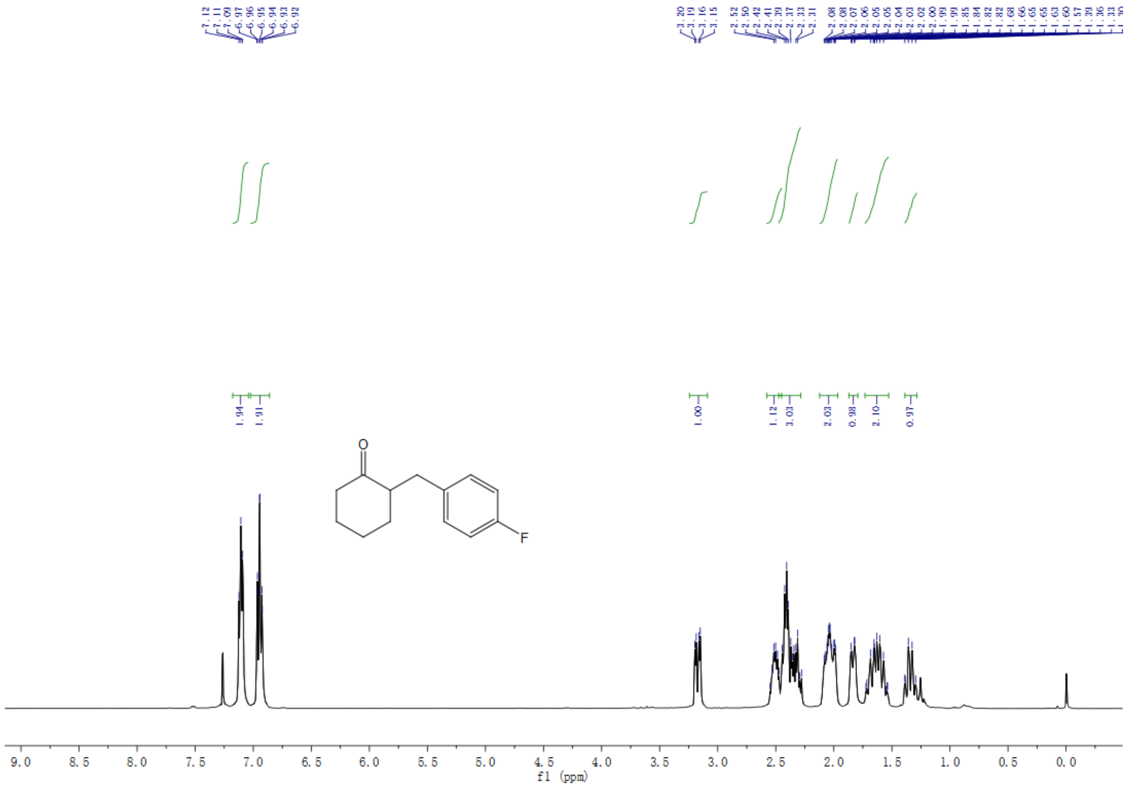

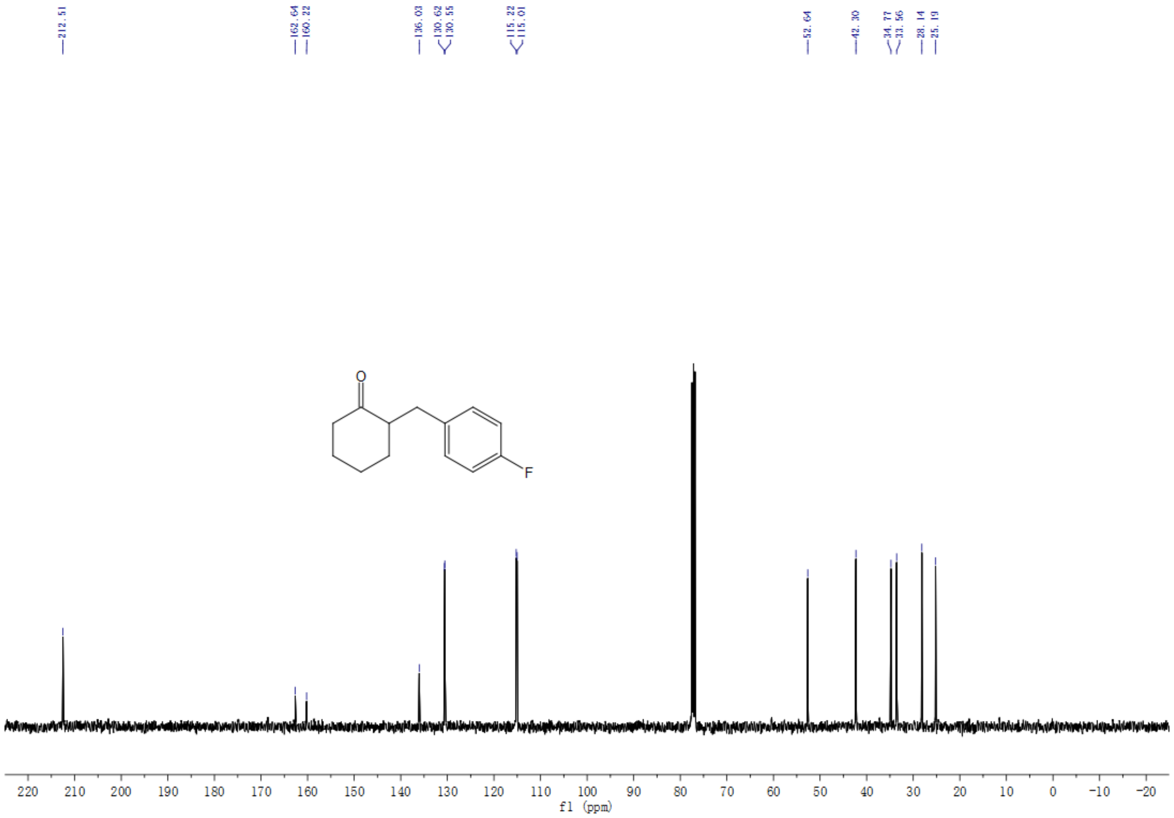

$^{1}$H NMR and $^{13}$C NMR spectra of compound **4ab**

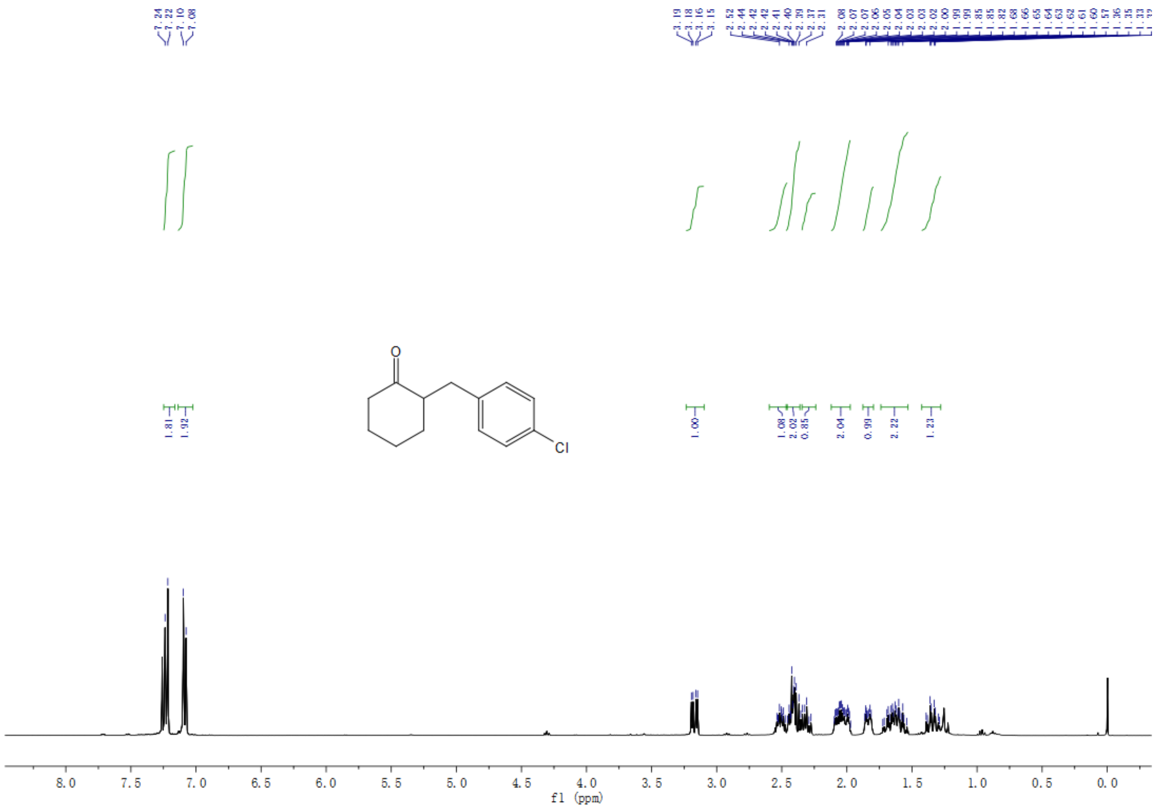


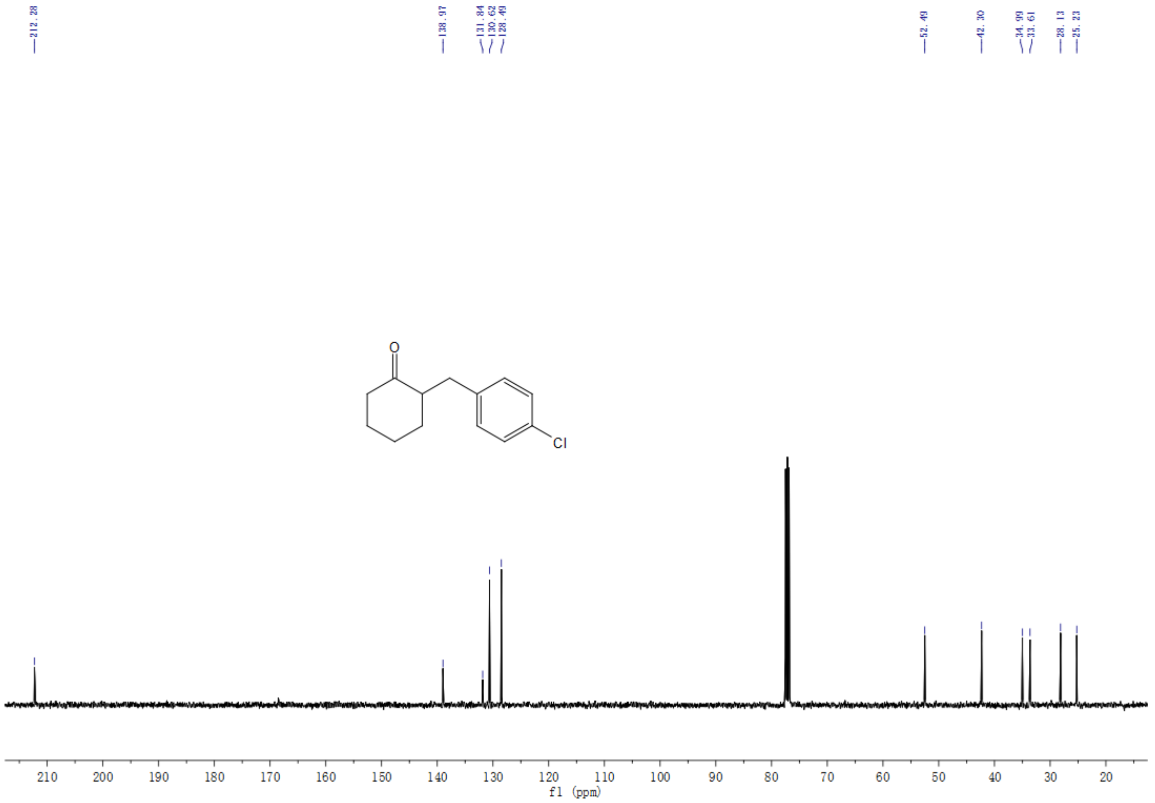


$^{1}$H NMR and $^{13}$C NMR spectra of compound **4ac**

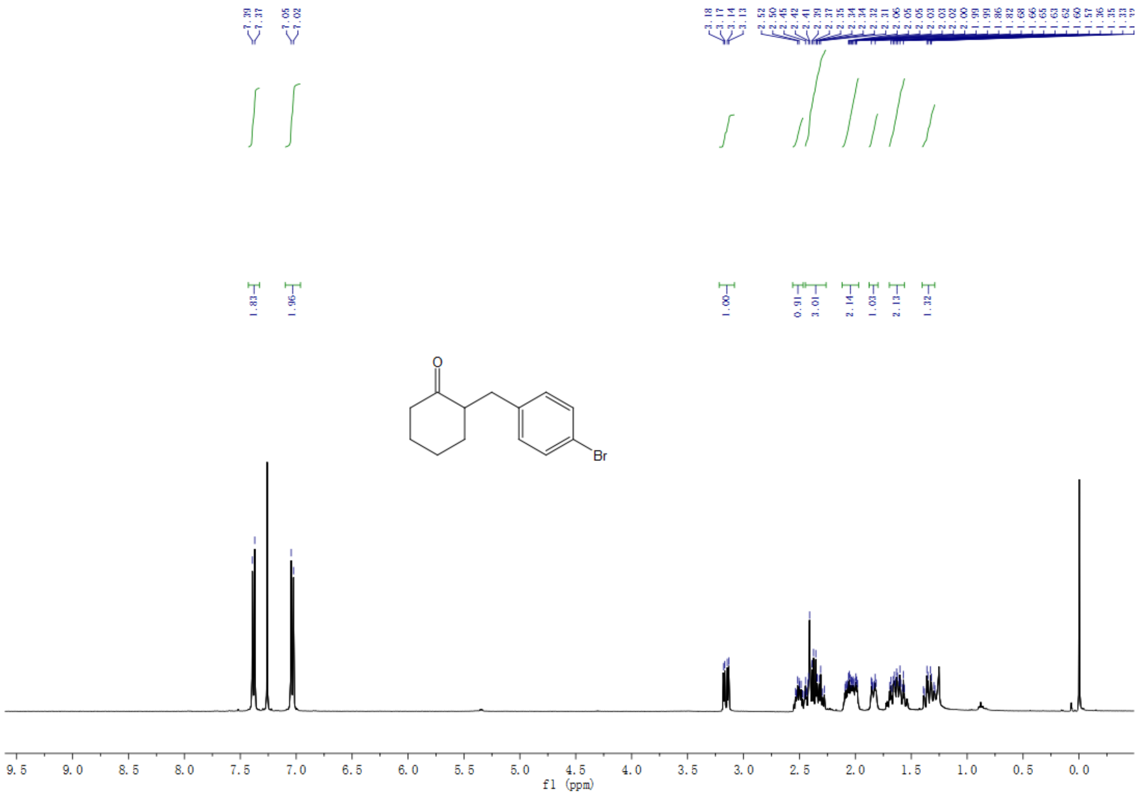


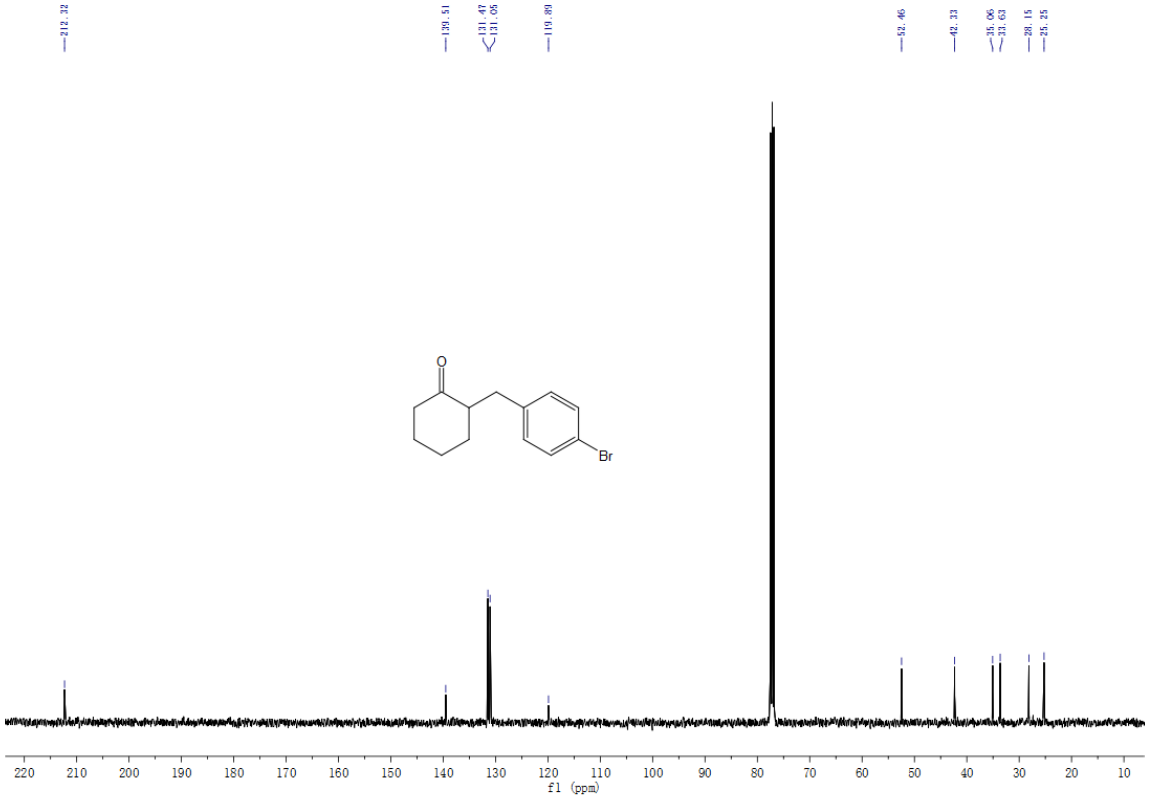


$^{1}$H NMR and $^{13}$C NMR spectra of compound **4ad**

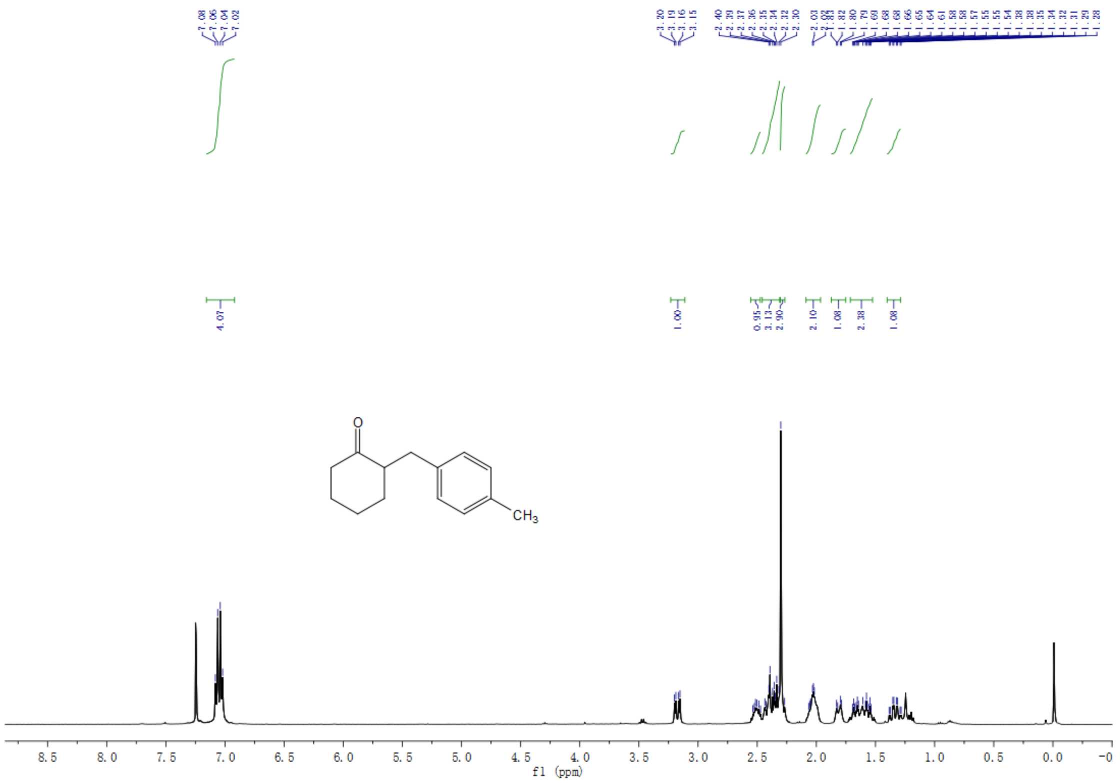

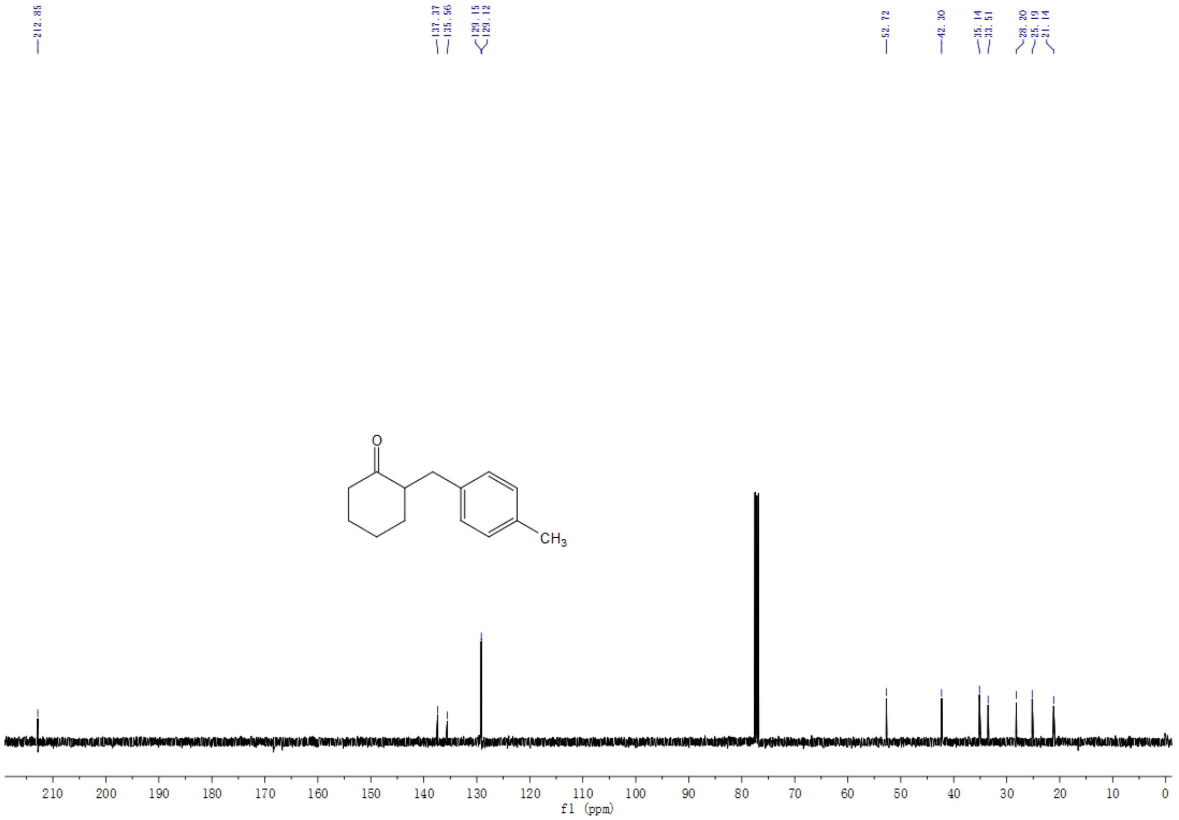

$^{1}$H NMR and $^{13}$C NMR spectra of compound **4ae**

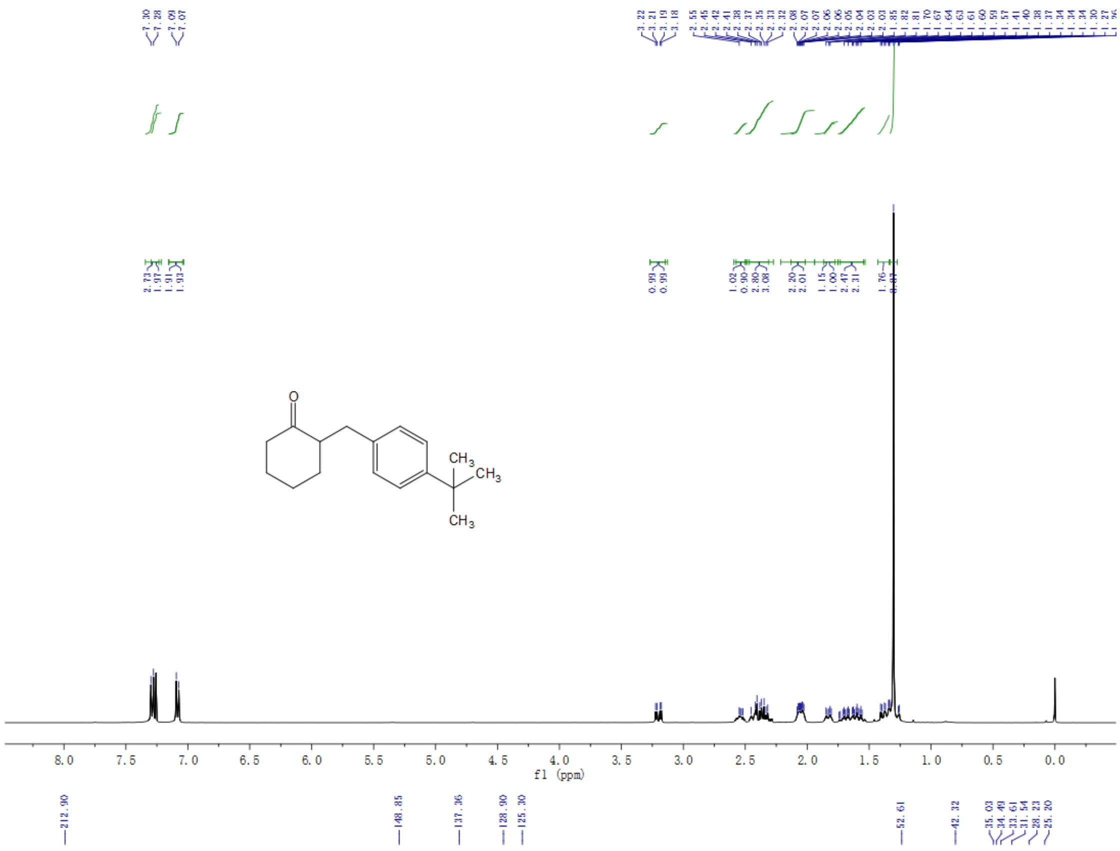

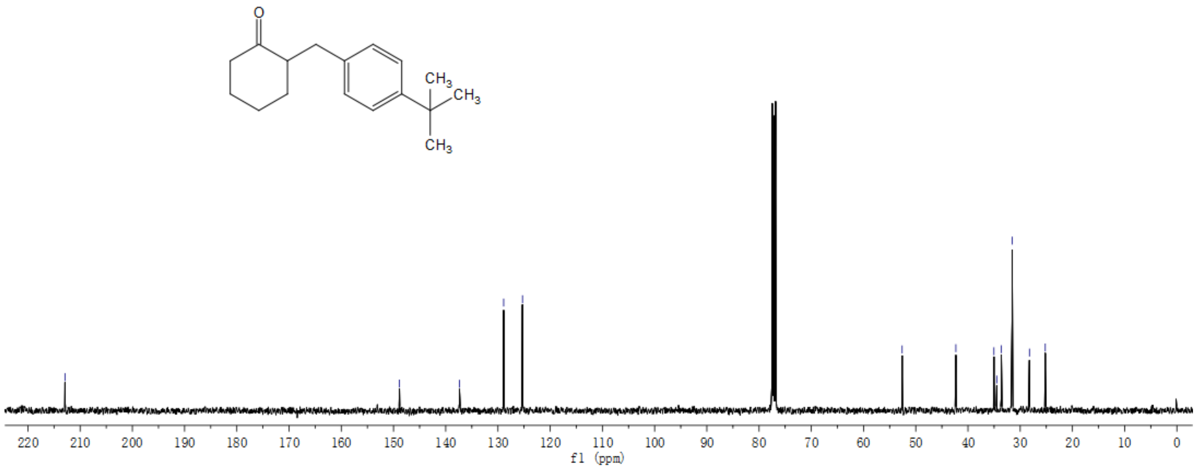

$^{1}H$ NMR and $^{13}C$ NMR spectra of compound **4af**

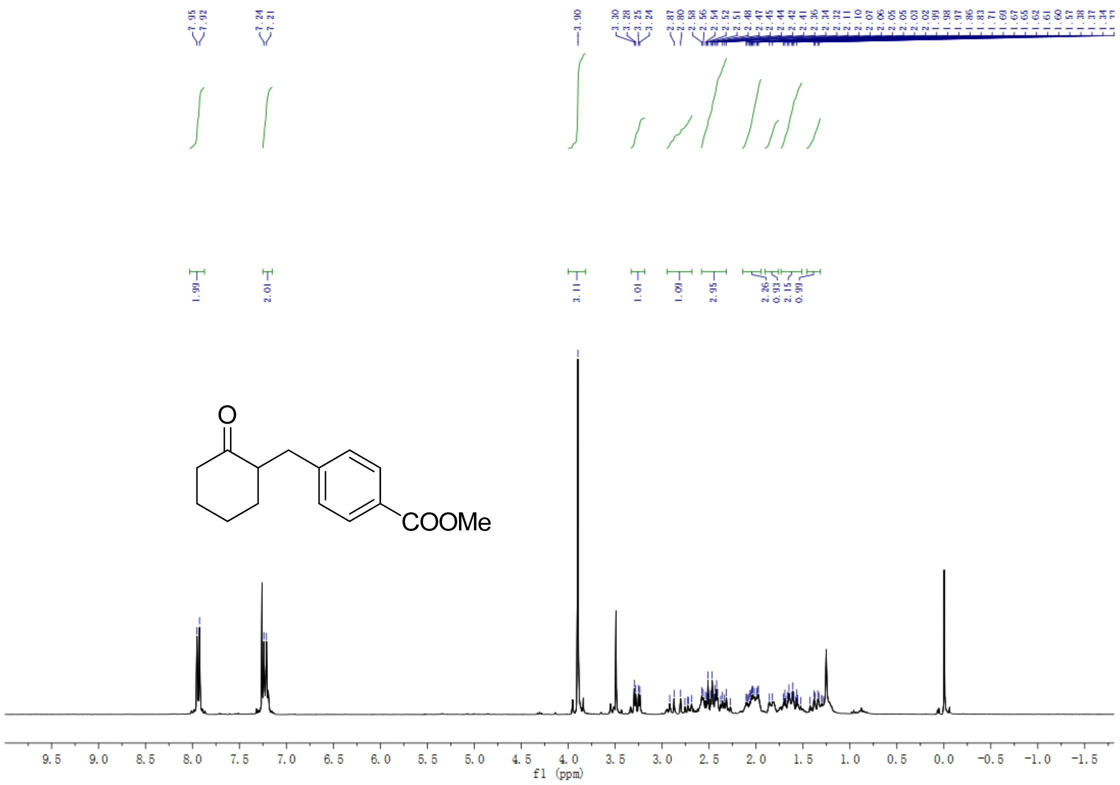

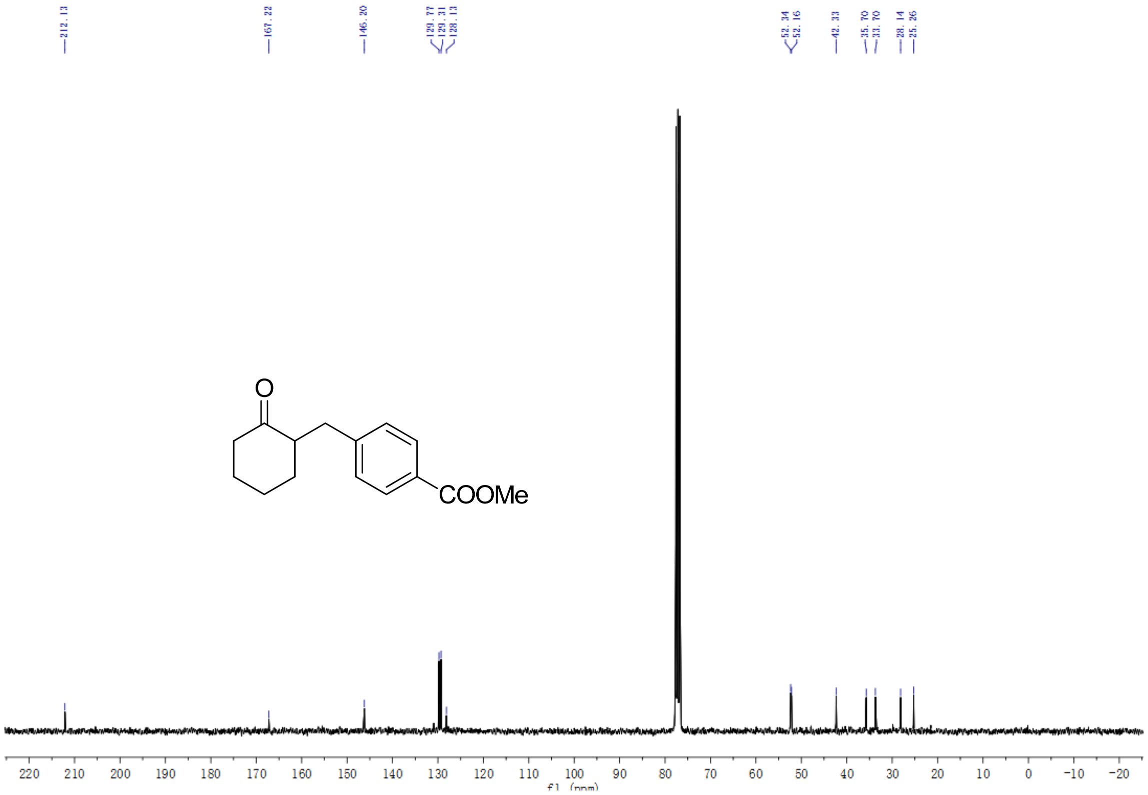

$^{1}H$ NMR and $^{13}C$ NMR spectra of compound **4ag**

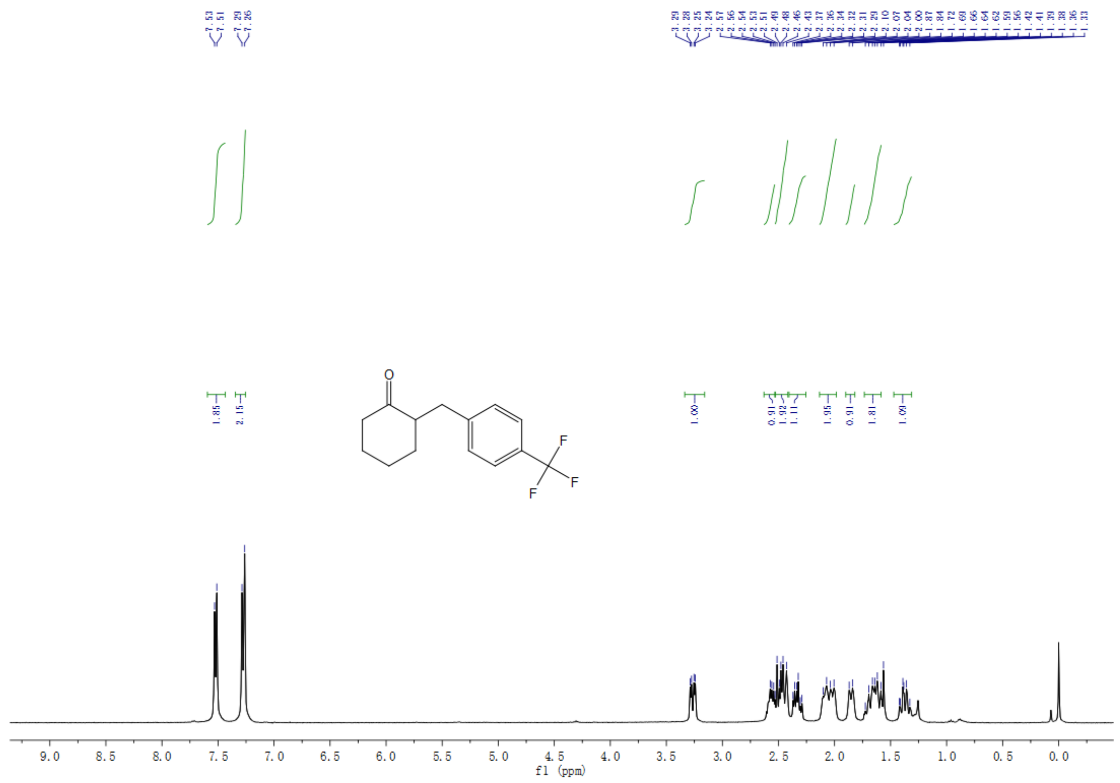


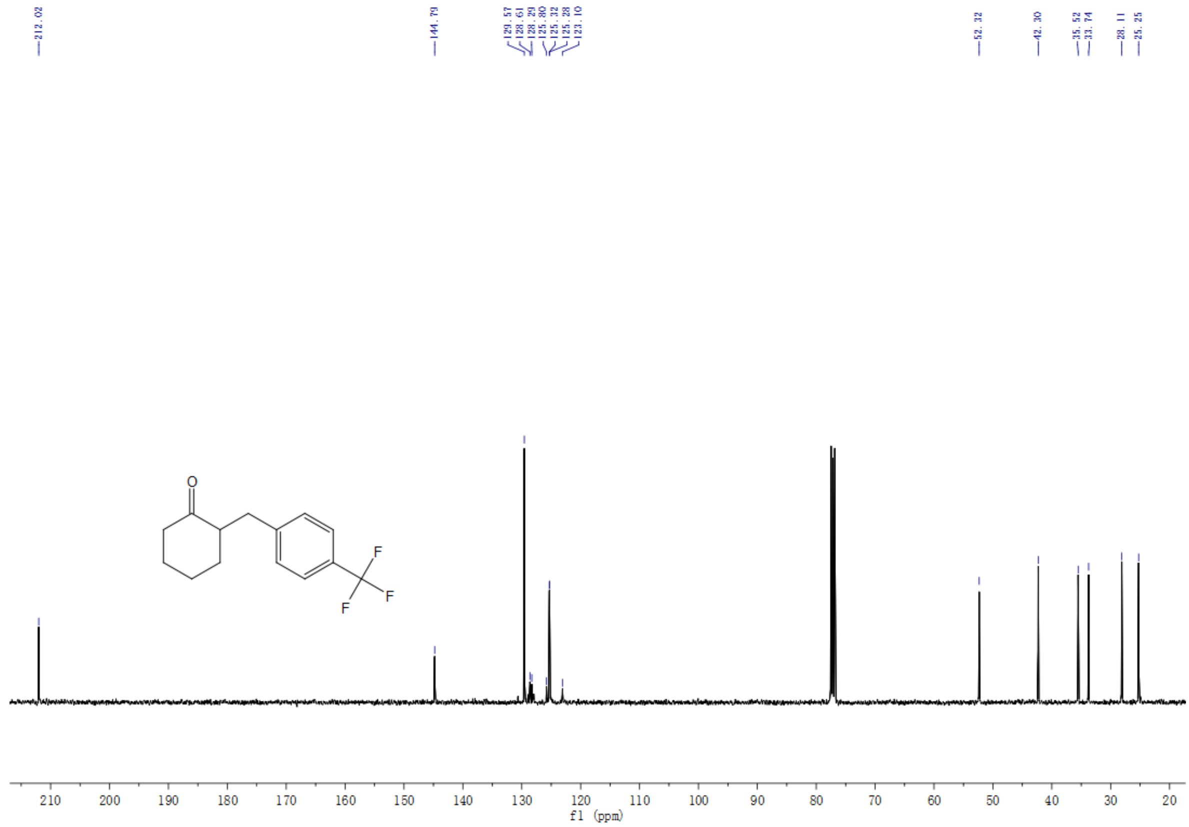


$^1$H NMR and $^{13}$C NMR spectra of compound **4ah**

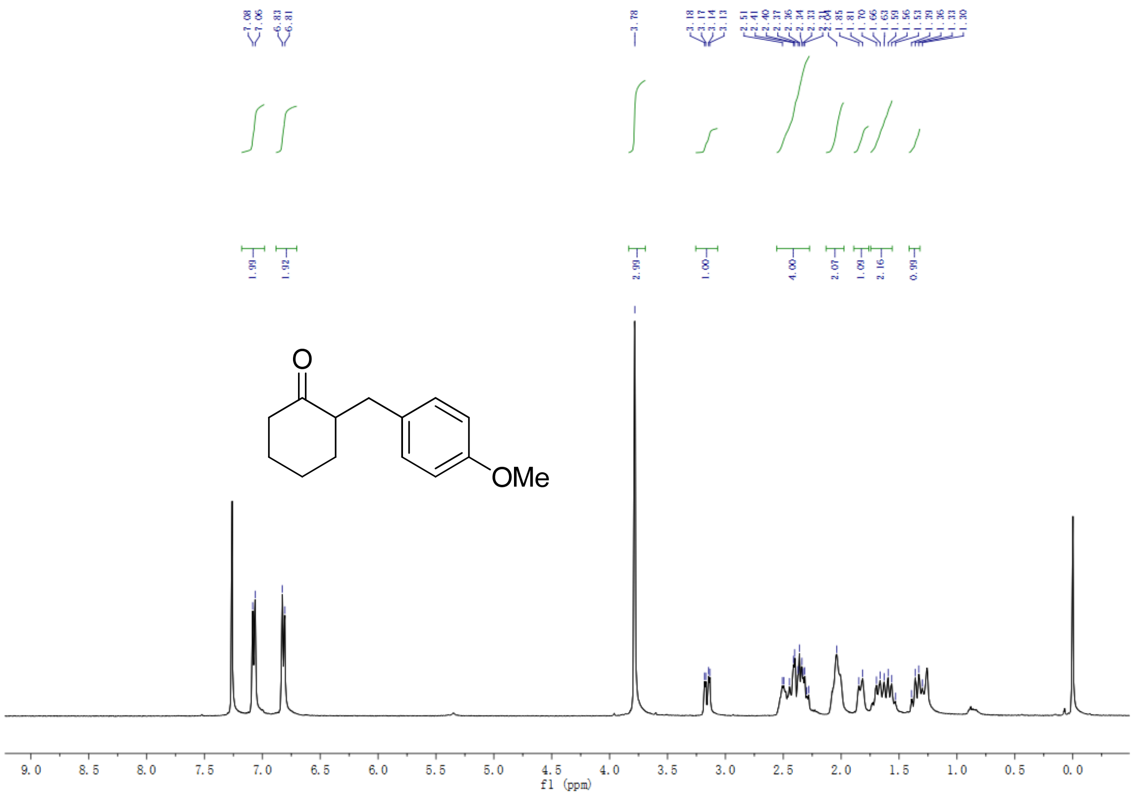


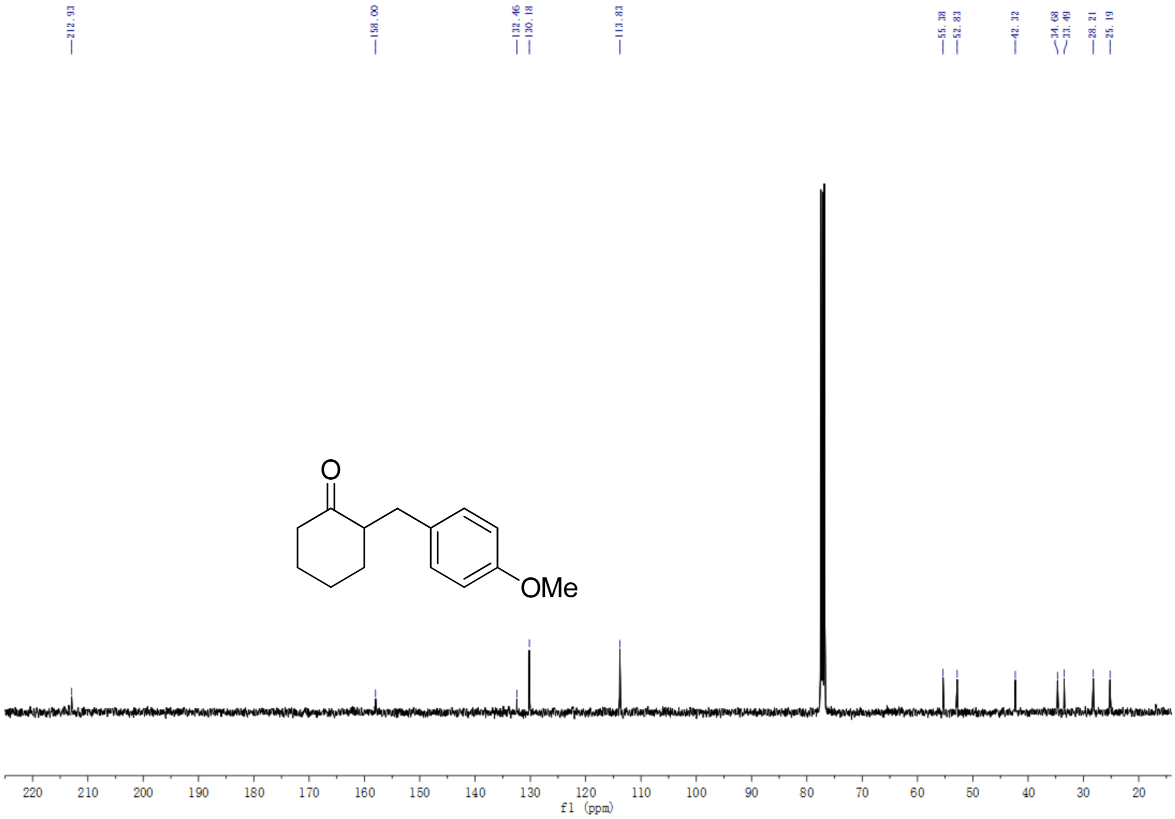


$^{1}$H NMR and $^{13}$C NMR spectra of compound **4ai**

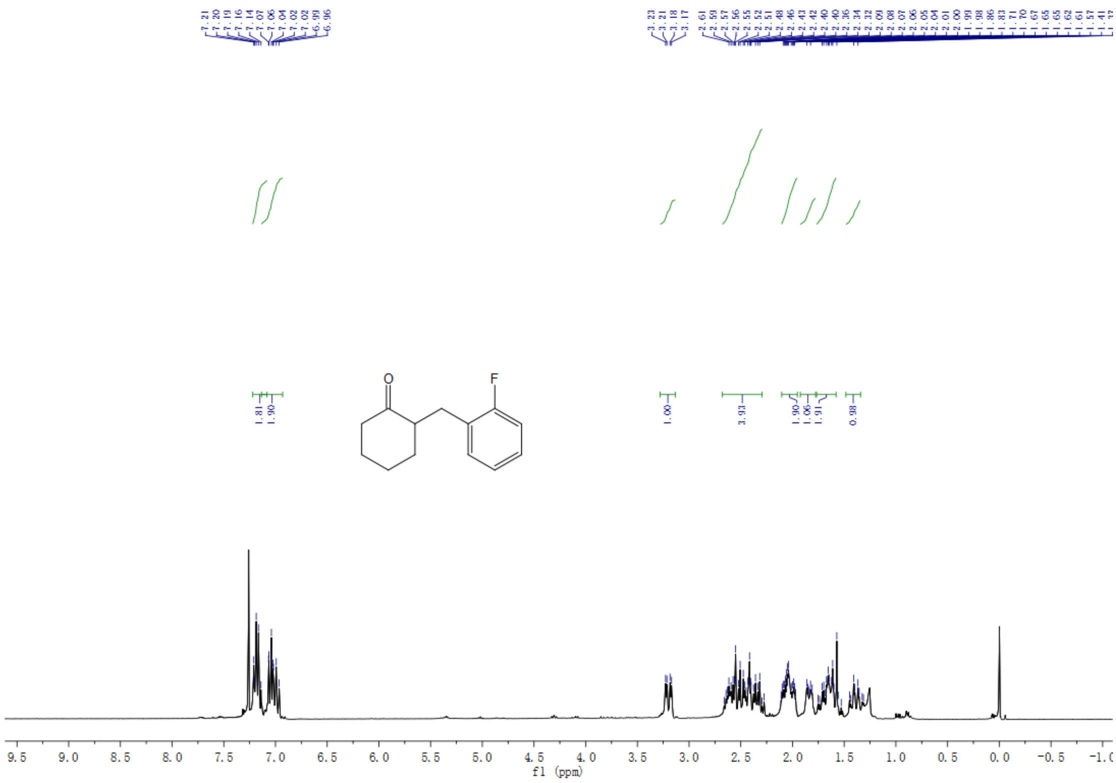

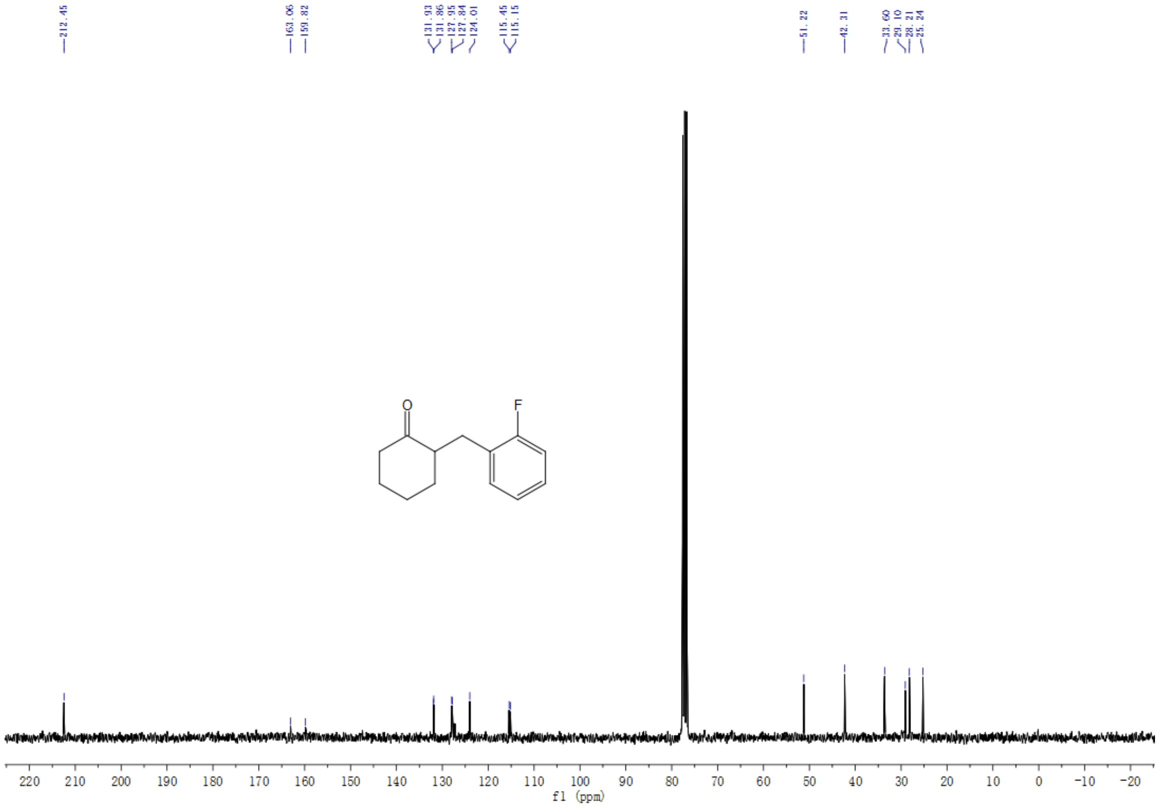

$^{1}$H NMR and $^{13}$C NMR spectra of compound **4aj**

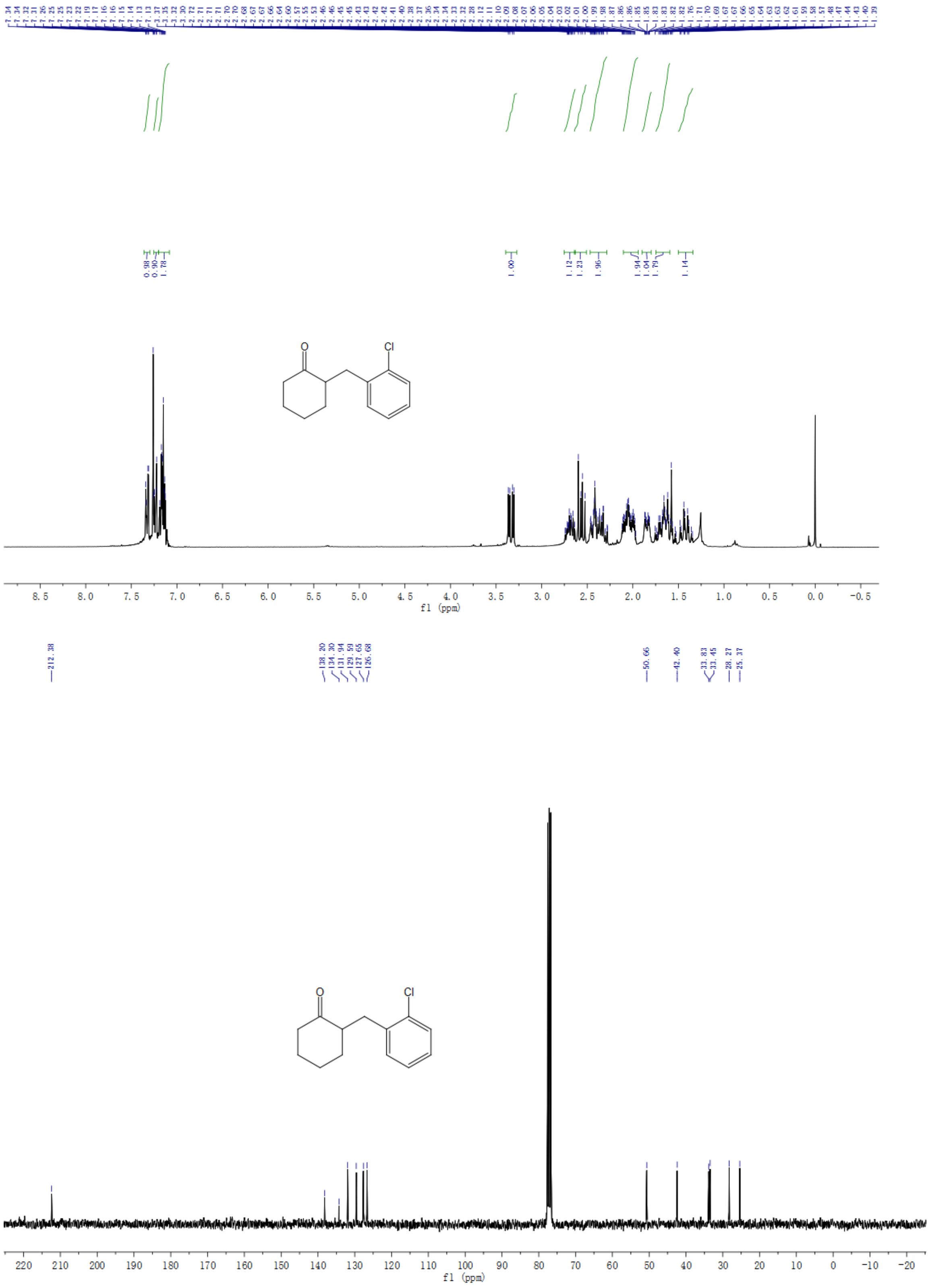

$^{1}$H NMR and $^{13}$C NMR spectra of compound **4ak**

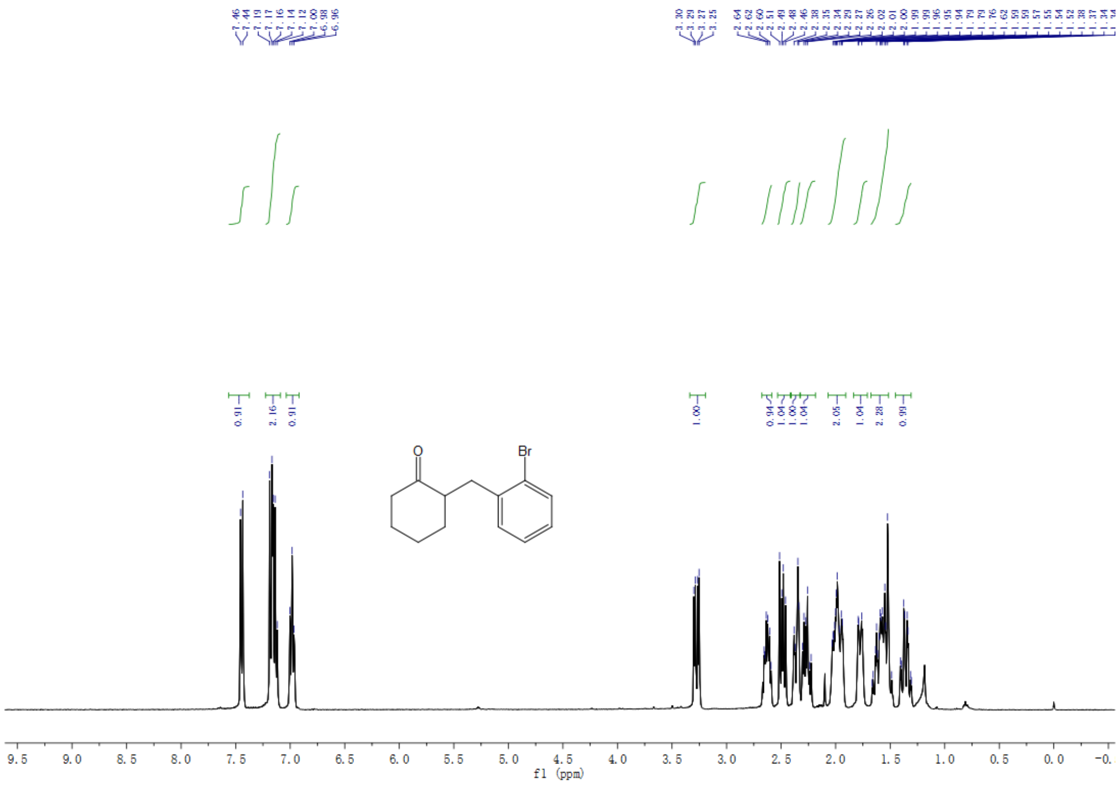

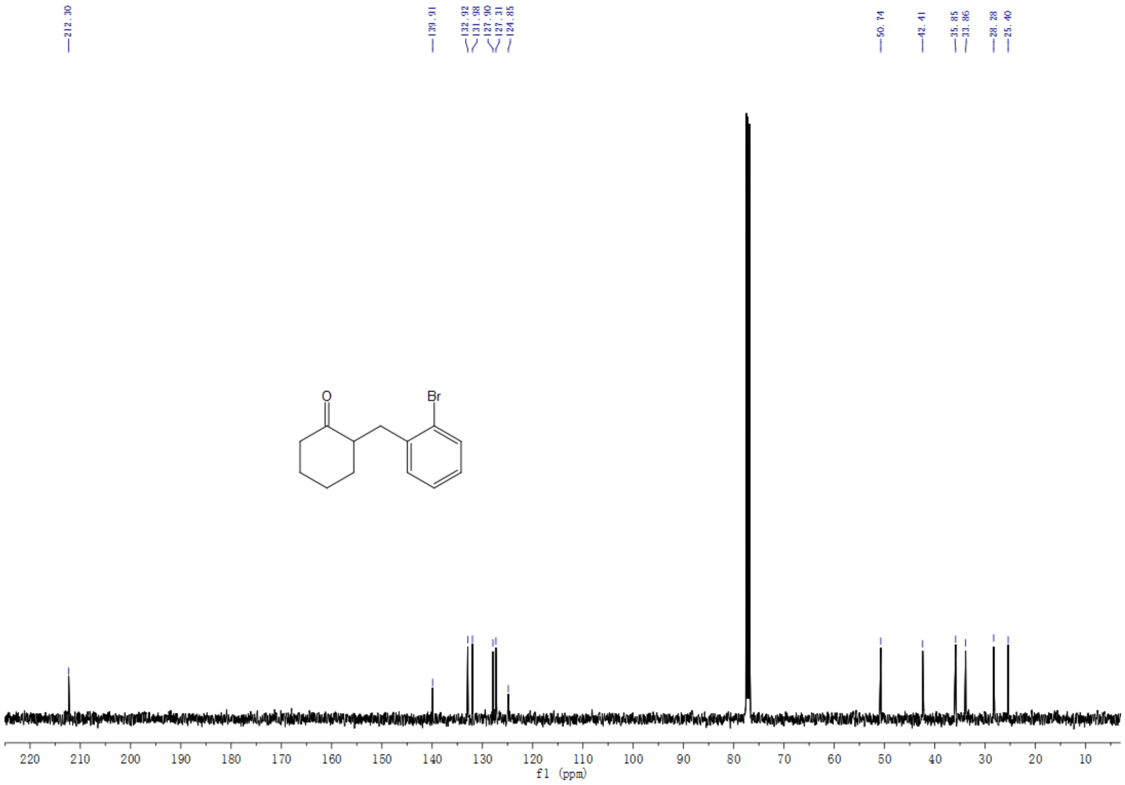

$^{1}$H NMR and $^{13}$C NMR spectra of compound **4al**

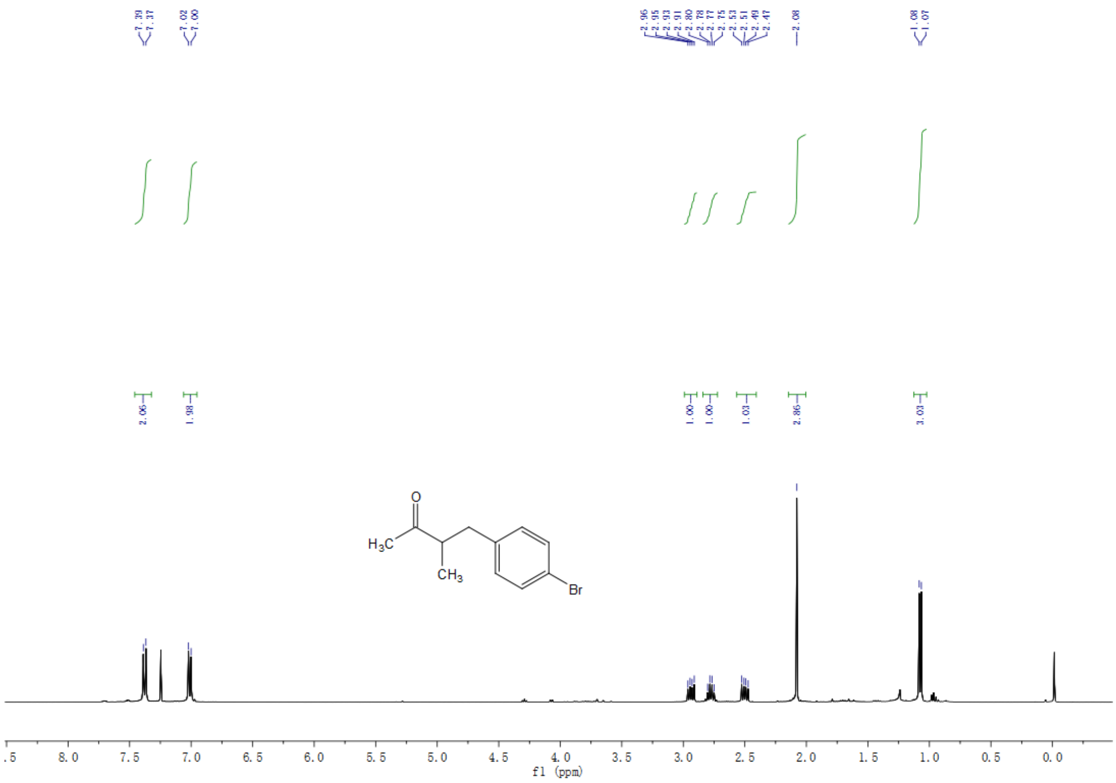


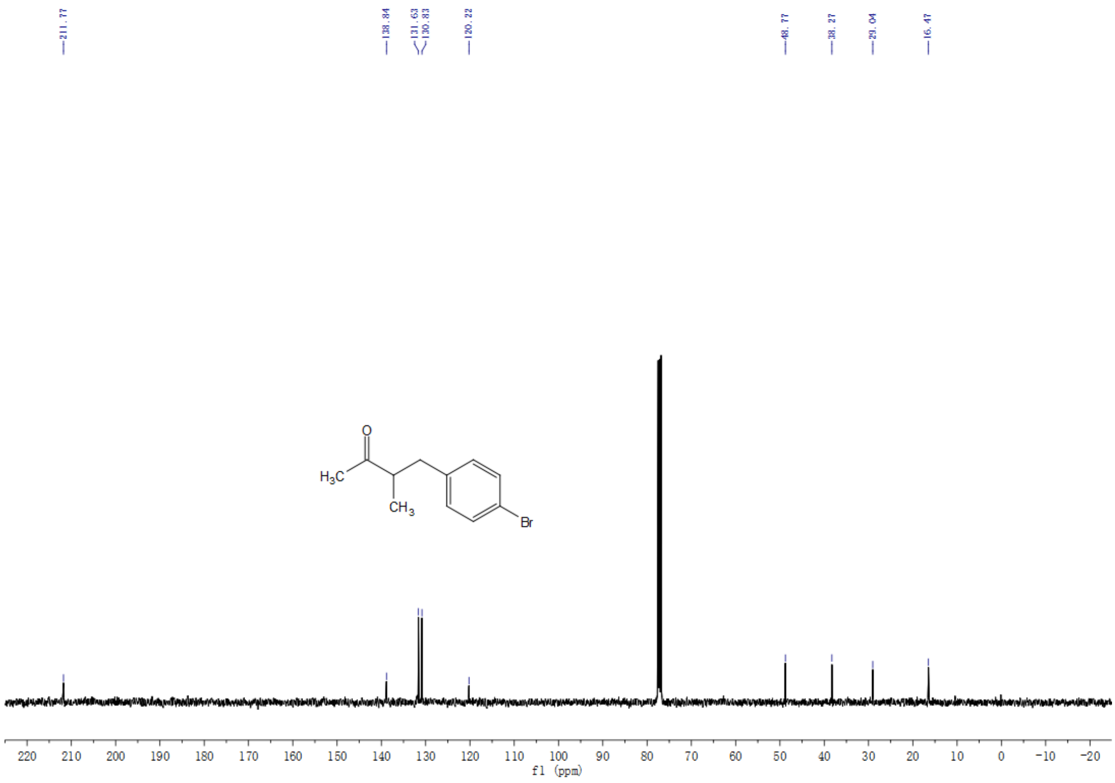


$^{1}$H NMR and $^{13}$C NMR spectra of compound **4bd**

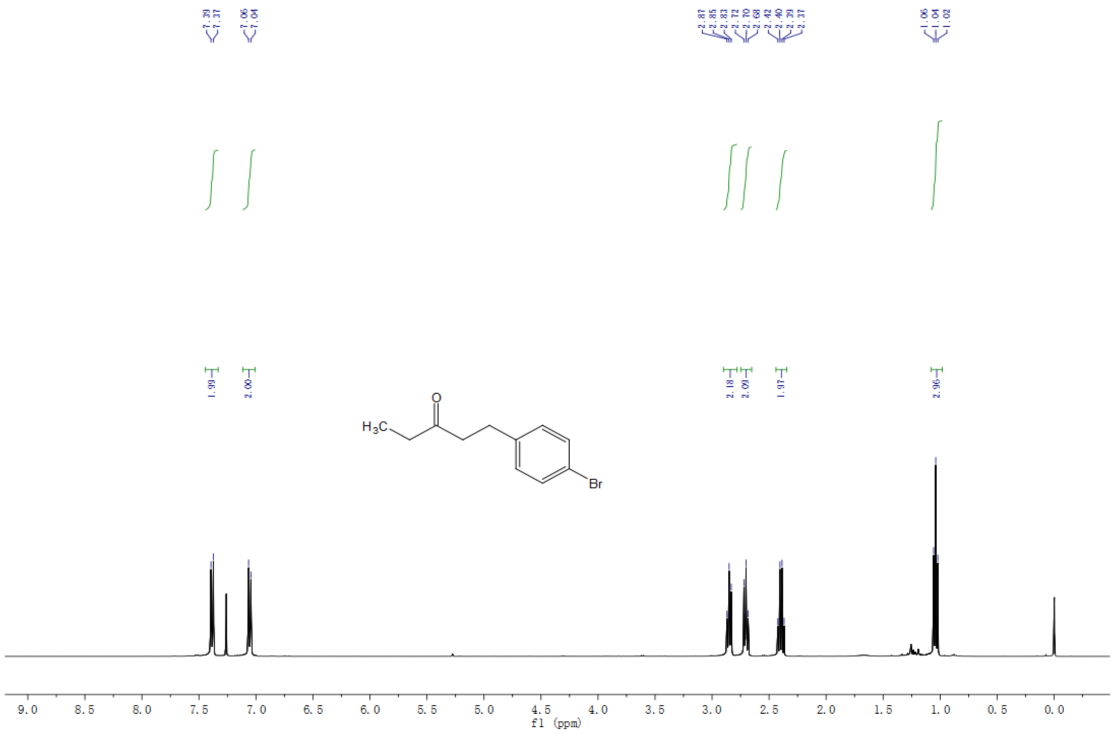


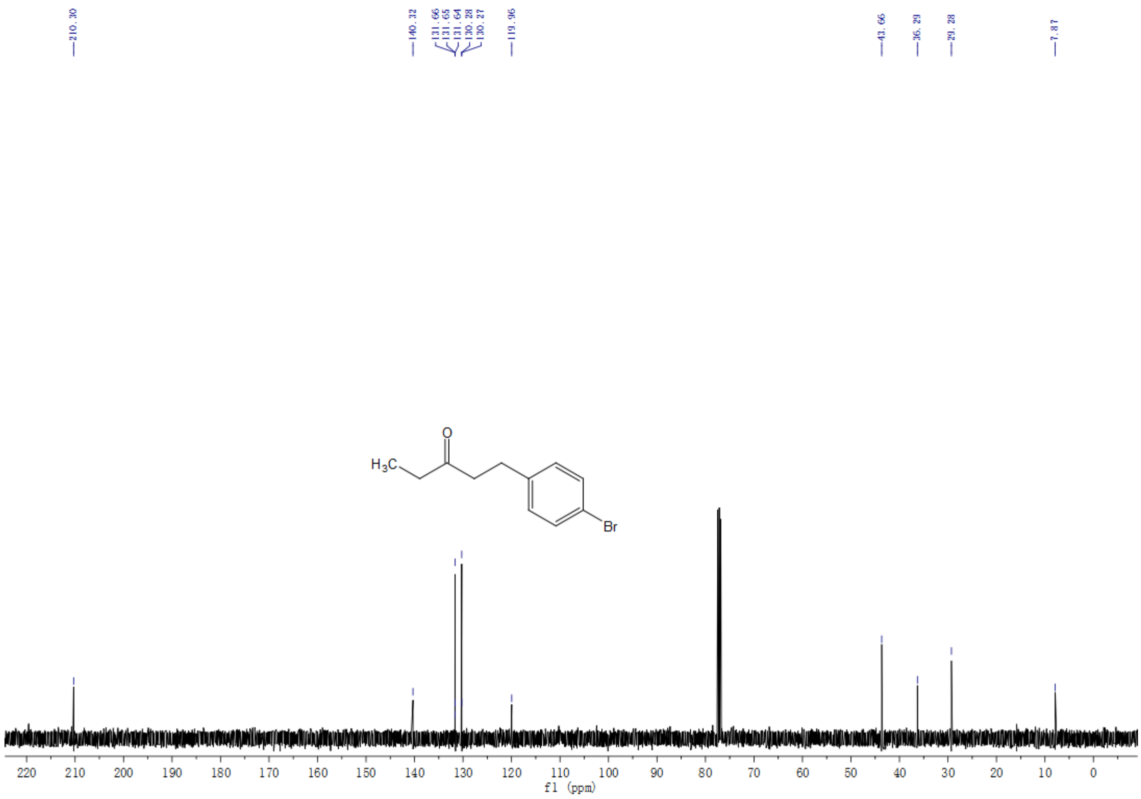


$^{1}$H NMR and $^{13}$C NMR spectra of compound **4cd**

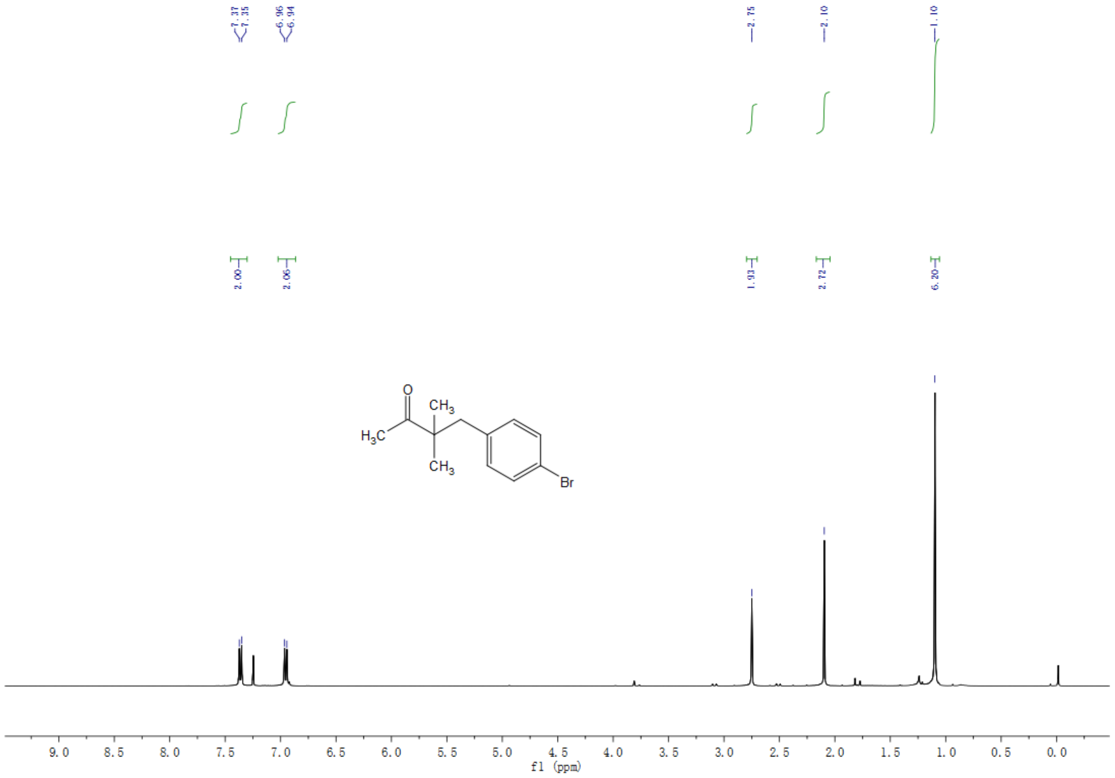


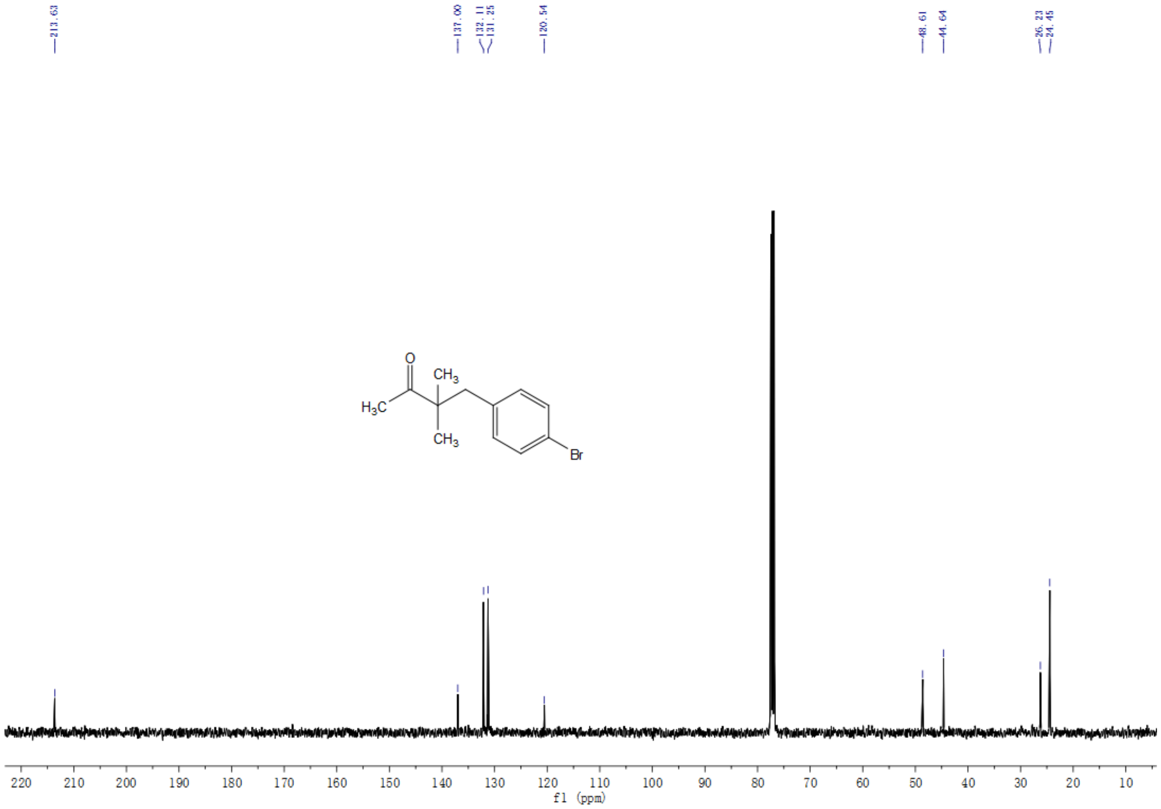


$^{1}H$ NMR and $^{13}C$ NMR spectra of compound **4dd**

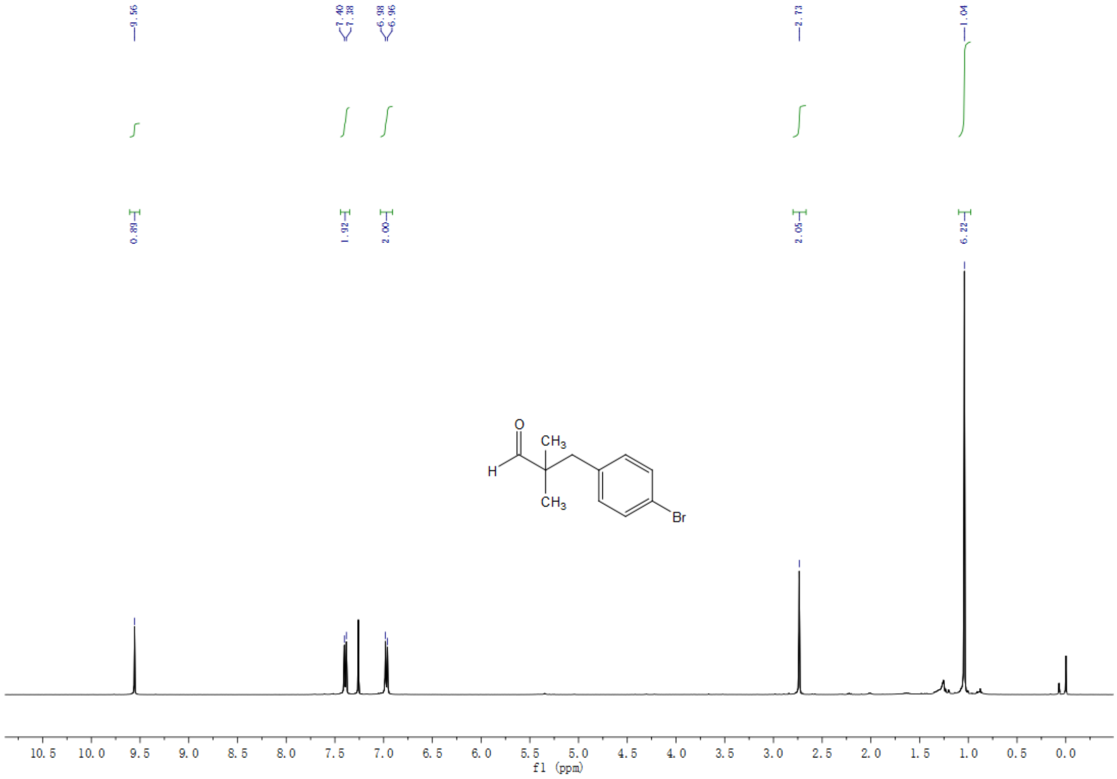


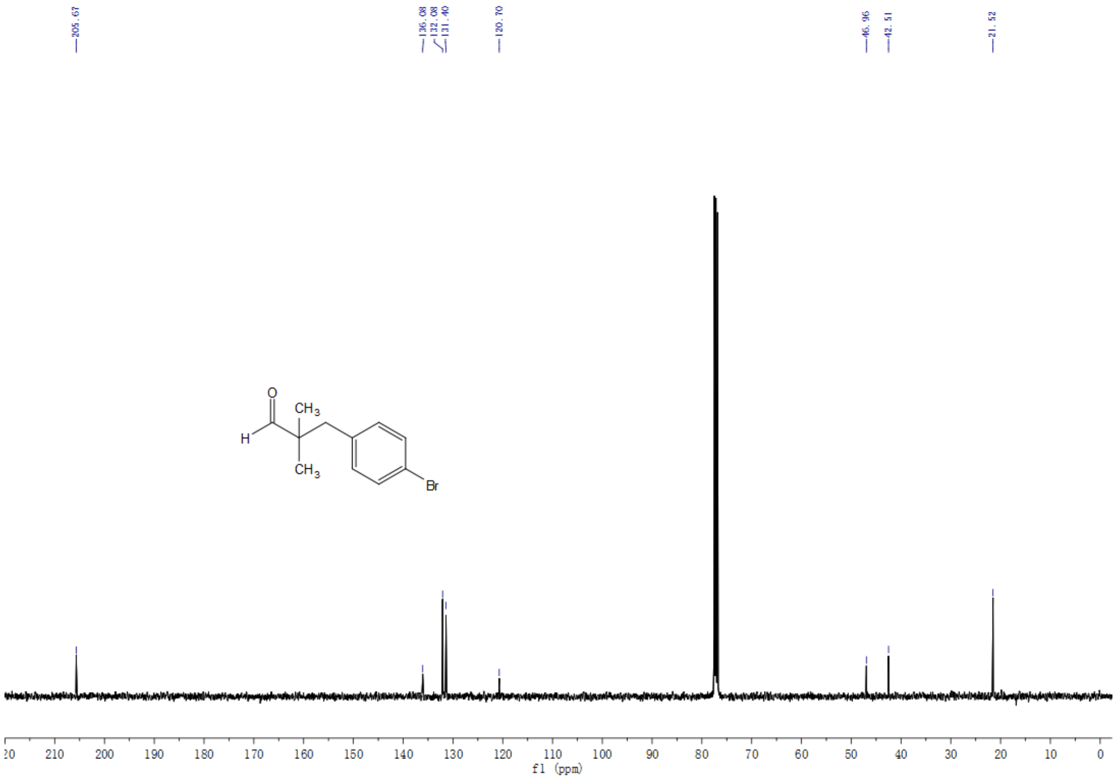


$^{1}$H NMR and $^{13}$C NMR spectra of compound **4ed**

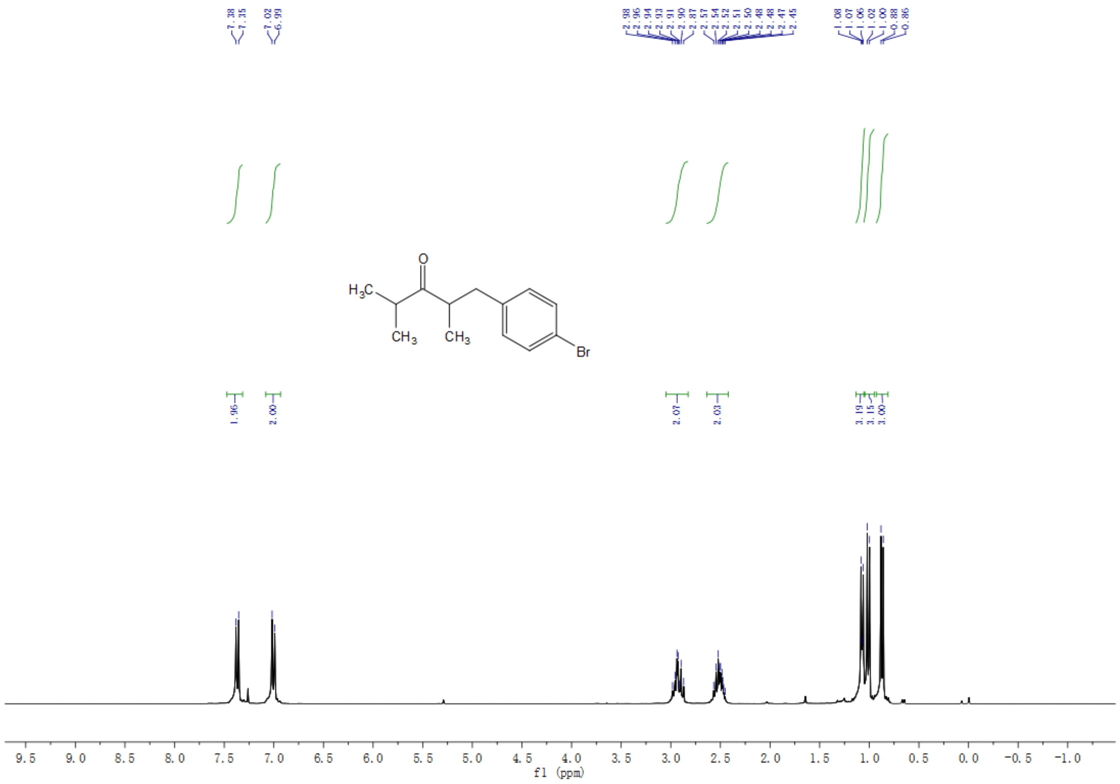


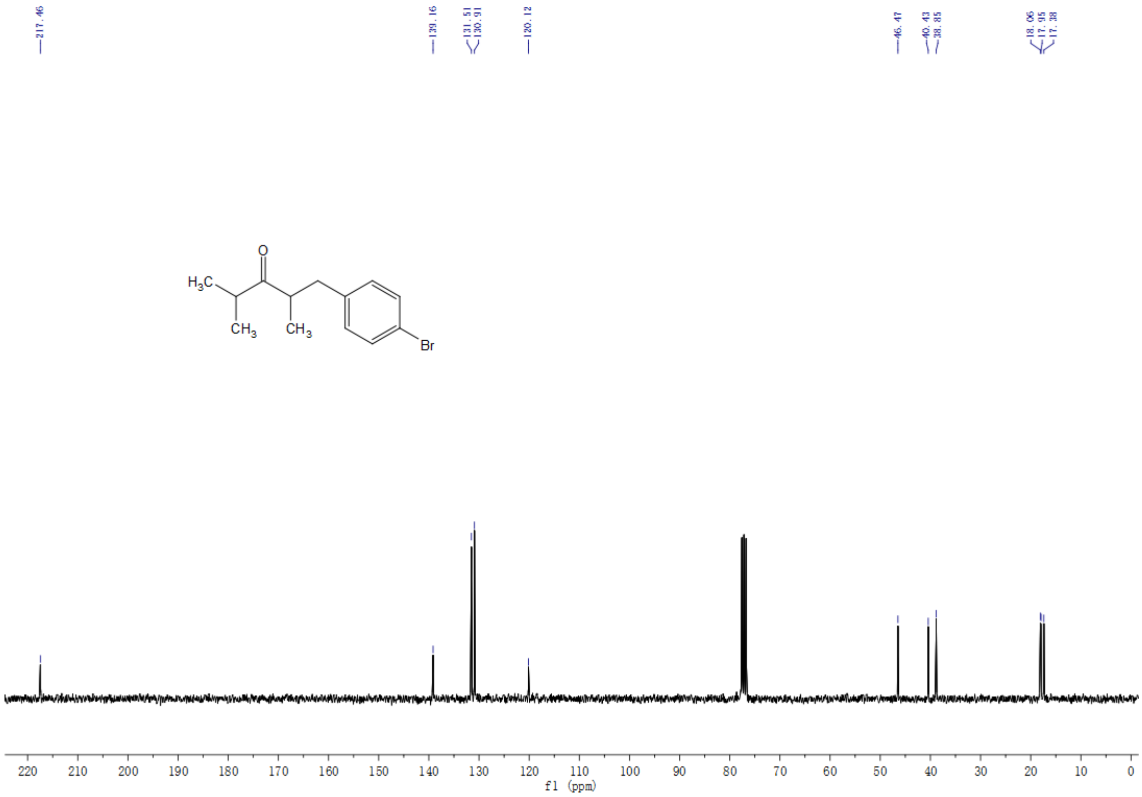


$^{1}$H NMR and $^{13}$C NMR spectra of compound **4fd**

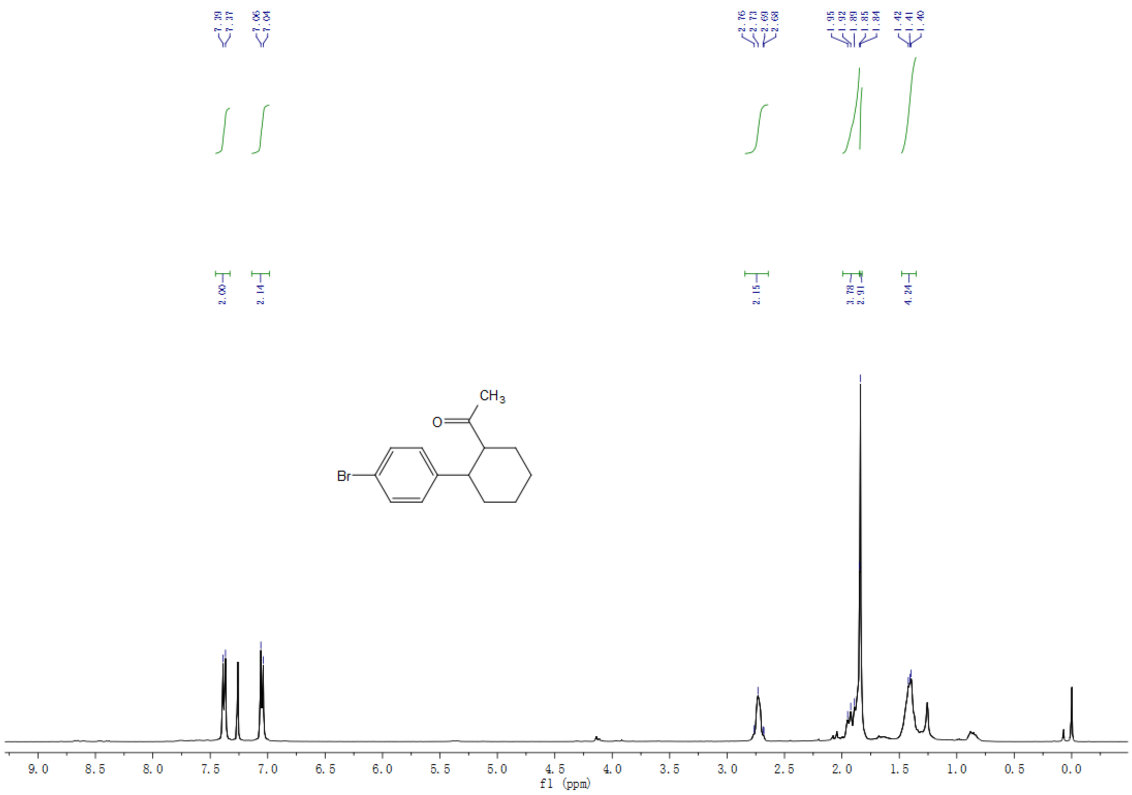

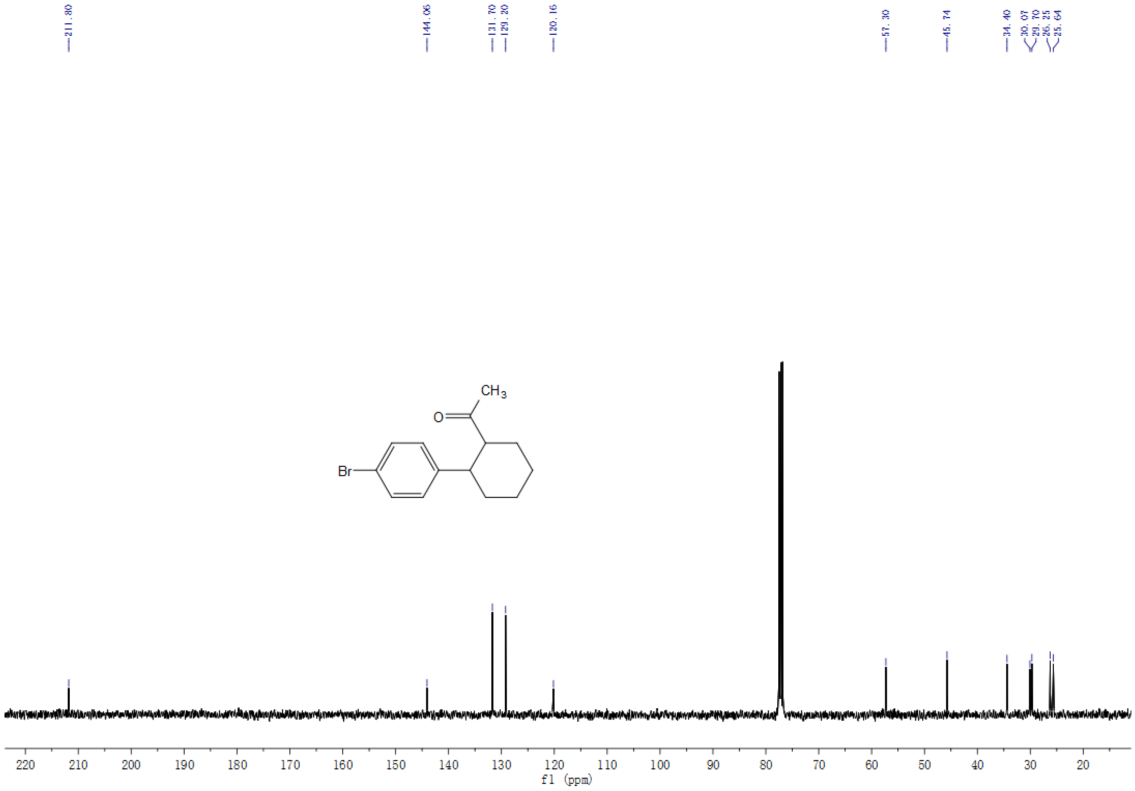

$^{1}H$ NMR and $^{13}C$ NMR spectra of compound **4gd**

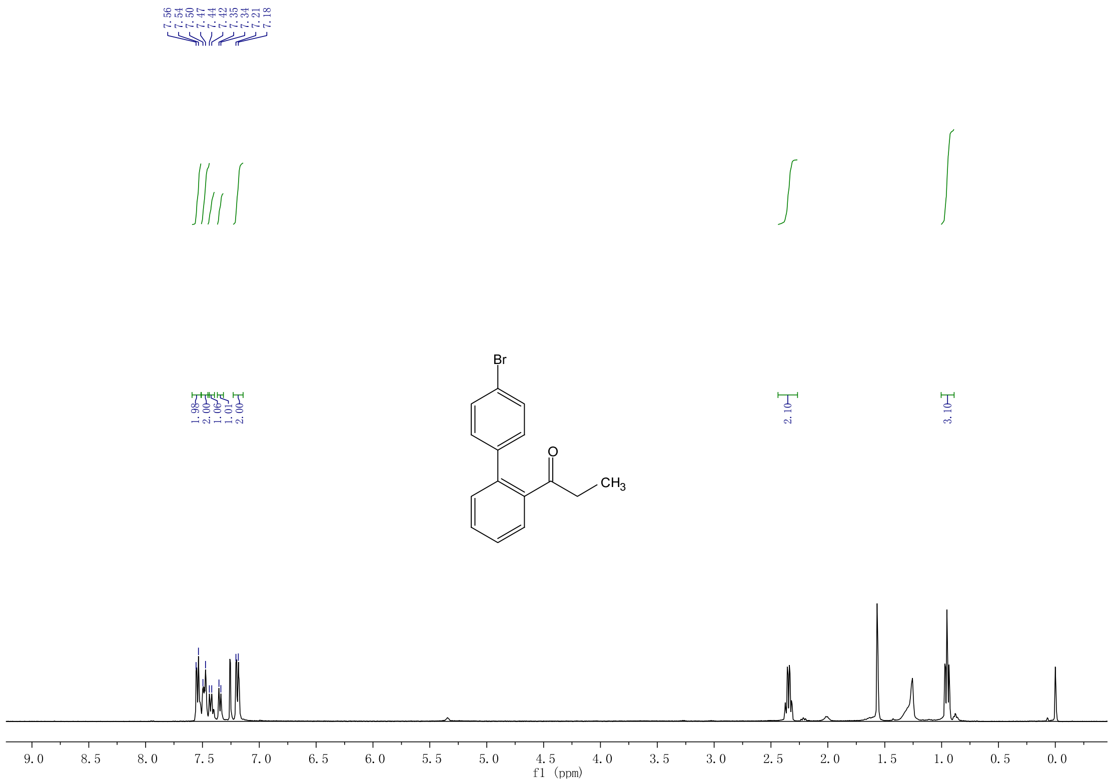

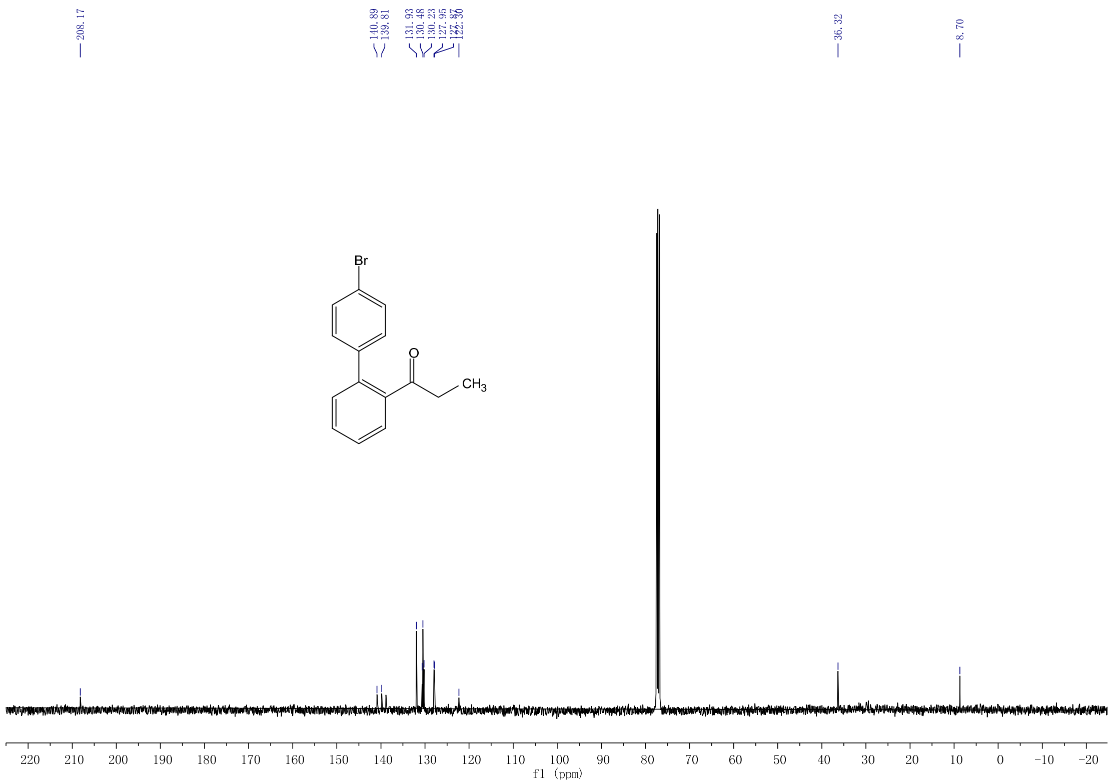

$^{1}$H NMR and $^{13}$C NMR spectra of compound **4od**

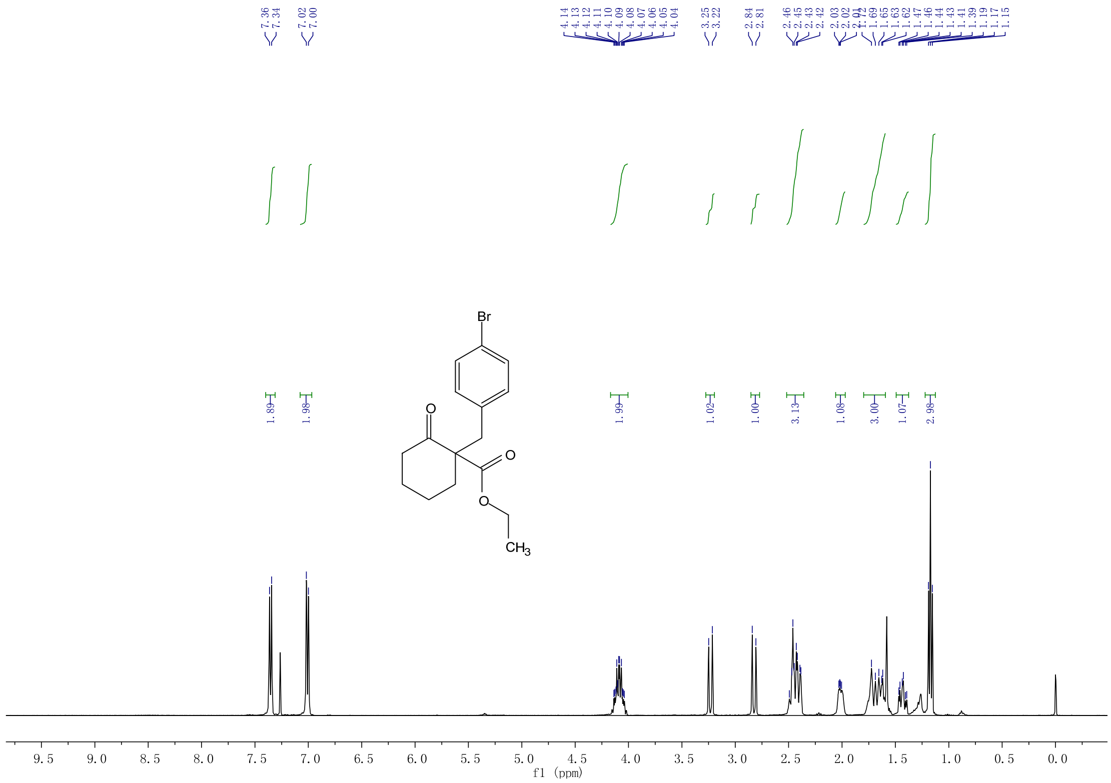

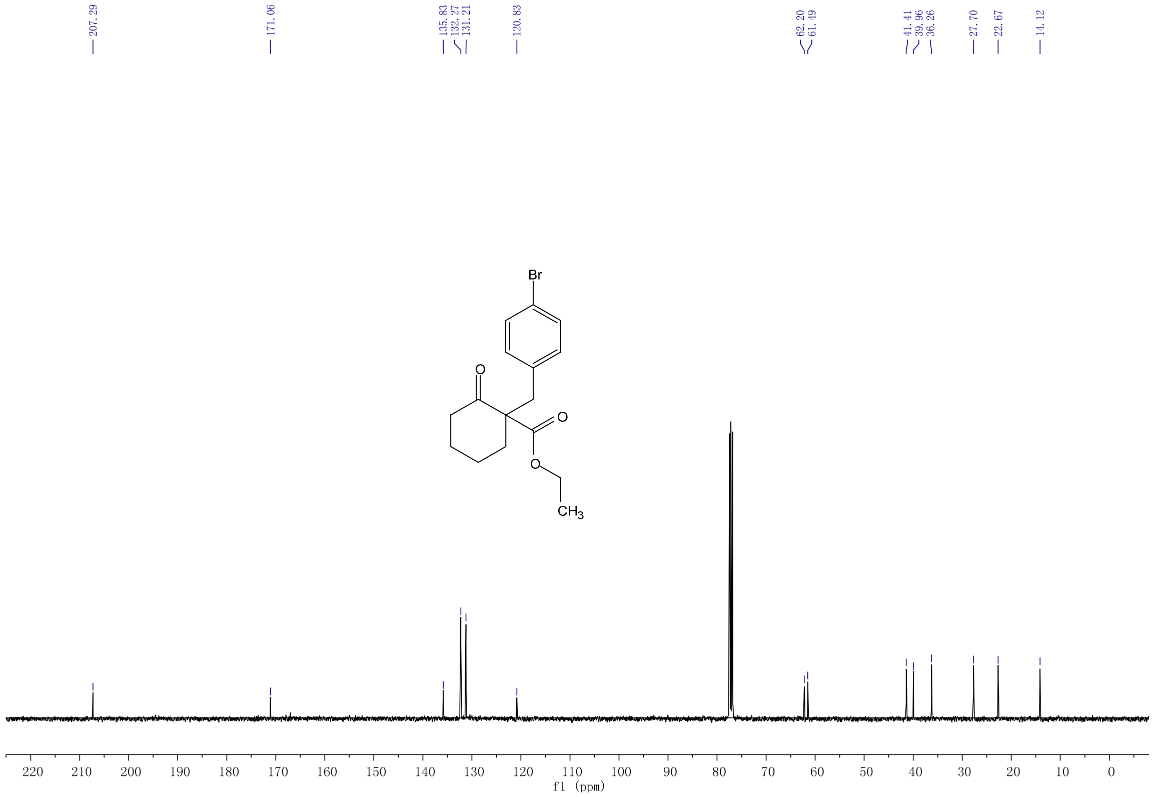

$^1$H NMR and $^{13}$C NMR spectra of compound **4nd**

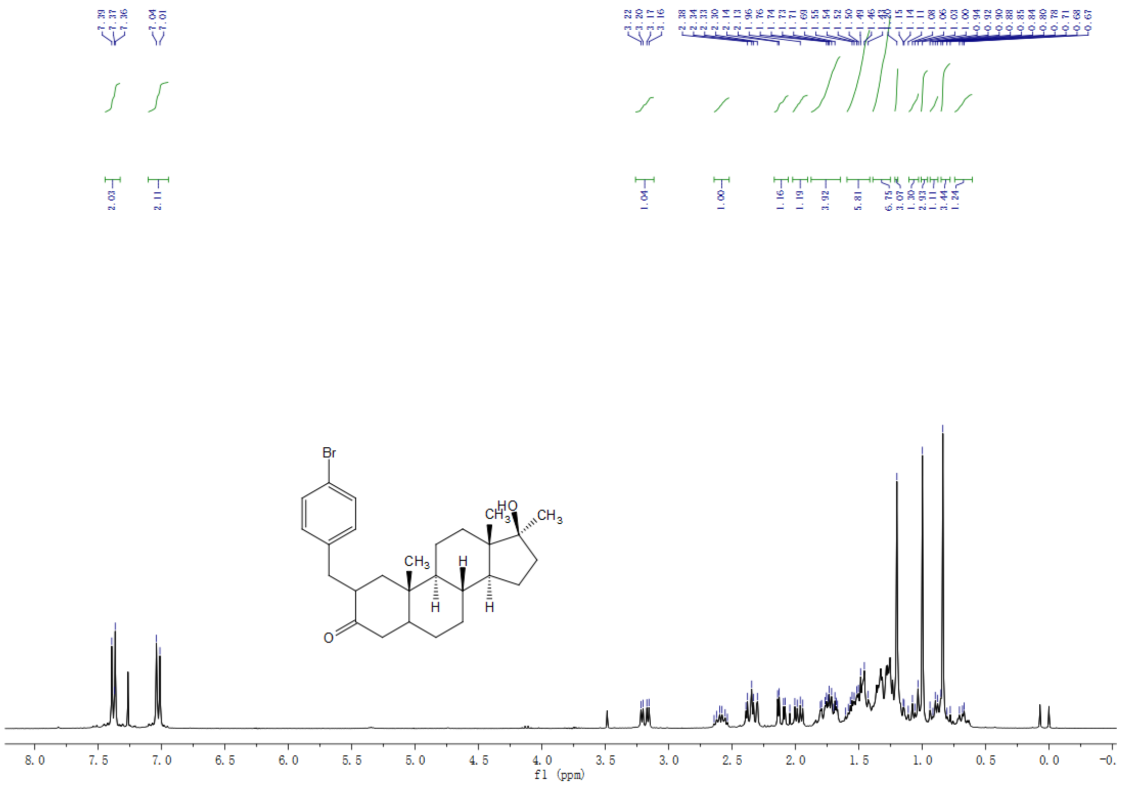

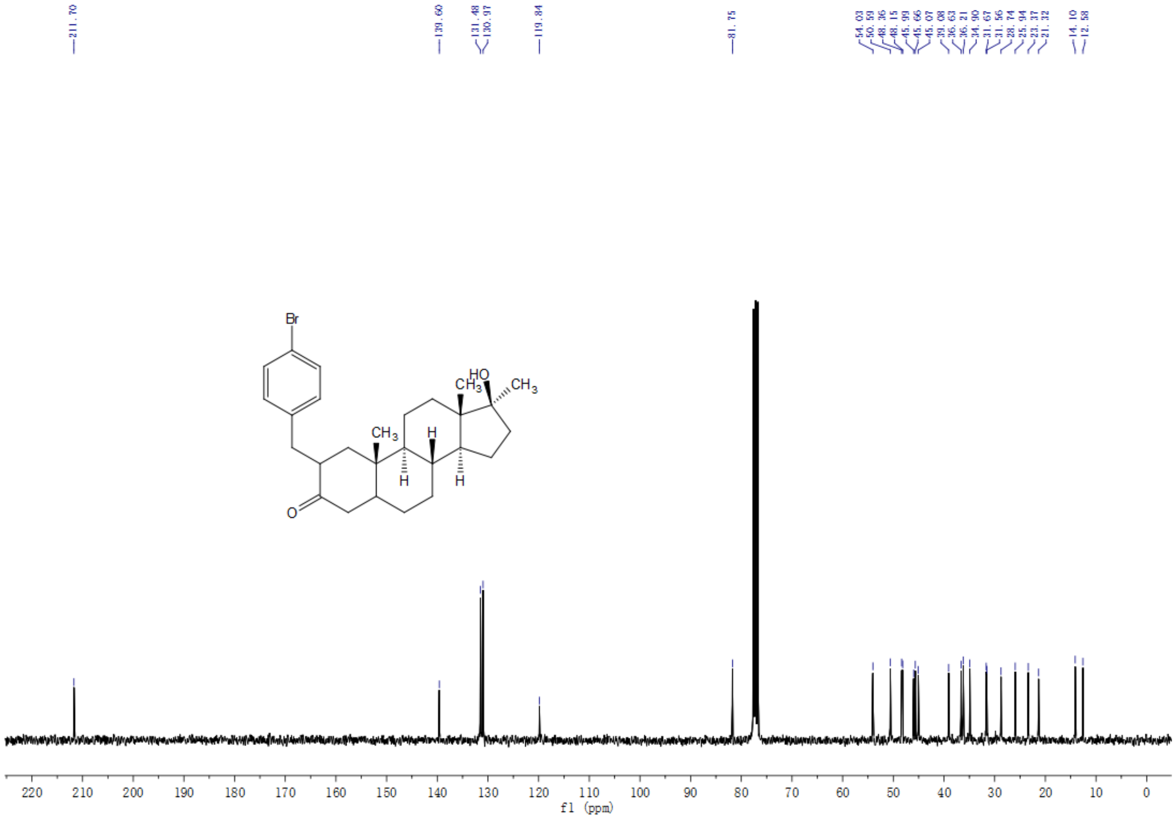

$^{1}$H NMR and $^{13}$C NMR spectra of compound **4md**

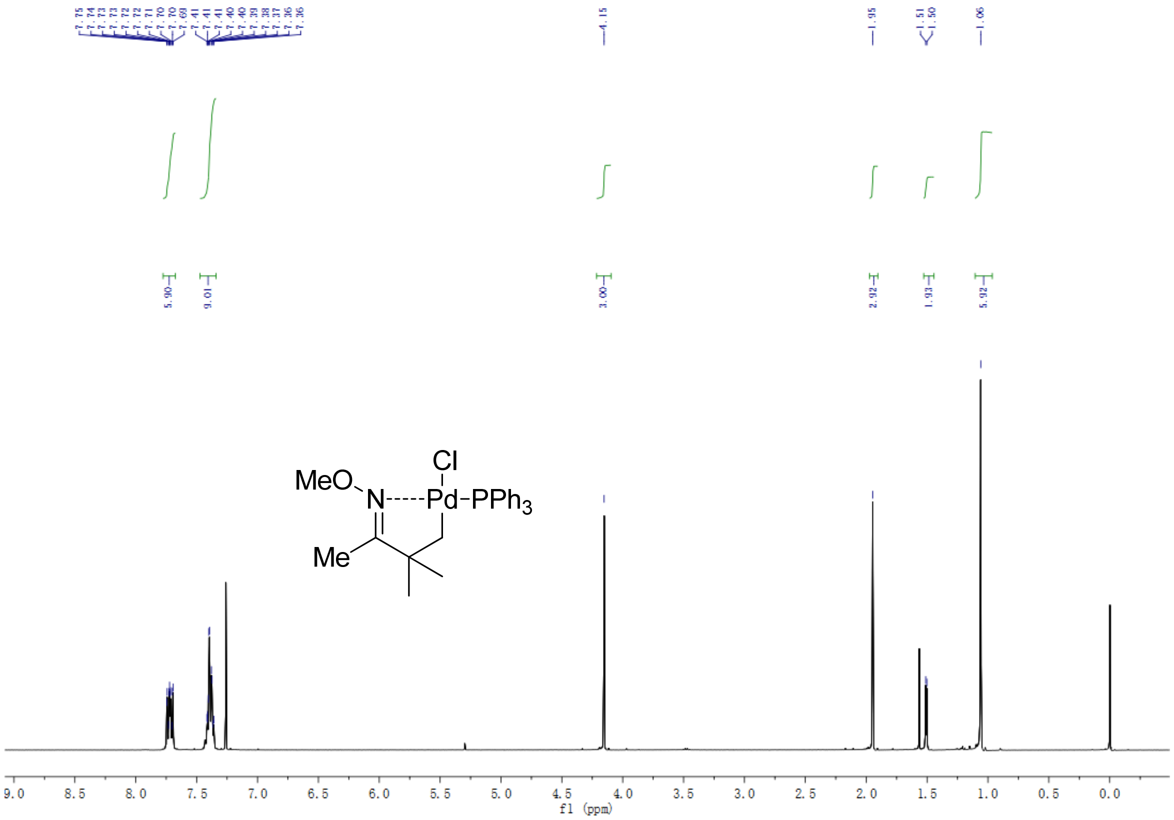


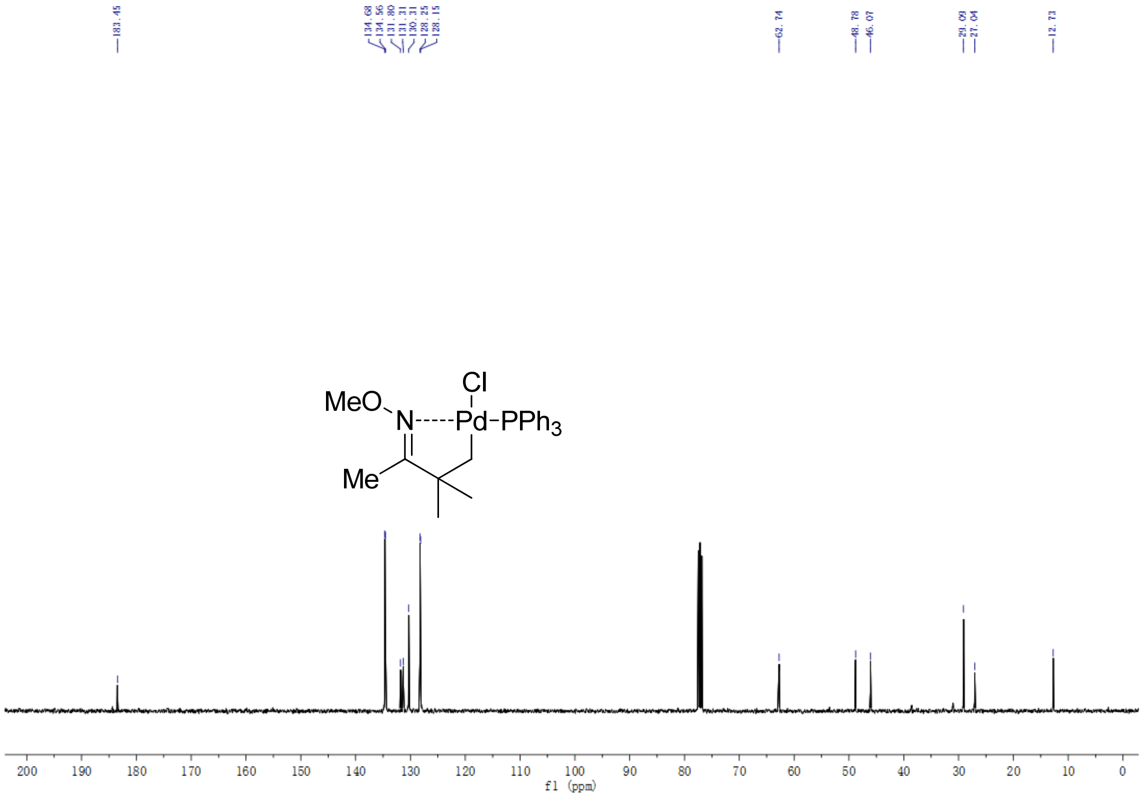


$^{1}$H NMR and $^{13}$C NMR spectra of compound **6**

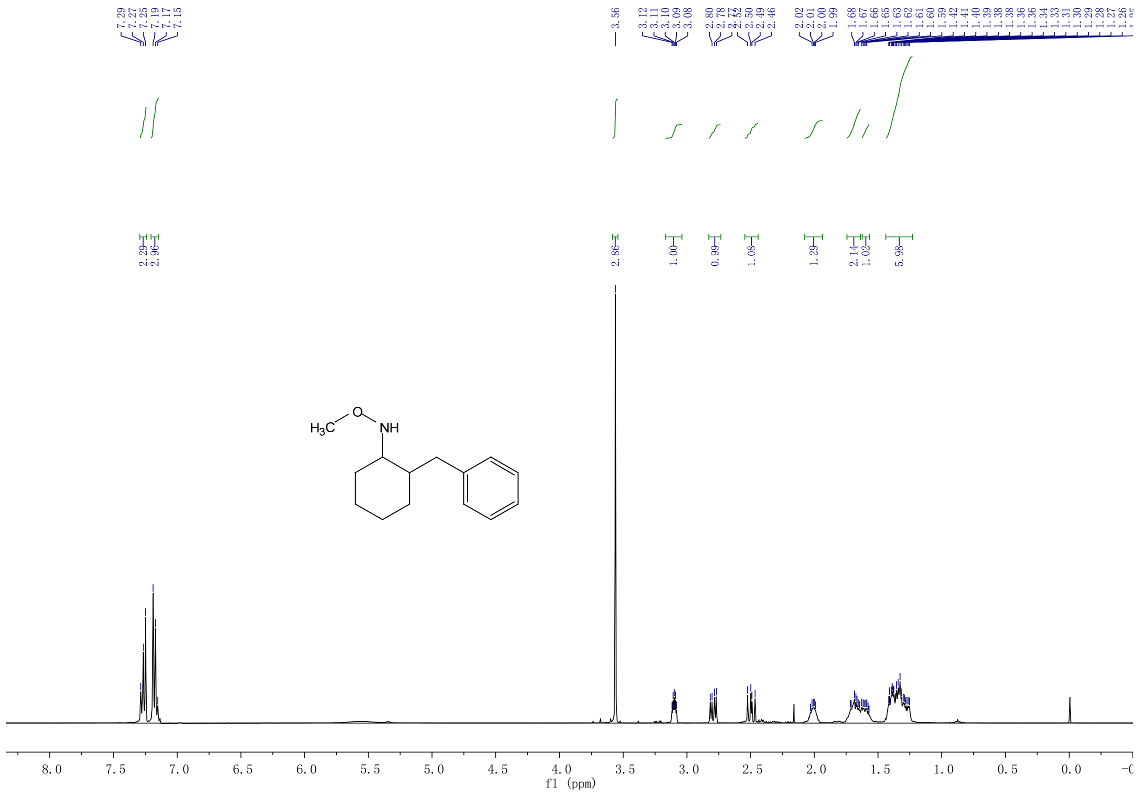


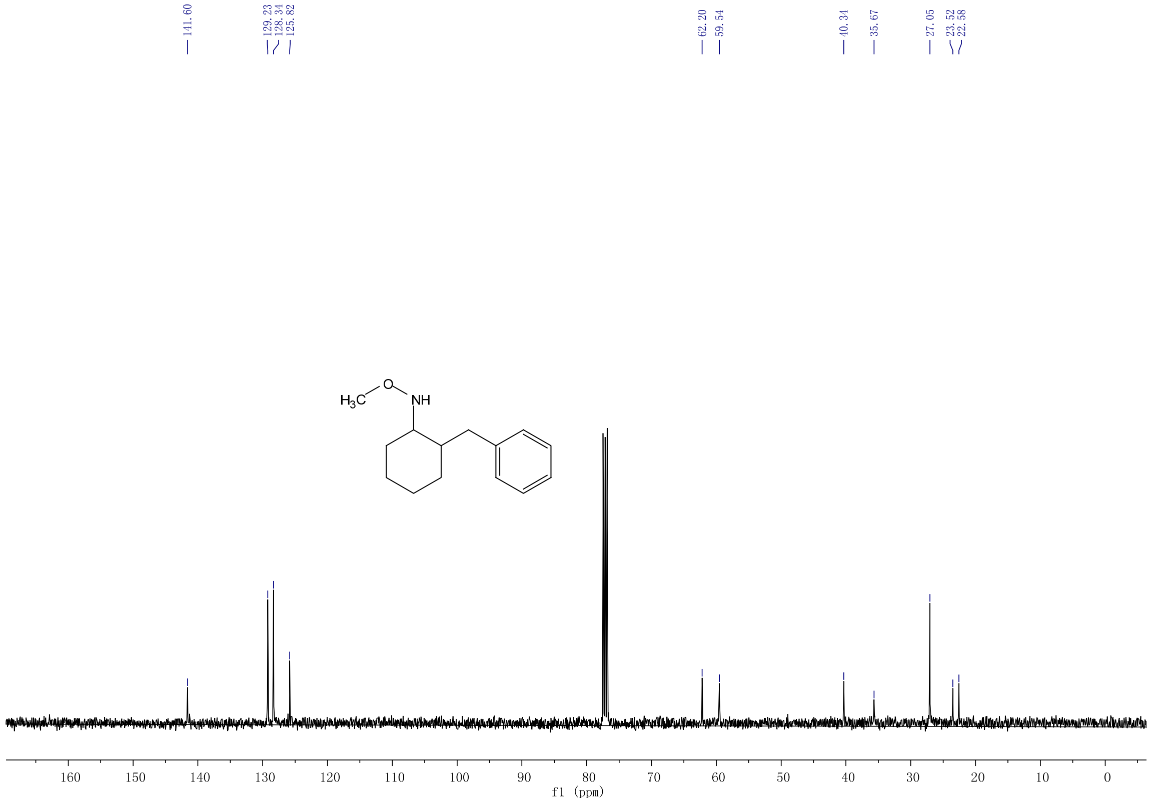


$^{1}$H NMR and $^{13}$C NMR spectra of compound **3aa-H**

# IX. References


[1] a) E. Skucas, D. W. C. MacMillan, *J. Am. Chem. Soc.* **2012**, *134*, 9090; b) M. Bielawski, M. Zhu, B. Olofsson, *Adv. Synth. Catal.* **2007**, *349*, 2610; c) M. Bielawski, B. Olofsson, *Chem. Commun.* **2007**, 2521; d) M. Bielawski, B. Olofsson, *Org. Synth*. **2009**, *86*, 308.

[2] T. Kang, Y. Kim, D. Lee, Z. Wang, S. Chang, *J. Am. Chem. Soc.* **2014**, *136*, 4141.

[3] D. S. William, E. J. Sorensen, *J. Am. Chem. Soc.* **2006**, *128*, 7025.

[4] S. D. Mayer, S. L. Schreiber, *J. Org. Chem.* **1994**, 59, 7549

[5] S. Schwarz, R. Csulk, *Bioorg. Med. Chem.* **2010**, *18*, 7458

[6] R. Subba, P. Kondaiah, S. K. Sinjay, P. Ravanan, M. B. Sporn, *Tetrahedron* **2008**, *64*, 11541

[7] A. McNally, B. Haffemayer, B. S. L. Collins, M. Gaunt, *Nature.* **2014**,*510*, 129.

[8] X. Wang, Xiang, T. Pei, X. –Q. Han, R. A. Widenhoefer, *Org. Lett.* **2003**,*5*, 2699.

[9] O. Mendoza, G. Rossey, L. Ghosez, *Tetra. Lett.* **2010**, *51*, 2571.

[10] W. J. G. M. Peijnenburg, H. M. Buck, *Tetrahedron* **1988**, *44*, 4927.

[11]A.S. Berenblyum, H.A. Al-Waldhaf, O. N. Shishilov, E. M. Evstigneeva, M. H. El-Hussien, V. R. Flid, *Russ. J. Coord. Chem.* **2011**, *37*, 460.
